# Integrative analysis of 3604 GWAS reveals multiple novel cell type-specific regulatory associations

**DOI:** 10.1186/s13059-021-02560-3

**Published:** 2022-01-07

**Authors:** Charles E. Breeze, Eric Haugen, Alex Reynolds, Andrew Teschendorff, Jenny van Dongen, Qing Lan, Nathaniel Rothman, Guillaume Bourque, Ian Dunham, Stephan Beck, John Stamatoyannopoulos, Nora Franceschini, Sonja I. Berndt

**Affiliations:** 1 National Cancer Institute, NIH, Bethesda, MD 20892 USA; 2 Altius Institute for Biomedical Sciences, Seattle, WA 98121 USA; 3 UCL Cancer Institute, University College London, WC1E 6BT, London, UK; 4 CAS Key Laboratory of Computational Biology, CAS-MPG Partner Institute for Computational Biology, Shanghai Institute of Nutrition and Health, Shanghai Institute for Biological Sciences, University of Chinese Academy of Sciences, Chinese Academy of Sciences, 320 Yue Yang Road, Shanghai, 200031 China; 5 Department of Biological Psychology, Vrije Universiteit Amsterdam, Amsterdam, 1081BT The Netherlands; 6 Department of Human Genetics, McGill University and Génome Québec Innovation Center, Montréal, H3A 0G1 Canada; 7 European Molecular Biology Laboratory, European Bioinformatics Institute (EMBL-EBI), Wellcome Genome Campus, Hinxton, Cambridge, CB10 1SD UK; 8 Department of Epidemiology, Gillings School of Global Public Health, University of North Carolina, Chapel Hill, NC USA

## Abstract

**Background:**

Genome-wide association study (GWAS) single nucleotide polymorphisms (SNPs) are known to preferentially co-locate to active regulatory elements in tissues and cell types relevant to disease aetiology. Further characterisation of associated cell type-specific regulation can broaden our understanding of how GWAS signals may contribute to disease risk.

**Results:**

To gain insight into potential functional mechanisms underlying GWAS associations, we developed FORGE2 (https://forge2.altiusinstitute.org/), which is an updated version of the FORGE web tool. FORGE2 uses an expanded atlas of cell type-specific regulatory element annotations, including DNase I hotspots, five histone mark categories and 15 hidden Markov model (HMM) chromatin states, to identify tissue- and cell type-specific signals. An analysis of 3,604 GWAS from the NHGRI-EBI GWAS catalogue yielded at least one significant disease/trait-tissue association for 2,057 GWAS, including > 400 associations specific to epigenomic marks in immune tissues and cell types, > 30 associations specific to heart tissue, and > 60 associations specific to brain tissue, highlighting the key potential of tissue- and cell type-specific regulatory elements. Importantly, we demonstrate that FORGE2 analysis can separate previously observed accessible chromatin enrichments into different chromatin states, such as enhancers or active transcription start sites, providing a greater understanding of underlying regulatory mechanisms. Interestingly, tissue-specific enrichments for repressive chromatin states and histone marks were also detected, suggesting a role for tissue-specific repressed regions in GWAS-mediated disease aetiology.

**Conclusion:**

In summary, we demonstrate that FORGE2 has the potential to uncover previously unreported disease-tissue associations and identify new candidate mechanisms. FORGE2 is a transparent, user-friendly web tool for the integrative analysis of loci discovered from GWAS.

**Supplementary Information:**

The online version contains supplementary material available at 10.1186/s13059-021-02560-3.

## Key findings

- Integrative analysis method spanning multiple epigenomic datasets can link GWAS data to key cell types and tissues
- Analysis reveals novel tissue-specific regulatory associations for 2,057 phenotypes from the GWAS catalogue.
- Redesigned FORGE2 tool can separate previous associations into specific regulatory element classes, such as promoters and enhancers
- Results for H3K27me3 and H3K9me3 suggest novel role for repressed regions in GWAS-mediated disease mechanisms

## Background

Over the past two decades, Genome-Wide Association Studies (GWAS) have emerged as one of the most important frameworks to improve our understanding of the contribution of genetics to a wide variety of complex diseases and traits [1, 2]. As of 2019, over 3000 GWAS have been conducted, uncovering over 128,000 associations between genomic variants and phenotypes, as indicated by the GWAS catalogue [3]. However, despite the explosion in the number of studies, the interpretation of GWAS findings has remained challenging, as many GWAS SNPs localise to non-protein-coding variants [4].

One way to aid the interpretation of GWAS results is by integrating these with large-scale epigenomic mapping data generated by projects like ENCODE, Roadmap Epigenomics, and BLUEPRINT [5–7]. In 2012, ENCODE scientists demonstrated that by integrating DNase-seq data from large-scale epigenomic mapping projects with disease-associated GWAS variants one can systematically categorise and localise key disease-associated cell types, tissues, and regulatory regions [4]. Further research by the Roadmap Epigenomics consortium developed and expanded upon this concept by analysing epigenetic data across 111 epigenomes [6]. This analysis approach is useful in understanding functional mechanisms underlying genetic loci as it can provide key information to identify the temporal- and tissue-specific actions of particular regulatory elements, as evidenced by recent studies on the FTO locus [8], and the complement component 4 locus [9]. In these efforts, the prioritisation and integrative analysis of specific target GWAS regions with epigenomic mark and/or gene expression data has proved crucial for understanding and designing further laboratory experiments to determine function.

Although integrative analysis can offer an approach for pinpointing both systems-level and locus-specific disease-associated regulation, implementations of these types of analyses are not without their pitfalls, as appropriate statistical analysis and background correction methods are needed to accurately prioritise and categorise GWAS variants. To address this problem and provide a robust methodology for scientists conducting GWAS, we developed the Functional element Overlap analysis of the Results of GWAS Experiments (FORGE) tool in 2015 [10]. This tool provides an automated web framework for systematic analysis of GWAS findings, focusing on DNase I hotspot data from ENCODE and Roadmap Epigenomics projects [5, 6].

Over the past few years, there has been an explosion in epigenomic data tracks, including ChIP-seq, DNase-seq and hidden Markov model (HMM) chromatin state tracks. When integrated with GWAS results, this new generation of data can offer further insight into the functional mechanisms of genetic loci prioritised by GWAS and help discriminate causal SNPs from other nearby genetic variants. However, these new epigenomic data can be difficult to access and integrate into GWAS analyses. There is a need to provide an updated, user-friendly and comprehensive framework for the categorisation, analysis and prioritisation of GWAS loci based on regulatory elements, in order to facilitate the identification of associated regulatory pathways, drug targets, cis-regulatory elements and cell types/tissues. To address this need, we developed FORGE2, a new statistical framework and analysis tool, which expands upon FORGE and incorporates new epigenomic data tracks, including DNase I hotspots, histone mark broadPeaks and HMM chromatin state tracks. The expanded atlas of cell type-specific regulatory element annotations in FORGE2 provides additional information that can be used to further refine and understand loci from GWAS. To demonstrate the breadth of information that can be gained from FORGE2, we analysed GWAS results from 3,604 phenotypes (GWAS phenotypes), representing a broad range of diseases and traits, downloaded from the NHGRI-EBI GWAS catalogue. We present results across DNase I hotspots, five histone mark categories and 15 HMM chromatin states and examine the relationship between associations among these different marks.

## Results

### FORGE2 analysis of GWAS results reveals multiple novel tissue-specific associations with DNase I hotspots

**Fig. 1:**
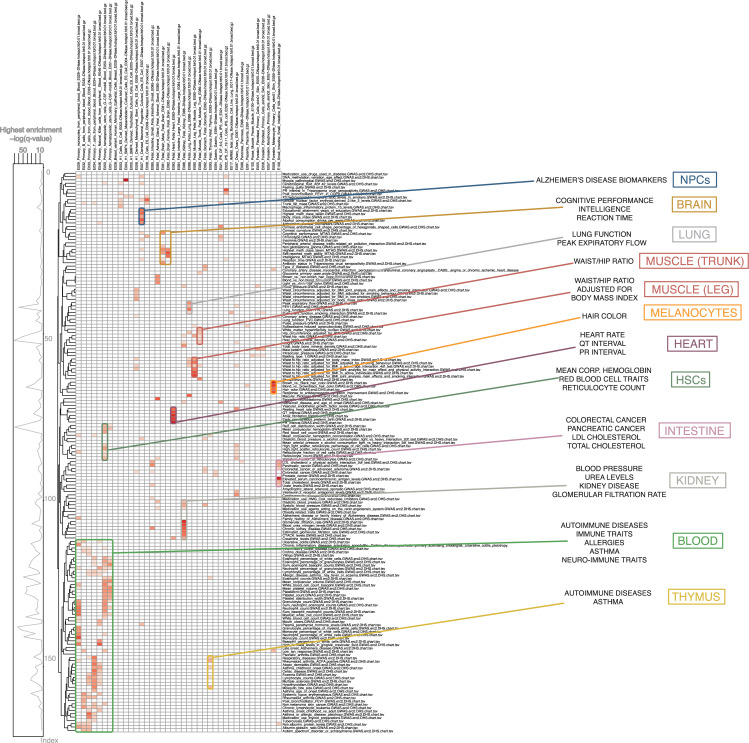
**GWAS enrichments for DNase I hotspots from consolidated Roadmap Epigenomics consortium data:** Shown are significant tissue-specific enrichment results (*q* value < 0.01, Benjamini-Hochberg (BH) correction) for FORGE2 analysis across the NHGRI/EBI GWAS catalogue (downloaded 2 September 2019). GWAS phenotypes (rows) are clustered using complete linkage clustering (Euclidean distance), while different tissues and cell types (columns) are grouped according to related tissue or cell type categories (e.g. white blood cell categories are placed together, as are fibroblast categories, etc.). Values are row-normalised, with reference lineplot on the left indicating top enrichment value for each category. Clustering reveals groups of related phenotypes with similar tissue-specific enrichment profiles, such as immune traits for blood or cognitive performance for brain. Groups are highlighted for different tissue- and cell type-enrichments, each in a different colour (right panel). A zoomable version of this figure can be found at: https://www.easyzoom.com/imageaccess/797f4272e4674bb28b42adf3f1918cf7

To gain an understanding of the relationship between GWAS findings and regulatory regions, we first focused on DNase-seq data. DNase-seq data are used to map DNase I hypersensitive sites (DHSs), which are regions of chromatin sensitive to cleavage by the DNase I enzyme [11]. DHSs are classic markers of regions of regulatory DNA, covering many types of cis-regulatory elements (CREs) including promoters, enhancers, silencers, insulators and locus control regions [12]. We used DNase-seq data from ENCODE, BLUEPRINT and Roadmap Epigenomics (2012 and 2015 data releases) to identify DNase I hotspots, which are similar to DHSs but encompass broader regions of DNase hypersensitivity [10]. We examined the association between DNase I hotspots and results from 3604 GWAS from the GWAS catalogue with FORGE2 (Additional File 1: Figure S1). We observed significant tissue- and cell type-specific enrichment for DNase I hotspots for 293 phenotypes from the GWAS catalogue (*q* value < 0.01) (Fig. 1, Additional File 1: Figures S2-S4, Additional Files 2, 3, 4 and 5: Tables S1-S4). Clustering analysis of the enrichment results revealed the existence of groups of phenotypes with significant associations for specific tissues or cell types. In many cases, the cell- or tissue-specific enrichment observed was consistent with the known aetiology of the phenotype. For example, this analysis showed that GWAS findings for QT interval were enriched for DNase I hotspots in heart tissue, whereas GWAS results for red blood cell traits were enriched for DNase I hotspots in haematopoietic stem cells. Several of these associations were previously known, but we uncovered many new associations suggesting underlying biological mechanisms, including, among others, for hair colour, which was enriched for melanocyte DNase I hotspots, and for waist circumference, which was enriched for mesenchymal stem cell DNase I hotspots.

Thus, we demonstrated that FORGE2 can systematically link a wide variety of GWAS associations to DNase I hotspots (or accessible chromatin elements) specific to particular tissues and cell types, providing extensive analytical confirmation of previous findings [4], and also revealing previously unreported links between GWAS phenotypes and epigenomic marks for certain cell types and tissues.

### FORGE2 analysis of GWAS results reveals shared cell type-specific enrichments for different histone marks

DNase I hotspot enrichments may be driven by variants located in different classes of CREs, such as promoters and enhancers, all of which are in accessible chromatin. Particular histone modifications (e.g. H3K4me3) more closely associated with specific CRE classes (such as promoters). These posttranslational modifications of histone proteins are known to influence processes as diverse as gene regulation, DNA repair and chromosome condensation [13]. Among the many types of histone modifications, specific histone modifications are enriched for certain types of genomic annotations such as enhancers (H3K4me1), promoters (H3K4me3), transcribed regions (H3K36me3), heterochromatin regions (H3K9me3), and polycomb-repressed regions (H3K27me3) [6]. Enrichment of GWAS loci in these regions could provide additional information regarding mechanisms underlying GWAS associations, potentially at a cell type-specific level. Using ChIP-seq data and computed broadPeak regions for the aforementioned 5 histone marks for 39 cell types from the Roadmap Epigenomics project [6] in FORGE2, we investigated the associations between these marks and GWAS catalogue results (Additional File 1: Figures S5-S10, Additional Files 6, 7, 8, 9 and 10: Tables S5-S9).

**Fig. 2:**
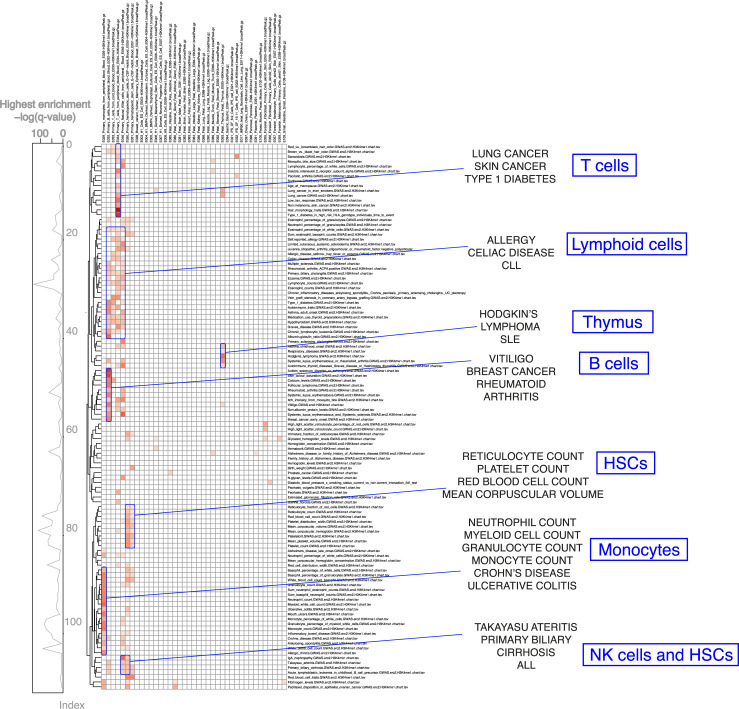
**GWAS enrichments for immune cell H3K4me1 broadPeaks from consolidated Roadmap Epigenomics consortium data:** shown are significant cell type-specific enrichment results (*q* value < 0.01, BH correction) for FORGE2 analysis across the NHGRI/EBI GWAS catalogue (downloaded 2 September 2019). GWAS phenotypes (rows) are clustered using complete linkage clustering (Euclidean distance), while different tissues and cell types (columns) are grouped according to related tissue or cell type categories (e.g. T cell categories are placed together, as are haematopoietic stem cell categories, etc.). All sample categories, including non-immune cell categories, are shown. Values are row-normalised, with reference lineplot on the left indicating top enrichment value for each category. Clustering reveals grouping of related phenotypes with similar immune cell type-specific enrichment profiles. We highlighted several groups including known related phenotypes such as mean corpuscular volume for HSCs, monocyte count for monocytes and rheumatoid arthritis for B cells (right panel). A zoomable version of this figure can be found at: https://www.easyzoom.com/imageaccess/e8c2fd02c90f456b85f629635c2f30a3

As a proof of principle, we first sought to verify if we could detect cell type-specific associations using histone mark data from immune cells, as immune cells are some of the best characterised cell types of any organ or system. We focused on H3K4me1 for this analysis, because it is the most tissue- and cell type-specific of the 5 main histone marks assayed by the Roadmap Epigenomics consortium [6]. Unsupervised analysis of the GWAS catalogue revealed enrichment for immune cell type-specific H3K4me1 broadPeaks for specific immune phenotypes (Fig. 2). Clustering analysis of the observed enrichments revealed segregation of the immune phenotypes into different immune cell classes, such as T cells and B cells. For example, GWAS data for monocyte cell count were enriched for H3K4me1 marks in monocytes, and chronic lymphocytic leukaemia (CLL) data were enriched for H3K4me1 marks in lymphoid cells. Thus, we demonstrate that FORGE2 can be used to identify cell type-specific histone associations with GWAS loci, improving the resolution to dissect the cell type-specific contribution to complex traits and diseases.

**Fig. 3:**
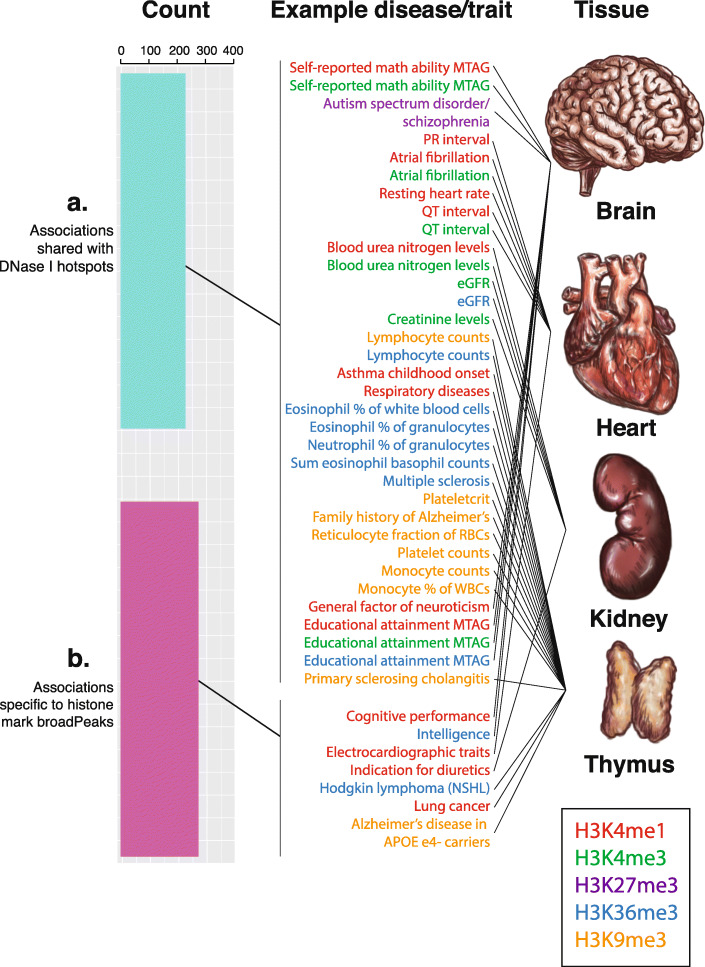
**Shared and distinct tissue-specific enrichments for histone mark broadPeaks from consolidated Roadmap Epigenomics consortium data:** (**A**) 231 tissue-specific enriched phenotypes from histone mark broadPeaks are shared with the set of enriched phenotypes for DNase I hotspot data for the same tissues, including examples for brain (central panel, self-reported math ability -MTAG-, H3K4me1 and H3K4me3 enrichment), heart (atrial fibrillation, H3K4me1 and H3K4me3 enrichment), kidney (estimated glomerular filtration rate or eGFR, H3K4me3 and H3K36me3 enrichment), and thymus (family history of Alzheimer’s, H3K9me3 enrichment). (**B**) 265 tissue-specific enriched phenotypes from histone mark broadPeaks are not shared with the set of enriched phenotypes for DNase I hotspot analysis for the same tissues, including examples for brain (central panel, cognitive performance H3K4me1 enrichment), heart (electrocardiographic traits, H3K4me1 enrichment), and thymus (nodular sclerosing Hodgkin lymphoma or NSHL H3K36me3 enrichment). PR interval (P wave to initiation of QRS complex interval), QT interval (Q wave to end of T wave interval), RBCs (red blood cells), WBCs (white blood cells), MTAG (multi-trait analysis of GWAS), NSHL (nodular sclerosis Hodgkin's lymphoma), Apolipoprotein E allele E4 (APOE e4). Tissue images used here are from Roadmap Epigenomics Consortium et al., 2015 [6]. A zoomable version of this figure can be found at: https://www.easyzoom.com/imageaccess/196e07438d2f48f6b24cb378d65f31b7

For certain phenotypes, analyses integrating histone mark data could reveal new cell type-specific enrichments which are not found by analysing DNase I hotspots, as a different set of genomic regions are used. We evaluated the extent to which tissue- or cell type-specific enrichments for the 5 core histone marks assayed by the Roadmap Epigenomics consortium (comprising H3K4me1, H3K4me3, H3K27me3, H3K36me3, and H3K9me3) were shared with enrichments observed for DNase I hotspots across the GWAS catalogue. We uncovered tissue- or cell type-specific enrichment for at least one histone mark for 496 traits/diseases. Of these 496 phenotypes, 231 also showed enrichment in DNase I hotspot analysis of the GWAS catalogue (Fig. 3A); however, 265 did not show enrichment in DNase I hotspot analysis of the GWAS catalogue and thus were specific to histone mark broadPeaks, suggesting that additional information about GWAS data may be gained from integrating information for specific histone marks (Fig. 3B).

To understand how tissue-specific enrichments were shared between the 5 histone marks analysed (described more extensively in Additional File 1: Figure S10), we performed a comparative analysis of histone mark enrichments in a representative subset of our data, specifically 4 tissues representative of 4 of the major systems in the human body which were most extensively sampled (brain, heart, kidney and thymus, representative of the nervous, cardiovascular, urinary, and immune systems). When analysing these data, we discovered that, for some phenotypes, the tissue-specific association was only present for one histone mark, whereas for other phenotypes, tissue-specific associations were observed for multiple histone marks (Fig. 3, central panel). For example, GWAS results for atrial fibrillation (which were previously found to be enriched for DNase-seq data in heart tissue) were enriched for both H3K4me1 (a mark associated with enhancers) and H3K4me3 (a mark associated with promoters) in heart tissue, indicating that a subset of the variants associated with this phenotype likely exert their effect on phenotype by affecting gene regulation, and specifically by affecting enhancer and promoter elements.

Further exploration of the overlap in histone mark enrichments revealed several notable patterns. Certain groups of phenotypes seem to consistently associate with the same type of histone mark in the same tissue. For example, a number of immune cell traits, such as eosinophil percentage of white blood cells, sum of eosinophil/basophil counts and neutrophil percentage of granulocytes, display a significant H3K36me3 enrichment in thymus, indicating that a subset of the variants associated with these phenotypes are located in regions of active transcription in the thymus. This suggests that genomic variation for certain classes of phenotypes may have a disproportionate effect on specific genomic region classes (in this case actively transcribed regions) in a tissue-specific context, hinting towards a potential systems effect involving common underlying biological processes or pathways. Some phenotypes individually point to a disproportionate effect of GWAS variants on one of the CRE classes. For example, PR interval, an electrocardiographic trait, shows a heart-specific enrichment solely for H3K4me1, indicating an association with heart-specific enhancer elements. Other phenotypes show tissue-specific enrichment across multiple histone marks, suggesting that the underlying variants exert their effects on gene regulation through several different classes of active CRE, such as enhancers and promoters (e.g. an H3K4me1 and H3K4me3 heart enrichment for atrial fibrillation). We also observed a number of tissue-specific associations for histone marks associated with repressive regions, such as a brain-specific H3K27me3 enrichment for “autism spectrum disorder (or schizophrenia)”, or a thymus-specific H3K9me3 enrichment for primary sclerosing cholangitis. These formerly unreported tissue-specific enrichments for repressive histone marks hint towards the possibility that GWAS SNPs may also have tissue-specific effects in repressive regions.

### FORGE2 chromatin state analysis of GWAS detects associations across different tissue-specific regulatory element classes

Although examination of histone marks can yield information about GWAS findings, additional information can be gained by evaluating the combination of multiple marks, which can inform segmentations of chromatin known as chromatin states. These chromatin states can further refine classes of genomic or regulatory elements, such as promoters, enhancers, or repressed regions, as well novel classes or subclasses of elements [14]. We evaluated the relationship between GWAS data and 15-state Roadmap Epigenomics ChromHMM chromatin segmentations, comprising the following states: active transcription start site -TSS- (TssA), flanking active TSS (TssAFlnk), bivalent/poised TSS (TssBiv), strong transcription (Tx), transcription at 5′ and 3′ ends of a gene (TxFlnk), weak transcription (TxWk), zinc finger (ZNF) genes and repeats (ZNF-Rpts), flanking bivalent TSS/enhancer (BivFlnk), enhancer (Enh), bivalent enhancer (EnhBiv), genic enhancer (EnhG), heterochromatin (Het), quiescent/low (Quies), repressed polycomb (ReprPC), and weak repressed polycomb (ReprPCWk) across 127 cell types [6].

**Fig. 4:**
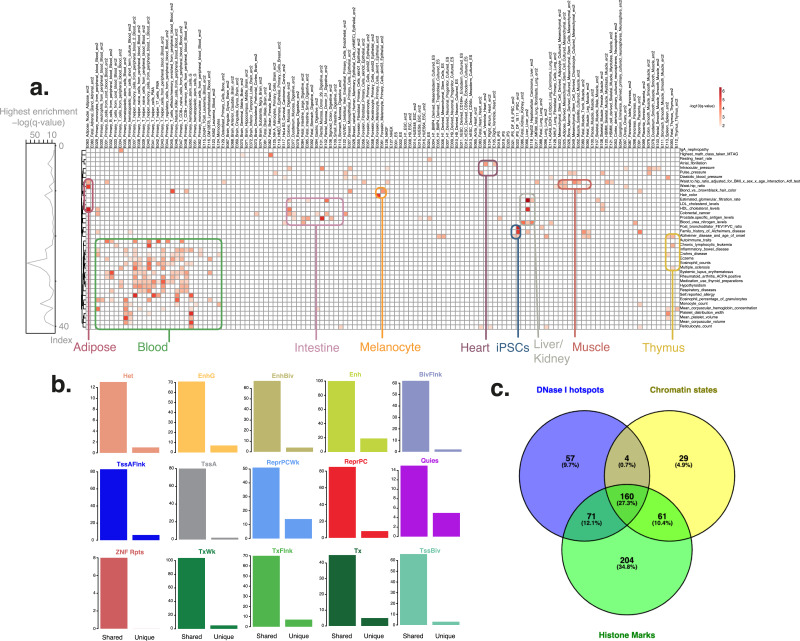
**GWAS enrichments for enhancer (Enh) HMM chromatin state regions from consolidated Roadmap Epigenomics consortium data:** (**A**) Shown are significant tissue-specific enrichment results (*q* value < 0.01, BH correction) for a representative random subset with 40 phenotypes from FORGE2 analysis across the NHGRI/EBI GWAS catalogue (downloaded 2 September 2019, full set shown in Additional File 1: Figure S19). GWAS phenotypes (rows) are clustered using complete linkage clustering (Euclidean distance), while different tissues and cell types (columns) are grouped according to related tissue or cell type categories (e.g. white blood cell categories are placed together, as are fibroblast categories, etc.). Values are row-normalised, with reference lineplot on the left indicating top enrichment value for each category. Clustering reveals grouping of related phenotypes with similar tissue-specific enrichment profiles. We highlighted several groups from each tissue, including phenotypes such as self-reported allergy for blood cells, atrial fibrillation for heart tissue or high-density lipoprotein (HDL) cholesterol levels for liver. (**B**) Number of shared vs unique (non-shared) tissue-specific enrichment profiles for different GWAS phenotypes across 15 chromatin states. (**C**) Venn diagram showing number of shared and unique (non-shared) phenotypes between DNase-seq datasets (DNase I hotspots), chromatin states and histone mark broadPeak datasets. The largest category is unique enriched phenotypes for histone mark broadPeaks (204, 34.8%), followed by shared enriched phenotypes across all three categories (160, 27.3%). A zoomable version of this figure can be found at: https://www.easyzoom.com/imageaccess/9e3b9bab699148f586db26ad36ba0002

Analysing the 3,604 GWAS from GWAS catalogue revealed enrichment in chromatin states for 254 phenotypes, of which 225 also showed enrichment in DNase I hotspot or histone marks; 29 phenotypes did not show enrichment in DNase I hotspot or histone mark broadPeak analysis of the GWAS catalogue and thus were specific to chromatin states (Fig. 4C, Additional File 1: Figures S11-S25, Additional File 11: Table S10). The number of enriched phenotypes was different across chromatin states. Active chromatin states such as active TSS or enhancer displayed a higher number of associated enriched GWAS phenotypes, whereas repressed region chromatin states, such as ZNF-repeats, quiescent, or heterochromatin, showed a lower number of enriched phenotypes.

The chromatin state for which we observed the highest number of enriched phenotypes (*n*=119) was the enhancer (Enh) state. To understand the phenotypes associated with this class further, we clustered enhancer-associated phenotypes according to their tissue-specific signal. Figure 4A shows a representative random subset with 40 phenotypes (full set shown in Additional File 1: Figure S19). These results highlight clusters of phenotypes converging on particular tissues or cell types, such as autoimmune traits and immune cell enhancers, or HDL cholesterol levels and liver enhancers, and show that FORGE2 can be used to link GWAS loci to specific chromatin states, systematically uncovering tissue-specific effects in common traits and diseases.

A large proportion of the enriched phenotypes that we observed for the enhancer chromatin state were shared with other chromatin states (100 out of 119). In order to get a general view of shared vs non-shared enriched phenotypes across chromatin state classes we categorised chromatin state results into two categories: unique for a particular state, or shared with other states (Fig. 4B). This revealed a high proportion of shared enrichments across chromatin states, especially for active TSS, ZNF-repeats, flanking bivalent TSS/enhancer, and bivalent/poised TSS.

To further categorise shared and unique chromatin state-associated phenotypes, we conducted set intersection analyses via the R package UpSetR [15]. We uncovered a range of different levels of shared and unique chromatin state associated phenotypes (Additional File 1: Figure S26). Interestingly, some phenotypes such as waist to hip ratio presented enrichment across several chromatin states, while other phenotypes such as autoimmune traits or interleukin-1-receptor antagonist levels presented enrichment only for specific chromatin states (enhancer and weak repressed polycomb, respectively). Shared associations between chromatin state classes were more frequent if chromatin states belonged to related categories, e.g. bivalent/poised TSS (TssBiv), flanking bivalent TSS/enhancer (BivFlnk) and bivalent enhancer (EnhBiv) categories, which suggests potentially related biological mechanisms underlying these different enrichments.

### Comparative analysis of FORGE2 results reveals shared associations between different epigenomic marks

**Fig. 5:**
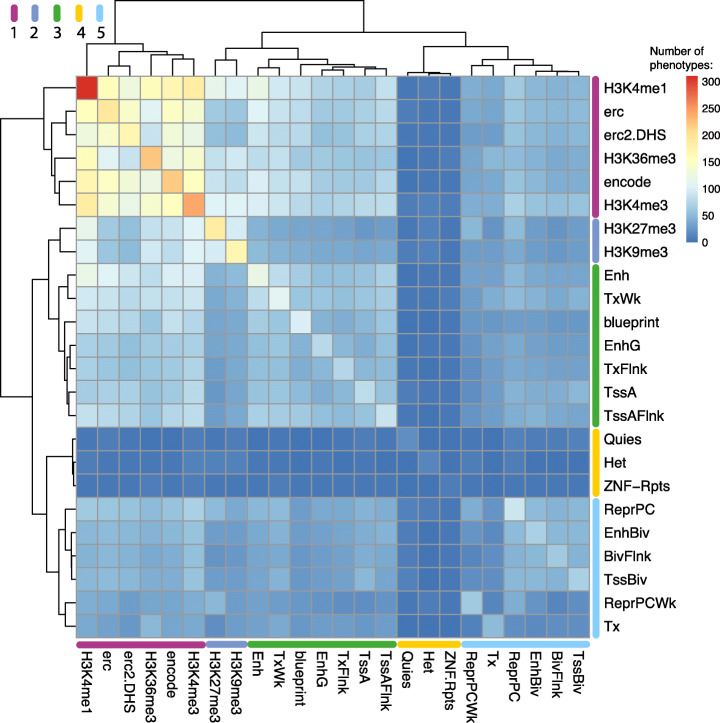
**Relationship between enriched GWAS phenotypes for different epigenomic marks from consolidated Roadmap Epigenomics consortium data:** heatmap showing the number of tissue-specific enriched phenotypes (*q* value < 0.01, BH correction) for FORGE2 analysis across the GWAS catalogue for DNase I hotspots, histone mark broadPeaks and HMM chromatin states. Epigenomic mark categories are clustered using complete linkage clustering (Euclidean distance), across rows and columns. Clustering reveals 5 main groups of epigenomic marks, coloured in purple (group 1), dark blue (group 2), green (group 3), yellow (group 4), and light blue (group 5). A zoomable version of this figure can be found at: https://www.easyzoom.com/imageaccess/4e114698194f492faa1d6812d325cd30

In this study, we have analysed GWAS data across multiple epigenomic datasets. However, it is important to consider that these datasets are interrelated. For instance, each one of these datasets presents a specific relationship with CREs. Chromatin regions accessible to the DNase I enzyme are representative of all main classes of CREs, while certain histone marks are enriched for different categories of CREs (e.g. H3K4me1 is enriched for enhancer regions). HMM chromatin state tracks constitute a highly refined dataset providing further granularity with classes and subclasses of genomic regions. Given the interrelationship between DNase I hypersensitive regions, histone mark broadPeaks and HMM chromatin state regions, we sought to compare the FORGE2 results across these different data types. Clustering of the FORGE2 results revealed large-scale substructure with segregation into 5 main groups, ranked on the number of associated GWAS phenotypes identified by each group (Fig. 5).

Group 1 included the most tissue-specific mark, H3K4me1, which had the highest number of associated phenotypes of any single mark (*n*=310), as well as DNase I hotspots from the ENCODE and Roadmap Epigenomics projects, H3K36me3 (a transcription-associated mark), and H3K4me3 (a promoter-associated mark). This group had the highest number of associated phenotypes (*n*=479), reinforcing the concept that active regions of chromatin associated with promoters, enhancers and transcribed elements represent the main category of regions associated with tissue-specific GWAS mechanisms.

Group 2 contained a lower number of unique associated phenotypes (n=254) and comprised two histone marks, H3K27me3 (a polycomb-repressed region associated mark), and H3K9me3 (a heterochromatin associated mark). These histone mark regions, which are known to be associated with either constitutively repressed regions forming heterochromatin, or regions subject to binding by the polycomb repressive complex 2 (PRC2), displayed enrichments for a markedly different set of phenotypes when compared to regions of active chromatin, suggesting the potential for additional understanding of GWAS SNP disease-associated mechanisms in these regions.

Group 3 had the third highest number of associated phenotypes (*n*=230) and included active chromatin states, such as enhancer (Enh), flanking active TSS (TssAFlnk), and DNase I hotspots from BLUEPRINT. Although many of these chromatin state categories showed enrichment for fewer GWAS phenotypes on average, a number of a number of these categories included smaller regions, and were more closely associated with specific classes of regulatory elements. The BLUEPRINT project focused on immune-associated cell types, and the resulting data constitute a reduced part of the overall human tissue accessible chromatin register. These datasets may be particularly valuable when evaluating the functional genomics for GWAS of immune-related traits.

Group 4 included constitutively repressed chromatin states, such as heterochromatin (Het), quiescent (Quies), and ZNF genes and repeats (ZNF-Rpts). This group represents the lowest number of associated phenotypes (*n*=36), consistent with the fact that GWAS SNPs are less likely to co-localise with repressed regions of chromatin than with active regions, especially if these are well segmented. However, it is important to highlight that analysis of repressed and heterochromatic regions suggests that these regions are not completely exempt from mechanisms mediated through disease-associated GWAS variants.

Group 5 had the second lowest number of associated phenotypes (*n*=173) and included polycomb-repressed chromatin states (e.g. repressed polycomb, ReprPC), and bivalent chromatin states (e.g. bivalent/poised TSS, TssBiv), and transcribed regions (e.g. “strong transcription”, Tx). These results suggest that bivalent and polycomb-repressed chromatin states play a role in some GWAS variant-associated mechanisms and that their role may be more prominent than that of heterochromatic regions. Some classes of repressed regions may be more informative than others. Interestingly, among the phenotypes associated with polycomb-repressed regions, we observed GWAS for neurodegenerative diseases, such as Alzheimer’s disease, as having enrichment for repressed polycomb (ReprPC) in brain as the top associated tissue. This finding highlights this regulatory class for GWAS loci of Alzheimer’s disease, which involves a loss of neurons and synapses across the cerebral cortex and specific subcortical regions [16]. These observations are a first step in evaluating and quantifying the different levels of importance of regulatory region classes in the context of interpreting biological mechanisms for GWAS-associated variants.

### Example analysis of myeloproliferative neoplasm data illustrates FORGE2 application in cancer GWAS

**Fig. 6:**
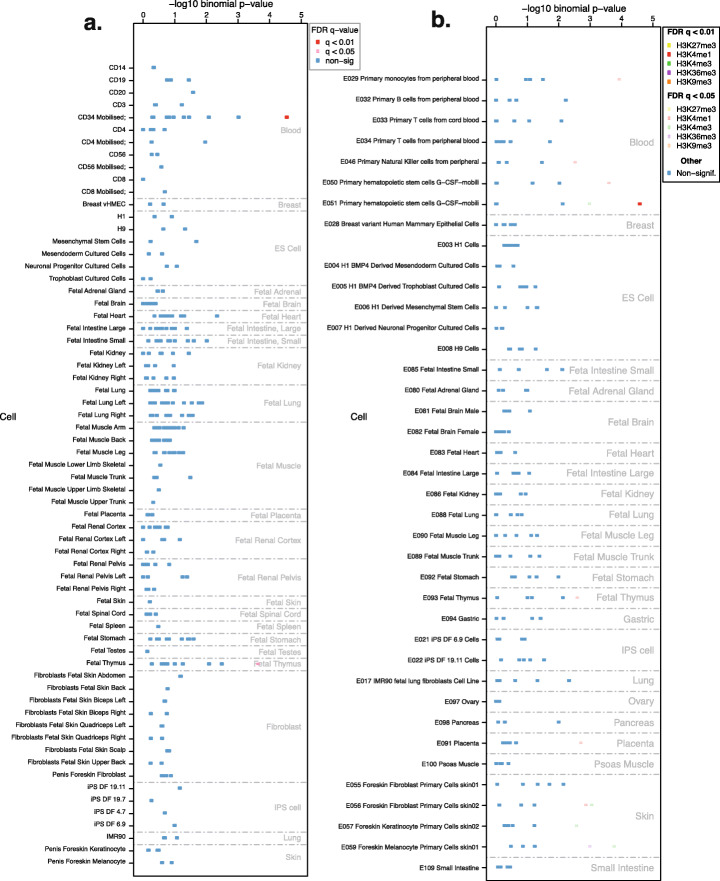
**Myeloproliferative neoplasm GWAS enrichment for DNase I hotspots and consolidated histone mark broadPeaks from Roadmap Epigenomics consortium data:** (**A**) Significant tissue- and cell type-specific enrichment results (*q* value < 0.01, Benjamini-Hochberg (BH) correction) for FORGE2 DNase I hotspot analysis on 25 suggestive MPN GWAS associations (*p* value < 1 × 10^−6^). Different tissues and cell types (rows) are grouped according to related tissue or cell type categories (e.g. white blood cell categories are placed together, as are fibroblast categories, etc.). FORGE2 DNase I hotspot analysis reveals a cell type-specific enrichment profile for CD34+ haematopoietic stem cells (red dot). (**B**) Significant tissue- and cell type-specific enrichment results (*q* value < 0.01, Benjamini-Hochberg (BH) correction) for FORGE2 histone mark broadPeak analysis for the same variants. Different tissues and cell types (rows) are grouped according to related tissue or cell type categories (e.g. white blood cell categories are placed together, as are fibroblast categories, etc.). Here, FORGE2 analysis reveals a cell type-specific enrichment profile for CD34+ haematopoietic stem cell H3K4me1 broadPeaks, a mark enriched for enhancer regions (red dot). A zoomable version of this figure can be found at: https://www.easyzoom.com/imageaccess/3245f515d6894a8eb644b35ea22b2ef5

To demonstrate the utility of FORGE2 for individual GWAS, we used data from a recent GWAS on myeloproliferative neoplasms or MPNs [17], not yet included in the GWAS catalogue at the time of initial analysis. This study reported a list of variants with suggestive associations with MPNs (*p* value < 10^−6^) [17]. FORGE2 analysis of these variants with DNase I hotspot data revealed a cell type-specific enrichment for CD34+ haematopoietic stem cells (HSCs, Fig. 6A). Subsequent evaluation with histone mark data revealed a significant tissue-specific association for haematopoietic stem cell-specific H3K4me1 broadPeaks (Fig. 6B). This indicates that genomic variants that predispose to myeloproliferative neoplasms tend to co-localise with haematopoietic stem cell-specific CREs marked by DNase I hotspots, and more specifically haematopoietic stem cell-specific enhancer regions marked by H3K4me1 broadPeaks. This finding echoes other associations we detected for blood cancers including a haematopoietic stem cell (HSC) H3K4me1 broadPeak enrichment for acute lymphoblastic leukaemia (ALL)-associated variants (Fig. 2), and an HSC flanking active TSS (TssAFlnk) chromatin state enrichment for chronic lymphocytic leukaemia (CLL)-associated variants (Additional File 1: Figure S12). A list of the haematopoietic stem cell-specific enhancers driving enrichment for MPN variants, along with context on which variants overlap and what other tissues and cell types these enhancer regions are present in is provided in Additional File 12: Table S11. The complementary list for DNase I hotspots across different tissues and variants is provided in Additional File 13: Table S12. This example analysis (< 1 min runtime) demonstrates the ability for FORGE2 to detect cell type-specific signals for a given GWAS in a fast and trait-agnostic manner.

## Discussion

By applying FORGE2 to thousands of GWAS, we demonstrate the potential of FORGE2 to uncover tissue- and cell type-specific signal and shed light on candidate functional mechanisms underlying GWAS loci. Of the 3604 GWAS evaluated, we identified at least one tissue- or cell-specific association for 2057 GWAS, detecting more associations than FORGE, which can only detect 413 associations for the same data, and highlighting the importance of tissue-specific regulatory elements. Using FORGE2, we profiled associated regulatory element classes such as promoters and enhancers, both for study-level results and for specific variants at the cell type and tissue-specific level. This multidimensional analysis provides a valuable resource for scientists conducting follow up research on GWAS findings.

Among the findings in this study, we discovered tissue-specific enrichments for repressive histone marks and chromatin states, such as H3K27me3, H3K9me3, polycomb-repressed, and heterochromatin chromatin states. This finding is particularly remarkable, as it indicates a link between tissue-specific repressed genomic regions and disease-associated GWAS variants, a finding previously not reported in epigenomic analyses of GWAS data.

In addition, it was thought that previously reported FORGE accessible chromatin results for different phenotypes might be driven by variants located in different CRE classes (e.g. promoters and enhancers, among other CRE classes marked by accessible chromatin). Our analysis shows that previously known open chromatin enrichments can be further refined and separated into associations with promoter-, enhancer-, and transcription-associated histone marks (H3K4me3, H3K4me1, and H3K36me3) and chromatin states (TssA, Enh, and Tx). This provides further knowledge of the underlying classes of regulatory elements involved, yielding insights into potential mechanisms underlying GWAS disease-associated variants.

The observation that GWAS-associated variants for certain phenotypes, such as cognitive performance or electrocardiographic traits, are predominantly enriched for tissue-specific enhancer-associated marks, but not for marks associated with other CREs is noteworthy. Considering the evolution of the phenotype in question might be important to understand the mechanistic causes underlying these findings. Indeed, research on the evolution of complex traits suggests that selective pressure can work to favour or discriminate against regulatory elements linked to specific pathways, which then may become over- or underrepresented for certain phenotypes [18]. In addition, we observe that certain groups of related phenotypes, such as eosinophil percentage of white blood cells, eosinophil percentage of granulocytes, and neutrophil percentage of granulocytes, all co-localise to immune H3K36me3 broadPeak regions, suggesting a potential mechanistic link between multiple associated phenotypes and transcription-associated regions, further hinting to shared mechanisms underlying certain groups of phenotypes.

Evidence relating GWAS findings to underlying disease mechanisms has led to a surge in drug company investment in GWAS-related research, with the aim of identifying more reliable drug targets [19, 20]. However, one of the biggest challenges in translating GWAS findings to the field of drug targets is the pervasive presence of variants with small effects on phenotype (supported by growing evidence [21, 22] and predicted by Fisher’s infinitesimal model [23], also discussed in the recently proposed omnigenic model [24]). So many SNPs may be involved in the genetics of certain polygenic disorders that for schizophrenia, for example, over 70% of 1 Mb blocks in the genome may contribute to disease risk [25]. Therefore, even though some of the genes harbouring small effect variants represent valid drug targets [26, 27], the vast number of potential loci makes the prospect of finding a good drug target for a given disease daunting. These results suggest that we need to discover new methods to narrow our search and inform drug target selection. Our observation that certain groups of phenotypes seem to consistently target specific classes of regulatory regions, plausibly at a systems level, can offer insights into identifying potential higher-level regulators or pathways that may prove easier drug targets.

We also uncovered novel associations between certain phenotypes and DNase I hotspots for particular tissues and cell types, such as the association between waist circumference and mesenchymal stem cells, or between hair colour and melanocytes (quantifying and confirming previously reported high overlap [28] with a enrichment detected by our model). These findings extend current knowledge on the tissue-specificity of GWAS regions and hint to underlying tissue-specific mechanisms for these phenotypes, in addition to pinpointing the regulatory elements and variants underlying these associations for further study.

One important step forward in FORGE2 is the detection of cell type-specific enrichments (as opposed to tissue-specific enrichments). Since FORGE provided a tissue-level multiple testing correction method, it could only detect tissue-specific enrichments. Using FORGE2 H3K4me1 analysis, we not only categorise enrichments at a tissue-specific level, but confirm that specificity analysis can also be performed in a systematic way at the cell type level across several immune cell type categories, including CD3+ T cells, CD19+ B cells, CD14+ monocytes and CD34+ haematopoietic stem cells. This refinement of enrichment accuracy to the level of cell type-specificity is an important step forward.

We also evaluated the relationship between DNase I hotspot, histone mark broadPeak and HMM chromatin state datasets in the context of GWAS analysis, charting shared and specific GWAS phenotypes across these epigenomic marks. Our results show high variability in enrichment across histone marks and chromatin states for GWAS results, with a high number of phenotypes displaying enrichment for certain features, such as H3K4me1, and a low number of phenotypes having enrichment for other features, such as the quiescent (Quies) chromatin state. We also discovered that for some phenotypes, tissue-specific enrichments for marks indicating general regulatory activity (e.g. accessible chromatin) were also present in the same tissue for two or more different marks indicating specific regulatory activity (e.g. active promoters and enhancer chromatin states), with different sets of SNPs underlying each one of these two categories. By understanding which regulatory elements were underlying the previous “accessible chromatin” enrichment and separating these enrichments into different classes of regulatory elements, we can gain a greater understanding of the mechanisms underlying the GWAS associations. We also observed that some phenotypes tend to be associated with specific regulatory regions (e.g. enhancers), and others seem to be associated with multiple types of regulatory marks. Although we may not understand why GWAS SNPs tend to localise to specific types of regulatory regions for some phenotypes, these results are a step forward and provide additional information relating to the underlying biological architecture of genetic susceptibility to these diseases and traits.

Regarding limitations and future analyses, it should be noted that analysis of tissue-specific regulatory regions cannot capture all potential biological mechanisms, but instead provide a tool by which many loci may be understood. In-depth analysis of tissue-invariant GWAS-associated regions, which are also catalogued by FORGE2 and annotated in our results (which cover the full spectrum of regulatory regions across tissues and cell types), may also help understand the biology behind some GWAS associations, but this is outside the scope of this manuscript. In addition, we noted differences in the number of enriched phenotypes between related analysis categories, for example, histone marks characteristic of enhancers, and enhancer chromatin states. This is likely driven in part by the distance between causal variants and GWAS-mapped variants. Broad epigenomic peaks may identify signals precisely because, due to their larger size, they overlap both causal variants and tagged variants which are in linkage disequilibrium. However, finer genomic segmentations, such as HMM chromatin states, which are smaller, cannot do this nearly as well. Advances in fine mapping of GWAS regions may improve our ability to further refine data to help identify functional variants across different phenotypes.

FORGE2 has a specific and unique role in the field of GWAS analysis methods. It performs multidimensional epigenomic data analysis across hundreds of samples and chromatin data types (including DNase I hotspots, histone mark broadPeaks and HMM chromatin states), utilising background SNP sets comprising thousands of selected variants adjusted for multiple genomic biases to robustly test for enrichment, and optimised in a fast and easy to use web tool. Unlike LD score regression [29, 30] FORGE2 does not rely on a specific reference population or require a large GWAS sample size, and it can be used to evaluate results from a broad range of GWAS, including GWAS from multi-ancestry, admixed or unique populations, as well as GWAS on both common and rare diseases. FORGE2 is focused on the statistical analysis of SNP sets, as well as annotating individual variants. FORGE2 analysis of regulatory associations can aid further investigation of variants via laboratory methods (e.g. massively parallel reporter assays/CRISPR experimentation). As such, it possesses unique characteristics and targets a niche different from that of other current tools [10, 29–31].

In the context of computational epigenomic methods for integrative analysis of GWAS data, the utility of a common and easily-accessible framework is noteworthy. While several command-line methods have been developed in this area [31, 32] (reviewed in [33]), issues related to installation across multiple systems, downloading massive epigenomic datasets and keeping local versions up to date with ever-changing computational pipelines have been a challenge for many researchers in the field. An important feature of our approach is the web-based integration of multiple tissue-specific regulatory element datasets to better understand the downstream effects of GWAS findings.

## Conclusions

In conclusion, by providing an easy to use, updated and robust framework for the epigenomic analysis of GWAS data, we have opened the door for experimental and computational researchers to rapidly expand and further characterise GWAS disease-associated regions and mechanisms. We have also provided extensive analyses of the GWAS catalogue as a resource for the community. This computational framework will increase our understanding of the biological underpinnings of complex disease and catalyse the prioritisation of candidate drug targets and pathways, thus accelerating the work of researchers reaping the benefits from the rapidly growing catalogue of GWAS data

## Methods

### Epigenomic data

FORGE2 utilises epigenomic data files that were downloaded from several sites: (1) ENCODE DNase I hotspot files (125 biosamples) from ftp://ftp.ebi.ac.uk/pub/databases/ensembl/encode/integration_data_jan2011/byDataType/openchrom/jan2011/combined_hotspots/ [34]; (2) BLUEPRINT DNase I hotspot files (30 biosamples) from https://blueprint.genomatix.de/grid/experiments/browse (samples listed at European Genome-phenome Archive accession number EGAD00001002713) [7, 35, 36]; (3) Roadmap Epigenomics 2012 DNase I sequencing tag alignment files (299 biosamples) from http://www.genboree.org/EdaccData/Current-Release/experiment-sample/Chromatin_Accessibility/ (GEO accession number GSE18927), and processed these files using the Hotspot method with default settings (http://www.uwencode.org/proj/hotspot/) [11, 37]; (4) Consolidated Roadmap Epigenomics 2015 DNase I hotspot files (39 biosamples) from http://egg2.wustl.edu/roadmap/data/byFileType/peaks/consolidated/broadPeak/; (5) BroadPeak Histone mark files (39 biosamples) from http://egg2.wustl.edu/roadmap/data/byFileType/peaks/consolidated/broadPeak/; and (6) HMM Chromatin State files (39 biosamples) from https://egg2.wustl.edu/roadmap/data/byFileType/chromhmmSegmentations/ChmmModels/coreMarks/jointModel/final/ [6]. Cell and tissue information for each one of these files was obtained using data from the same consortium webpage or decodings available via tables from the ENCODE Data Coordination Centre (https://genome.ucsc.edu/encode/cellTypes.html) or information from sample group SAMEG31306 from the BioSamples database (http://www.ebi.ac.uk/biosamples/).

### FORGE2 database generation

Bedops [38] was used to overlap epigenomic track bed files with SNP positions, where an overlap indicates identical coordinate position for the SNP and epigenomic track at a given base pair. An indexed sqlite database was used to store the overlaps (forge2.db). Data were saved as a binary string, where 1 indicated presence of an overlap between a SNP and an epigenomic track and 0 indicated absence of overlap. Storing results in the sqlite database allows for rapid retrieval of information and thus improves FORGE2 analysis speed.

The addition of new epigenomic track data to the standalone FORGE2 database can be performed using bedtools/bedops and similar openly available code to previous methods [35].

### Functional overlap analysis

FORGE2 takes a set of input SNPs (e.g. a list of rsIDs) and computes their overlap (based on base pair position) with epigenomic track data. For a SNP to be analysed, it must be present in the 1000 genomes dataset (phase I of the 1000 Genomes Project, ftp://ftp.1000genomes.ebi.ac.uk/vol1/ftp/phase1/analysis_results/integrated_call_sets/). To avoid including multiple SNPs that are in linkage disequilibrium (LD), a filter may be applied. The LD filter included in the FORGE2 web tool is based on an international sample of 14 populations from the 1000 Genomes Project (with a default of *r*^2^ ≥ 0.8, population details at https://www.internationalgenome.org/data-portal/data-collection/phase-1). When used, it retains the first SNP from the SNP list and excludes all other SNPs that are in LD with this SNP. The user may also apply their own population-specific LD filter to the input SNP list prior to FORGE2 analysis or utilise other filtering metrics.

To test for enrichment (higher overlap than expected), FORGE2 first computes an overlap score for a SNP set (the sum of all overlaps of that set) against epigenomic track data across a range of cell types and tissues. For each epigenomic track sample, the overlap score of the input set is then compared to the overlap scores of 1000 background SNP sets that FORGE2 generates. SNPs from these 1000 background sets are selected for similarity to the input SNP set, via matching decile bins for minor allele frequency (MAF), distance to transcription start site (TSS) and GC content. A binomial test is used to compare the overlap score of the input SNP set with the overlap scores of the 1000 background sets. This is repeated across multiple cell/tissue samples, until all samples have been analysed. The *p* values from the binomial tests for all cell/tissue samples tested are then adjusted for multiple testing via the Benjamini-Hochberg (BH) method [39], generating *q* values as a result. BH is applied at a sample level, including all samples and *p* values. Enrichments at a *q* value< 0.01 are considered significant. Alongside *q* values, corresponding uncorrected *p* values and *Z*-scores are also provided by FORGE2.

### False positive rate testing

To evaluate the false positive rate, we ran > 27 million random input SNP tests with FORGE2, including 1000 sets of 5, 10, 15, 20, 30, 40, 50, and 100 SNPs. This analysis showed a low level of false positives (7.6 for every 100,000 tests at a BH-corrected *p* value < 0.01). False Discovery Rate (FDR) correction was implemented in a similar way to the eFORGE tool, which previously implemented FDR for the detection of cell type-specific and tissue-specific enrichment signal in epigenome-wide association studies (EWAS) [35, 36, 40].

### Outputs

FORGE2 returns several different outputs, including charts, tables, original raw text files and R code for plotting. Tables and charts are also generated in interactive format to allow for an intuitive exploration of cell type- and tissue-specific enrichment results. Specifically, FORGE2 outputs: a web-based interactive table, a web-based dimple (http://dimplejs.org) d3 interactive graphic using rCharts, a tab-separated values (TSV) file, a PDF file, R code source files for generating the charts and the table, as well as a compressed file with the input data. FORGE2 provides output similar to FORGE [10], eFORGE v1.0, and eFORGE v2.0 [35, 36, 40], and additional information can be found in those references as well as in a recently published EWAS where eFORGE analysis was applied [41].

### GWAS catalogue analysis

We downloaded the NHGRI-EBI GWAS catalogue (2-September-2019 version, all associations v1.0, tab separated file format) from https://www.ebi.ac.uk/gwas /docs/file-downloads. This file contained all genome-wide significant SNPs and traits as determined by GWAS catalogue criteria. We computationally separated the SNPs into distinct files by phenotype (GWAS phenotypes). For this analysis, we required each phenotype (or GWAS) to have a minimum of 5 SNPs. To account for SNP density, we used the default LD filter of *r*^2^ ≥ 0.8 for this analysis. We ran FORGE2 with default settings (i.e. 1000 background repetitions) and a significance threshold of *q* value< 0.01, across the following datasets: DNase I hotspot data from ENCODE, BLUEPRINT, and consolidated and unconsolidated Roadmap Epigenomics, 5 histone mark categories from consolidated Roadmap Epigenomics data and 15 HMM chromatin state datasets from consolidated Roadmap Epigenomics data.

### Clustering analysis and related figures

The clustering analysis was performed using R statistical software. Clustered heatmaps were generated using the pheatmap R package (with default settings including complete linkage clustering, Euclidean distance)[42]. Upset plots were generated using the UpSetR package [15], and other figures were generated via base R plotting. Zoomable versions of all figures were uploaded to easyzoom (https://www.easyzoom.com) and are available in the following easyzoom folder:

https://www.easyzoom.com/albumaccess/3de0ae35c00b41189f63d13635bdb8d9.

## Supplementary Information

**Additional file 1: Supplementary figures S1-S26**. and supplementary figure and table legends.

**Additional file 2: Table S1.** Consolidated Epigenomics Roadmap DNase I hotspot FORGE2 GWAS catalogue analysis results (q-values).

**Additional file 3: Table S2.** Unconsolidated (2012) Epigenomics Roadmap DNase I hotspot FORGE2 GWAS catalogue analysis results (q-values).

**Additional file 4: Table S3.** ENCODE DNase I hotspot FORGE2 GWAS catalogue analysis results (q-values).

**Additional file 5: Table S4.** BLUEPRINT DNase I hotspot FORGE2 GWAS catalogue analysis results (q-values).

**Additional file 6: Table S5.** Consolidated Epigenomics Roadmap H3K4me1 FORGE2 GWAS catalogue analysis results (q-values).

**Additional file 7: Table S6.** Consolidated Epigenomics Roadmap H3K4me3 FORGE2 GWAS catalogue analysis results (q-values).

**Additional file 8: Table S7.** Consolidated Epigenomics Roadmap H3K36me3 FORGE2 GWAS catalogue analysis results (q-values).

**Additional file 9: Table S8.** Consolidated Epigenomics Roadmap H3K27me3 FORGE2 GWAS catalogue analysis results (q-values).

**Additional file 10: Table S9.** Consolidated Epigenomics Roadmap H3K9me3 FORGE2 GWAS catalogue analysis results (q-values).

**Additional file 11: Table S10.** Consolidated Epigenomics Roadmap HMM Chromatin State (15-state model) FORGE2 GWAS catalogue analysis results (q-values).

**Additional file 12: Table S11.** FORGE2 histone mark broadPeak analysis results (q-values, combined H3K4me1, H3K4me3, H3K9me3, H3K36me3, and H3K27me3) for a set of 25 variants from a GWAS on myeloproliferative neoplasms.

**Additional file 13: Table S12.** FORGE2 DNase I hotspot analysis results (q-values) for a set of 25 variants from a GWAS on myeloproliferative neoplasms.

**Additional file 14:** Review history.

## Data Availability

**Data obtention** ENCODE DNase I hotspot files were obtained from (1) ftp://ftp.ebi.ac.uk/pub/databases/ensembl/encode/integration_data_jan2011/byDataType/openchrom/jan2011/combined_hotspots/ [34]; (2) BLUEPRINT DNase I hotspot files were obtained from https://blueprint.genomatix.de/grid/experiments/browse (samples listed at European Genome-phenome Archive accession number EGAD00001002713) [7, 35, 36]; (3) Roadmap Epigenomics 2012 DNase I sequencing tag alignment files were obtained from http://www.genboree.org/EdaccData/Current-Release/experiment-sample/Chromatin_Accessibility/ (GEO accession number GSE18927) [11, 37]; (4) Consolidated Roadmap Epigenomics 2015 DNase I hotspot files were obtained from http://egg2.wustl.edu/roadmap/data/byFileType/peaks/consolidated/broadPeak/; (5) BroadPeak Histone mark files were obtained from http://egg2.wustl.edu/roadmap/data/byFileType/peaks/consolidated/broadPeak/; and (6) HMM Chromatin State files were obtained from https://egg2.wustl.edu/roadmap/data/byFileType/chromhmmSegmentations/ChmmModels/coreMarks/jointModel/final/ [6]. Cell and tissue data for each one of these files were obtained either from the same consortium webpage or the decodings available via tables from the ENCODE Data Coordination Centre (https://genome.ucsc.edu/encode/cellTypes.html) or information from sample group SAMEG31306 from the BioSamples database (http://www.ebi.ac.uk/biosamples/). **Source code and additional files** FORGE2 code has been tested on Mac OSX 10.12.6 and Linux Centos 7. Source code is available at https://github.com/charlesbreeze/FORGE2 under the GNU General Public License Version 3. Source code and additional files (including main figures, supplementary figures, supplementary tables and relevant legends) are available on Zenodo at 10.5281/zenodo.5719754 [43, 44]. Information for generating FORGE2/eFORGE-type databases from scratch can be found here: https://github.com/charlesbreeze/eFORGE/blob/master/database/README.txt. Example code for creating FORGE2 plot subsets can be found on GitHub at: https://github.com/charlesbreeze/FORGE2/blob/forge2.v1.0/bin/plot.subsets/plot.subsets.txt. All input SNP lists, output analyses and figures have been uploaded to the FORGE2 Google Drive directory and are accessible from the following link: https://drive.google.com/drive/folders/10GYaZQj_dF8FVj8LzMMBTlf1MwNBIIpU?usp=sharing.
