# Supplementary material for: Integrative analysis of 3604 GWAS reveals multiple novel cell type-specific regulatory associations
Source: Genome Biol. 2022 Jan 7;23:13. doi: 10.1186/s13059-021-02560-3 (PMC8742386; doi:10.1186/s13059-021-02560-3)
Supplement: Supplementary file 1 — Additional file 1: Supplementary figures S1-S26. and supplementary figure and table legends. [file 13059_2021_2560_MOESM1_ESM.pdf]

Supplementary Figures

Integrative analysis across 3,604 GWAS reveals multiple novel cell type-specific regulatory associations

Charles E. Breeze[1,2,3,9,10], Eric Haugen[2], Alex Reynolds[2], Andrew Teschendorff[4], Jenny van Dongen[5], Qing Lan[1], Nathaniel Rothman[1], Guillaume Bourque[6], Ian Dunham[7], Stephan Beck[3], John Stamatoyannopoulos[2], Nora Franceschini[8], and Sonja I. Berndt[1].

[1] National Cancer Institute, NIH, Bethesda, MD 20892

[2] Altius Institute for Biomedical Sciences, Seattle, WA 98121

[3] UCL Cancer Institute, University College London, London, WC1E 6BT, UK

[4] CAS Key Laboratory of Computational Biology, CAS-MPG Partner Institute for Computational Biology, Shanghai Institute of Nutrition and Health, Shanghai Institute for Biological Sciences, University of Chinese Academy of Sciences, Chinese Academy of Sciences, 320 Yue Yang Road, Shanghai, 200031, China

[5] Department of Biological Psychology, Vrije Universiteit Amsterdam, Amsterdam 1081BT, The Netherlands

[6] Department of Human Genetics, McGill University and Génome Québec Innovation Center, Montréal H3A 0G1, Canada

[7] European Molecular Biology Laboratory, European Bioinformatics Institute (EMBL-EBI), Wellcome Genome Campus, Hinxton, Cambridge, CB10 1SD, UK

[8] Department of Epidemiology, Gillings School of Global Public Health, University of North Carolina, Chapel Hill, North Carolina, USA

[9] Corresponding author: c.breeze@ucl.ac.uk

Supplementary figure legends

Zoomable versions of figures S1-S26 can be found at: https://www.easyzoom.com/albumaccess/94ac1fa38e944c66b6470fea0c35cb18

Figure S1: FORGE2 analysis: [A] FORGE2 takes multidimensional data across 15 chromatin states, 5 histone marks, and DNase I hypersensitive sites across hundreds of samples and overlays them with GWAS SNPs. [B] FORGE2 then compares overlap results to those obtained using background SNPs (correcting for distance to TSS, MAF and GC content) [C]. This comparison results in an enrichment score across many cell types and tissues, which can identify cell types or tissues where the GWAS SNPs present a high enrichment score.

Figure S2: GWAS enrichments for DNase I hotspots from sample-specific or unconsolidated Roadmap Epigenomics consortium data: Shown are significant tissue specific enrichment results (q-value <0.01, Benjamini-Hochberg -BH- correction) for FORGE2 analysis across the NHGRI/EBI GWAS catalogue (downloaded 2-September-2019, row-normalised values). GWAS phenotypes (columns) are clustered using complete linkage clustering (Euclidean distance), while different tissues and cell types (rows) are grouped according to related tissue or cell type categories (e.g. white blood cell categories are placed together, as are fibroblast categories, etc.). Clustering reveals groups of related phenotypes with similar tissue-specific enrichment profiles, such as immune traits for blood or cardiac traits for heart tissue.

Figure S3: GWAS enrichments for DNase I hotspots from the ENCODE consortium: Shown are significant tissue specific enrichment results (q-value <0.01, Benjamini-Hochberg -BH- correction) for FORGE2 analysis across the NHGRI/EBI GWAS catalogue (downloaded 2-September-2019, row-normalised values). GWAS phenotypes (columns) are clustered using complete linkage clustering (Euclidean distance), while different tissues and cell types (rows) are grouped according to related tissue or cell type categories (e.g. white blood cell categories are placed together, as are fibroblast categories, etc.). Clustering reveals groups of related phenotypes with similar tissue-specific enrichment profiles.

Figure S4: GWAS enrichments for DNase I hotspots from the BLUEPRINT consortium: Shown are significant tissue specific enrichment results (q-value <0.01, Benjamini-Hochberg -BH- correction) for FORGE2 analysis across the NHGRI/EBI GWAS catalogue (downloaded 2-September-2019, row-normalised values). GWAS phenotypes (columns) are clustered using complete linkage clustering (Euclidean distance), while different tissues and cell types (rows) are grouped according to related tissue or cell type categories (e.g. white blood cell categories are placed together, as are fibroblast categories, etc.). Clustering reveals groups of related phenotypes with similar tissue-specific enrichment profiles.

Figure S5: GWAS enrichments for H3K4me1 broadPeaks from consolidated Roadmap Epigenomics consortium data: Shown are significant tissue specific enrichment results (q-value <0.01, Benjamini-Hochberg -BH- correction) for FORGE2 analysis across the NHGRI/EBI GWAS catalogue (downloaded 2-September-2019, row-normalised values). GWAS phenotypes (columns) are clustered using complete linkage clustering (Euclidean distance), while different tissues and cell types (rows) are grouped according to related tissue or cell type categories (e.g. white blood cell categories are placed together, as are fibroblast categories, etc.). Clustering reveals groups of related phenotypes with similar tissue-specific enrichment profiles.

Figure S6: GWAS enrichments for H3K4me3 broadPeaks from consolidated Roadmap Epigenomics consortium data: Shown are significant tissue specific enrichment results (q-value <0.01, Benjamini-Hochberg -BH- correction) for FORGE2 analysis across the NHGRI/EBI GWAS catalogue (downloaded 2-September-2019, row-normalised values). GWAS phenotypes (columns) are clustered using complete linkage clustering (Euclidean distance), while different tissues and cell types (rows) are grouped according to related tissue or cell type categories (e.g. white blood cell categories are placed together, as are fibroblast categories, etc.). Clustering reveals groups of related phenotypes with similar tissue-specific enrichment profiles.

Figure S7: GWAS enrichments for H3K36me3 broadPeaks from consolidated Roadmap Epigenomics consortium data: Shown are significant tissue specific enrichment results (q-value <0.01, Benjamini-Hochberg -BH- correction) for FORGE2 analysis across the NHGRI/EBI GWAS catalogue (downloaded 2-September-2019, row-normalised values). GWAS phenotypes (columns) are clustered using complete linkage clustering (Euclidean distance), while different tissues and cell types (rows) are grouped according to related tissue or cell type categories (e.g. white blood cell categories are placed together, as are fibroblast categories, etc.). Clustering reveals groups of related phenotypes with similar tissue-specific enrichment profiles.

Figure S8: GWAS enrichments for H3K27me3 broadPeaks from consolidated Roadmap Epigenomics consortium data: Shown are significant tissue specific enrichment results (q-value <0.01, Benjamini-Hochberg -BH- correction) for FORGE2 analysis across the NHGRI/EBI GWAS catalogue (downloaded 2-September-2019, row-normalised values). GWAS phenotypes (columns) are clustered using complete linkage clustering (Euclidean distance), while different tissues and cell types (rows) are grouped according to related tissue or cell type categories (e.g. white blood cell categories are placed together, as are fibroblast categories, etc.). Clustering reveals groups of related phenotypes with similar tissue-specific enrichment profiles.

Figure S9: GWAS enrichments for H3K9me3 broadPeaks from consolidated Roadmap Epigenomics consortium data: Shown are significant tissue specific enrichment results (q-value <0.01, Benjamini-Hochberg -BH- correction) for FORGE2 analysis across the NHGRI/EBI GWAS catalogue (downloaded 2-September-2019, row-normalised values). GWAS phenotypes (columns) are

clustered using complete linkage clustering (Euclidean distance), while different tissues and cell types (rows) are grouped according to related tissue or cell type categories (e.g. white blood cell categories are placed together, as are fibroblast categories, etc.). Clustering reveals groups of related phenotypes with similar tissue-specific enrichment profiles.

Figure S10: Shared and unique FORGE2 results across different histone marks: Upset plot showing shared and unique (non-shared) GWAS phenotypes that present significant enrichments for histone mark broadPeak datasets from consolidated Roadmap Epigenomics consortium data. Upset plot grouping reveals ranked numbers of phenotypes presenting enrichment profiles across specific histone mark intersections (top barplot), also indicating the precise number of GWAS phenotypes that are present in each intersection (intersection size number indicated at the top of each bar). The left-hand barplot shows the number of total significantly enriched phenotypes for each histone mark, regardless of intersection (set size).

Figure S11: GWAS enrichments for active transcription start site -TSS- (TssA) chromatin state regions from consolidated Roadmap Epigenomics consortium data: Shown are significant tissue specific enrichment results (q-value <0.01, Benjamini-Hochberg -BH- correction) for FORGE2 analysis across the NHGRI/EBI GWAS catalogue (downloaded 2-September-2019, row-normalised values). GWAS phenotypes (columns) are clustered using complete linkage clustering (Euclidean distance), while different tissues and cell types (rows) are grouped according to related tissue or cell type categories (e.g. white blood cell categories are placed together, as are fibroblast categories, etc.). Clustering reveals groups of related phenotypes with similar tissue-specific enrichment profiles.

Figure S12: GWAS enrichments for flanking active TSS (TssAFlnk) chromatin state regions from consolidated Roadmap Epigenomics consortium data: Shown are significant tissue specific enrichment results (q-value <0.01, Benjamini-Hochberg -BH- correction) for FORGE2 analysis across the NHGRI/EBI GWAS catalogue (downloaded 2-September-2019, row-normalised values). GWAS phenotypes (columns) are clustered using complete linkage clustering (Euclidean distance), while different tissues and cell types (rows) are grouped according to related tissue or cell type categories (e.g. white blood cell categories are placed together, as are fibroblast categories, etc.). Clustering reveals groups of related phenotypes with similar tissue-specific enrichment profiles.

Figure S13: GWAS enrichments for bivalent/poised TSS (TssBiv) chromatin state regions from consolidated Roadmap Epigenomics consortium data: Shown are significant tissue specific enrichment results (q-value <0.01, Benjamini-Hochberg -BH- correction) for FORGE2 analysis across the NHGRI/EBI GWAS catalogue (downloaded 2-September-2019, row-normalised values). GWAS phenotypes (columns) are clustered using complete linkage clustering (Euclidean distance), while different tissues and cell types (rows) are grouped according to related tissue or cell type categories (e.g. white blood cell categories are placed

together, as are fibroblast categories, etc.). Clustering reveals groups of related phenotypes with similar tissue-specific enrichment profiles.

Figure S14: GWAS enrichments for strong transcription (Tx) chromatin state regions from consolidated Roadmap Epigenomics consortium data: Shown are significant tissue specific enrichment results (q-value <0.01, Benjamini-Hochberg -BH- correction) for FORGE2 analysis across the NHGRI/EBI GWAS catalogue (downloaded 2-September-2019, row-normalised values). GWAS phenotypes (columns) are clustered using complete linkage clustering (Euclidean distance), while different tissues and cell types (rows) are grouped according to related tissue or cell type categories (e.g. white blood cell categories are placed together, as are fibroblast categories, etc.). Clustering reveals groups of related phenotypes with similar tissue-specific enrichment profiles.

Figure S15: GWAS enrichments for transcription at 5' and 3' ends of a gene (TxFlnk) chromatin state regions from consolidated Roadmap Epigenomics consortium data: Shown are significant tissue specific enrichment results (q-value <0.01, Benjamini-Hochberg -BH- correction) for FORGE2 analysis across the NHGRI/EBI GWAS catalogue (downloaded 2-September-2019, row-normalised values). GWAS phenotypes (columns) are clustered using complete linkage clustering (Euclidean distance), while different tissues and cell types (rows) are grouped according to related tissue or cell type categories (e.g. white blood cell categories are placed together, as are fibroblast categories, etc.). Clustering reveals groups of related phenotypes with similar tissue-specific enrichment profiles.

Figure S16: GWAS enrichments for weak transcription (TxWk) chromatin state regions from consolidated Roadmap Epigenomics consortium data: Shown are significant tissue specific enrichment results (q-value <0.01, Benjamini-Hochberg -BH- correction) for FORGE2 analysis across the NHGRI/EBI GWAS catalogue (downloaded 2-September-2019, row-normalised values). GWAS phenotypes (columns) are clustered using complete linkage clustering (Euclidean distance), while different tissues and cell types (rows) are grouped according to related tissue or cell type categories (e.g. white blood cell categories are placed together, as are fibroblast categories, etc.). Clustering reveals groups of related phenotypes with similar tissue-specific enrichment profiles.

Figure S17: GWAS enrichments for ZNF genes and repeats (ZNF-Rpts) chromatin state regions from consolidated Roadmap Epigenomics consortium data: Shown are significant tissue specific enrichment results (q-value <0.01, Benjamini-Hochberg -BH- correction) for FORGE2 analysis across the NHGRI/EBI GWAS catalogue (downloaded 2-September-2019, row-normalised values). GWAS phenotypes (columns) are clustered using complete linkage clustering (Euclidean distance), while different tissues and cell types (rows) are grouped according to related tissue or cell type categories (e.g. white blood cell categories are placed together, as are fibroblast categories, etc.). Clustering reveals groups of related phenotypes with similar tissue-specific enrichment profiles.

Figure S18: GWAS enrichments for flanking bivalent TSS/enhancer (BivFlnk) chromatin state regions from consolidated Roadmap Epigenomics consortium data: Shown are significant tissue specific enrichment results (q-value <0.01, Benjamini-Hochberg -BH- correction) for FORGE2 analysis across the NHGRI/EBI GWAS catalogue (downloaded 2-September-2019, row-normalised values). GWAS phenotypes (columns) are clustered using complete linkage clustering (Euclidean distance), while different tissues and cell types (rows) are grouped according to related tissue or cell type categories (e.g. white blood cell categories are placed together, as are fibroblast categories, etc.). Clustering reveals groups of related phenotypes with similar tissue-specific enrichment profiles.

Figure S19: GWAS enrichments for enhancer (Enh) chromatin state regions from consolidated Roadmap Epigenomics consortium data: Shown are significant tissue specific enrichment results (q-value <0.01, Benjamini-Hochberg -BH- correction) for FORGE2 analysis across the NHGRI/EBI GWAS catalogue (downloaded 2-September-2019, row-normalised values). GWAS phenotypes (columns) are clustered using complete linkage clustering (Euclidean distance), while different tissues and cell types (rows) are grouped according to related tissue or cell type categories (e.g. white blood cell categories are placed together, as are fibroblast categories, etc.). Clustering reveals groups of related phenotypes with similar tissue-specific enrichment profiles.

Figure S20: GWAS enrichments for bivalent enhancer (EnhBiv) chromatin state regions from consolidated Roadmap Epigenomics consortium data: Shown are significant tissue specific enrichment results (q-value <0.01, Benjamini-Hochberg -BH- correction) for FORGE2 analysis across the NHGRI/EBI GWAS catalogue (downloaded 2-September-2019, row-normalised values). GWAS phenotypes (columns) are clustered using complete linkage clustering (Euclidean distance), while different tissues and cell types (rows) are grouped according to related tissue or cell type categories (e.g. white blood cell categories are placed together, as are fibroblast categories, etc.). Clustering reveals groups of related phenotypes with similar tissue-specific enrichment profiles.

Figure S21: GWAS enrichments for genic enhancer (EnhG) chromatin state regions from consolidated Roadmap Epigenomics consortium data: Shown are significant tissue specific enrichment results (q-value <0.01, Benjamini-Hochberg -BH- correction) for FORGE2 analysis across the NHGRI/EBI GWAS catalogue (downloaded 2-September-2019, row-normalised values). GWAS phenotypes (columns) are clustered using complete linkage clustering (Euclidean distance), while different tissues and cell types (rows) are grouped according to related tissue or cell type categories (e.g. white blood cell categories are placed together, as are fibroblast categories, etc.). Clustering reveals groups of related phenotypes with similar tissue-specific enrichment profiles.

Figure S22: GWAS enrichments for heterochromatin (Het) chromatin state regions from consolidated Roadmap Epigenomics consortium data: Shown are significant tissue specific enrichment results (q-value <0.01, Benjamini-Hochberg -BH- correction) for FORGE2 analysis across the NHGRI/EBI GWAS

catalogue (downloaded 2-September-2019, row-normalised values). GWAS phenotypes (columns) are clustered using complete linkage clustering (Euclidean distance), while different tissues and cell types (rows) are grouped according to related tissue or cell type categories (e.g. white blood cell categories are placed together, as are fibroblast categories, etc.). Clustering reveals groups of related phenotypes with similar tissue-specific enrichment profiles.

Figure S23: GWAS enrichments for quiescent/low (Quies) chromatin state regions from consolidated Roadmap Epigenomics consortium data: Shown are significant tissue specific enrichment results (q-value <0.01, Benjamini-Hochberg -BH- correction) for FORGE2 analysis across the NHGRI/EBI GWAS catalogue (downloaded 2-September-2019, row-normalised values). GWAS phenotypes (columns) are clustered using complete linkage clustering (Euclidean distance), while different tissues and cell types (rows) are grouped according to related tissue or cell type categories (e.g. white blood cell categories are placed together, as are fibroblast categories, etc.). Clustering reveals groups of related phenotypes with similar tissue-specific enrichment profiles.

Figure S24: GWAS enrichments for repressed polycomb (ReprPC) chromatin state regions from consolidated Roadmap Epigenomics consortium data: Shown are significant tissue specific enrichment results (q-value <0.01, Benjamini-Hochberg -BH- correction) for FORGE2 analysis across the NHGRI/EBI GWAS catalogue (downloaded 2-September-2019, row-normalised values). GWAS phenotypes (columns) are clustered using complete linkage clustering (Euclidean distance), while different tissues and cell types (rows) are grouped according to related tissue or cell type categories (e.g. white blood cell categories are placed together, as are fibroblast categories, etc.). Clustering reveals groups of related phenotypes with similar tissue-specific enrichment profiles.

Figure S25: GWAS enrichments for weak repressed polycomb (ReprPCWk) chromatin state regions from consolidated Roadmap Epigenomics consortium data: Shown are significant tissue specific enrichment results (q-value <0.01, Benjamini-Hochberg -BH- correction) for FORGE2 analysis across the NHGRI/EBI GWAS catalogue (downloaded 2-September-2019, row-normalised values). GWAS phenotypes (columns) are clustered using complete linkage clustering (Euclidean distance), while different tissues and cell types (rows) are grouped according to related tissue or cell type categories (e.g. white blood cell categories are placed together, as are fibroblast categories, etc.). Clustering reveals groups of related phenotypes with similar tissue-specific enrichment profiles.

Figure S26: Shared and unique FORGE2 results across different chromatin states: Upset plot showing shared and unique (non-shared) GWAS phenotypes that present significant enrichments for Roadmap Epigenomics chromatin state datasets. Upset plot grouping reveals ranked numbers of phenotypes presenting enrichment profiles across specific chromatin state intersections (top barplot), also indicating the precise number of GWAS phenotypes that are present in each intersection (intersection size number indicated at the top of each bar). The left-

hand barplot shows the number of total significantly enriched phenotypes for each chromatin state, regardless of intersection (set size).

Supplementary table legends

Table S1: Consolidated Epigenomics Roadmap DNase I hotspot FORGE2 GWAS catalog analysis results (q-values): Different tissues and cell types (rows) are grouped according to related tissue or cell type categories (e.g. white blood cell categories are placed together, as are fibroblast categories, etc.). Tables include FORGE2 analysis results which reveal different tissue/cell type-specific enrichment profiles, as indicated by q-values for different tissue/cell type samples (rows) analysed across a range of traits/diseases (columns, Benjamini-Hochberg -BH- correction).

Table S2: Unconsolidated (2012) Epigenomics Roadmap DNase I hotspot FORGE2 GWAS catalog analysis results (q-values): Different tissues and cell types (rows) are grouped according to related tissue or cell type categories (e.g. white blood cell categories are placed together, as are fibroblast categories, etc.). Tables include FORGE2 analysis results which reveal different tissue/cell type-specific enrichment profiles, as indicated by q-values for different tissue/cell type samples (rows) analysed across a range of traits/diseases (columns, Benjamini-Hochberg -BH- correction).

Table S3: ENCODE DNase I hotspot FORGE2 GWAS catalog analysis results (q-values): Different tissues and cell types (rows) are grouped according to related tissue or cell type categories (e.g. white blood cell categories are placed together, as are fibroblast categories, etc.). Tables include FORGE2 analysis results which reveal different tissue/cell type-specific enrichment profiles, as indicated by q-values for different tissue/cell type samples (rows) analysed across a range of traits/diseases (columns, Benjamini-Hochberg -BH- correction).

Table S4: BLUEPRINT DNase I hotspot FORGE2 GWAS catalog analysis results (q-values): Different tissues and cell types (rows) are grouped according to related tissue or cell type categories (e.g. white blood cell categories are placed together, as are fibroblast categories, etc.). Tables include FORGE2 analysis results which reveal different tissue/cell type-specific enrichment profiles, as indicated by q-values for different tissue/cell type samples (rows) analysed across a range of traits/diseases (columns, Benjamini-Hochberg -BH- correction).

Table S5: Consolidated Epigenomics Roadmap H3K4me1 FORGE2 GWAS catalog analysis results (q-values): Different tissues and cell types (rows) are grouped according to related tissue or cell type categories (e.g. white blood cell categories are placed together, as are fibroblast categories, etc.). Tables include FORGE2 analysis results which reveal different tissue/cell type-specific enrichment profiles, as indicated by q-values for different tissue/cell type samples (rows) analysed across a range of traits/diseases (columns, Benjamini-Hochberg -BH- correction).

Table S6: Consolidated Epigenomics Roadmap H3K4me3 FORGE2 GWAS catalog analysis results (q-values): Different tissues and cell types (rows) are grouped according to related tissue or cell type categories (e.g. white blood cell categories are placed together, as are fibroblast categories, etc.). Tables include FORGE2 analysis results which reveal different tissue/cell type-specific enrichment profiles, as indicated by q-values for different tissue/cell type samples (rows) analysed across a range of traits/diseases (columns, Benjamini-Hochberg -BH- correction).

Table S7: Consolidated Epigenomics Roadmap H3K36me3 FORGE2 GWAS catalog analysis results (q-values): Different tissues and cell types (rows) are grouped according to related tissue or cell type categories (e.g. white blood cell categories are placed together, as are fibroblast categories, etc.). Tables include FORGE2 analysis results which reveal different tissue/cell type-specific enrichment profiles, as indicated by q-values for different tissue/cell type samples (rows) analysed across a range of traits/diseases (columns, Benjamini-Hochberg -BH- correction).

Table S8: Consolidated Epigenomics Roadmap H3K27me3 FORGE2 GWAS catalog analysis results (q-values): Different tissues and cell types (rows) are grouped according to related tissue or cell type categories (e.g. white blood cell categories are placed together, as are fibroblast categories, etc.). Tables include FORGE2 analysis results which reveal different tissue/cell type-specific enrichment profiles, as indicated by q-values for different tissue/cell type samples (rows) analysed across a range of traits/diseases (columns, Benjamini-Hochberg -BH- correction).

Table S9: Consolidated Epigenomics Roadmap H3K9me3 FORGE2 GWAS catalog analysis results (q-values): Different tissues and cell types (rows) are grouped according to related tissue or cell type categories (e.g. white blood cell categories are placed together, as are fibroblast categories, etc.). Tables include FORGE2 analysis results which reveal different tissue/cell type-specific enrichment profiles, as indicated by q-values for different tissue/cell type samples (rows) analysed across a range of traits/diseases (columns, Benjamini-Hochberg -BH- correction).

Table S10: Consolidated Epigenomics Roadmap HMM Chromatin State (15-state model) FORGE2 GWAS catalog analysis results (q-values): Different tissues and cell types (rows) are grouped according to related tissue or cell type categories (e.g. white blood cell categories are placed together, as are fibroblast categories, etc.). Tables include FORGE2 analysis results which reveal different tissue/cell type-specific enrichment profiles, as indicated by q-values for different tissue/cell type samples (rows) analysed across a range of traits/diseases (columns, Benjamini-Hochberg -BH- correction).

Table S11: FORGE2 histone mark broadPeak analysis results (q-values, combined H3K4me1, H3K4me3, H3K9me3, H3K36me3, and H3K27me3) for a set of 25 variants from a GWAS on myeloproliferative neoplasms: Different tissues and cell types (rows) are grouped according to related tissue or cell type

categories (e.g. white blood cell categories are placed together, as are fibroblast categories, etc.). Significant (q-value <0.01, Benjamini-Hochberg -BH- correction) FORGE2 analysis results reveal a tissue-specific enrichment profile for CD34+ haematopoietic stem cell H3K4me1 broadPeaks, a mark enriched for enhancer regions.

Table S12: FORGE2 DNase I hotspot analysis results (q-values) for a set of 25 variants from a GWAS on myeloproliferative neoplasms: Different tissues and cell types (rows) are grouped according to related tissue or cell type categories (e.g. white blood cell categories are placed together, as are fibroblast categories, etc.). Significant (q-value <0.01, Benjamini-Hochberg -BH- correction) FORGE2 analysis results reveal a tissue-specific enrichment profile for CD34+ haematopoietic stem cell DNase I hotspots, a mark representative of all classes of known cis-regulatory elements.

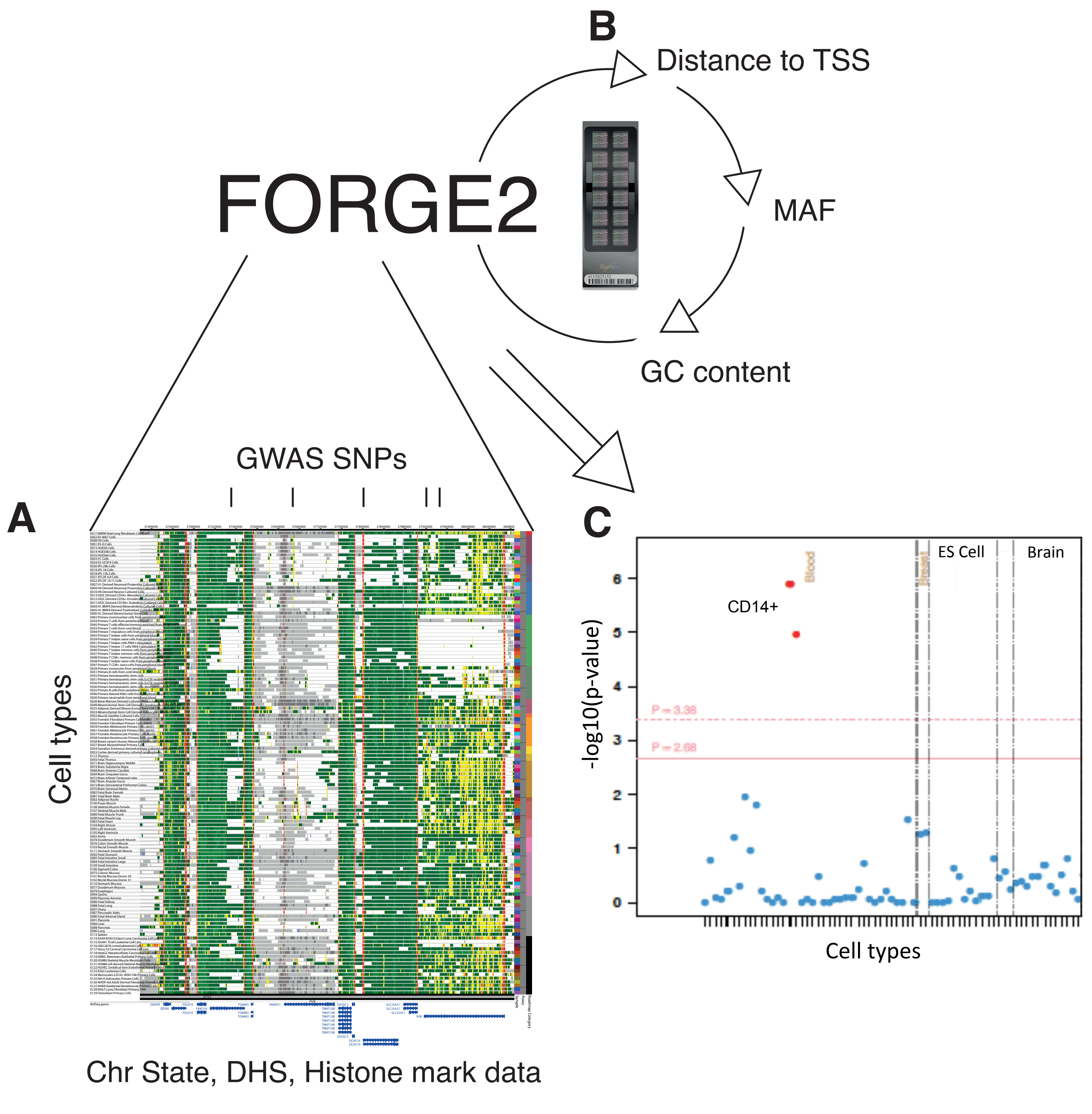
B
Distance to TSS
MAF
GC content
FORGE2
GWAS SNPs
A
Cell types
Chr State, DHS, Histone mark data
C
CD14+
Blood
ES Cell
Brain
-log10(p-value)
Cell types

Figure S1

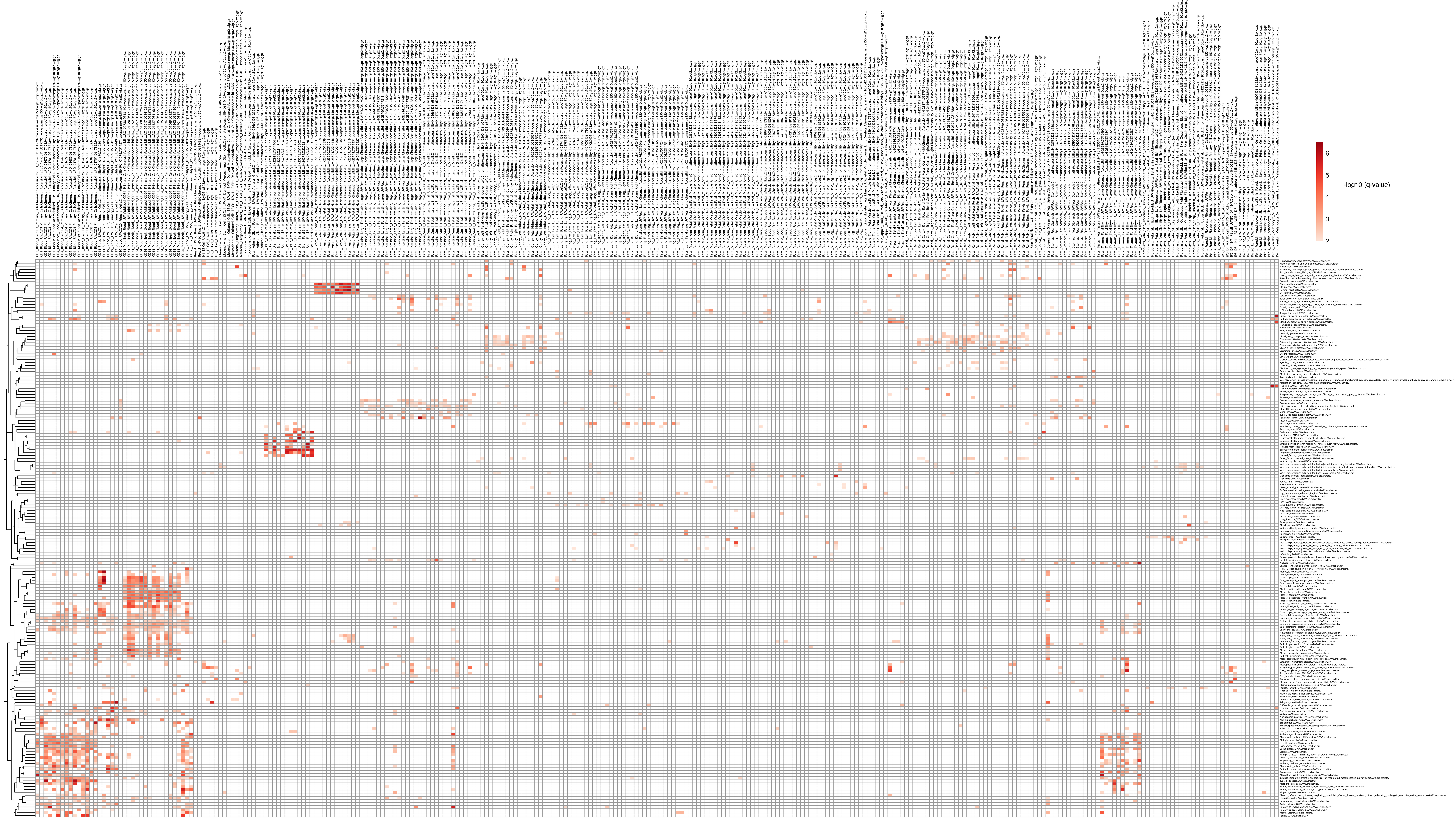

Figure S2

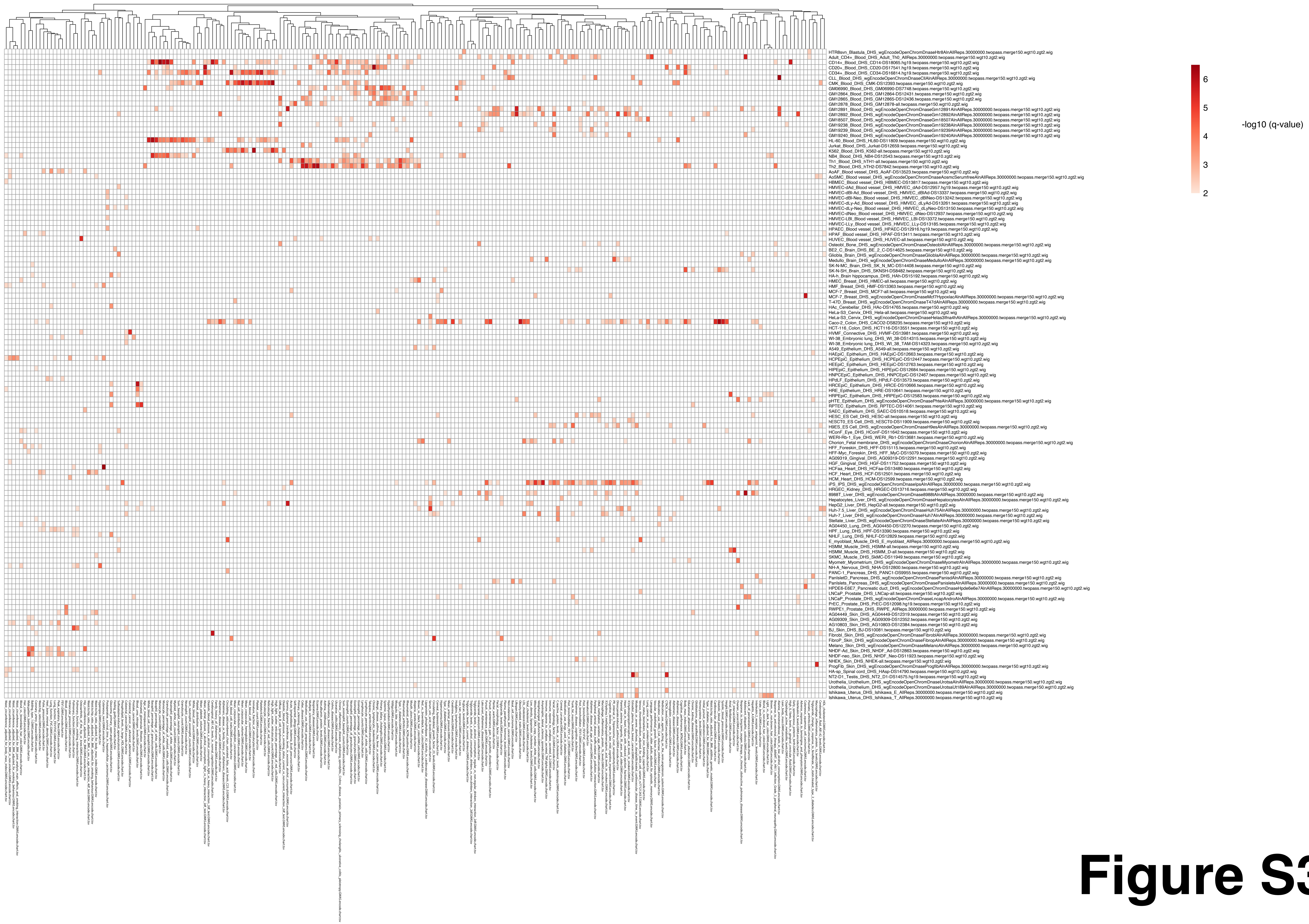

Figure S3

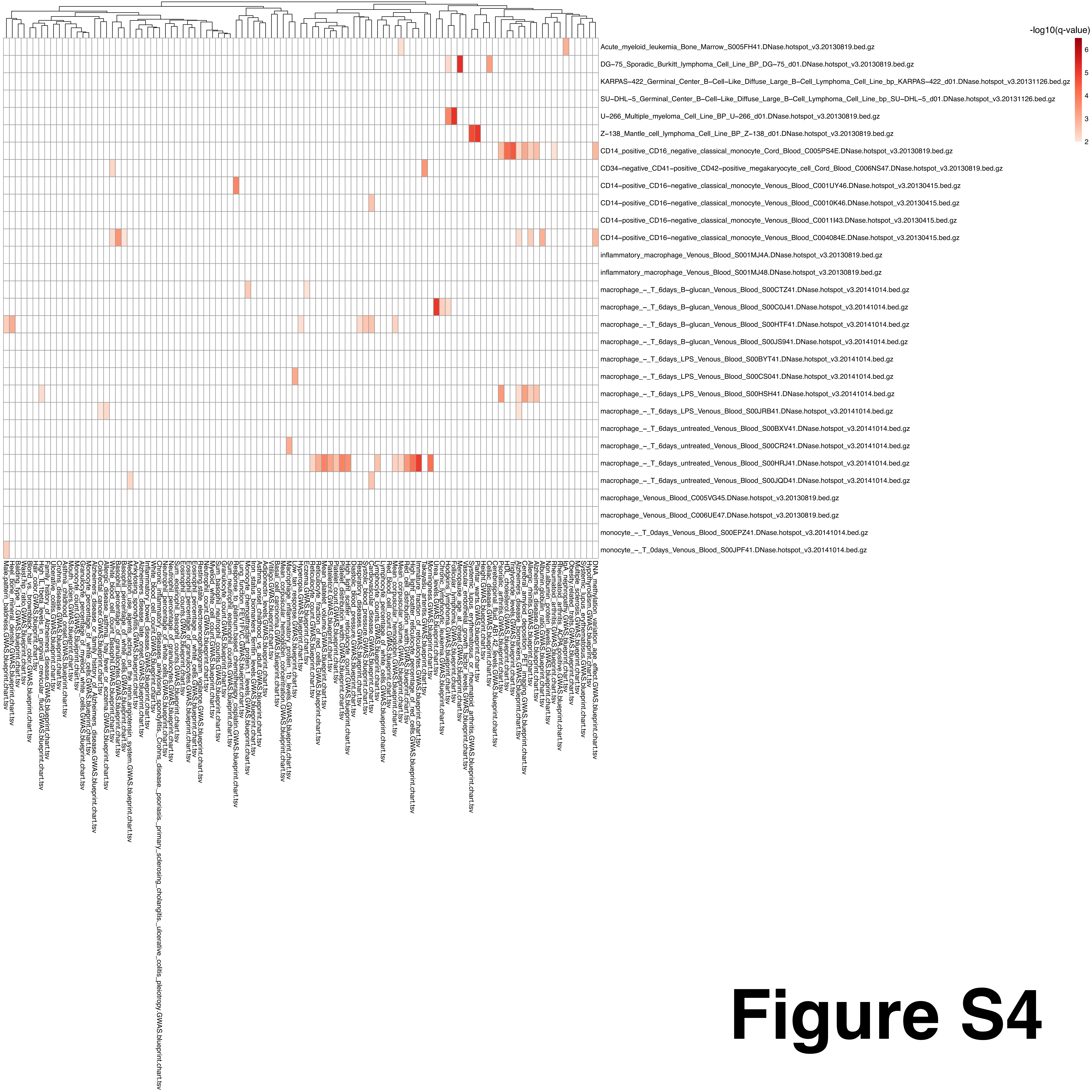
-log10(q-value)
6
5
4
3
2
Acute_myeloid_leukemia_Bone_Marrow_S005FH41.DNase.hotspot_v3.20130819.bed.gz
DG−75_Sporadic_Burkitt_lymphoma_Cell_Line_BP_DG−75_d01.DNase.hotspot_v3.20130819.bed.gz
KARPAS−422_Germinal_Center_B−Cell−Like_Diffuse_Large_B−Cell_Lymphoma_Cell_Line_bp_KARPAS−422_d01.DNase.hotspot_v3.20131126.bed.gz
SU−DHL−5_Germinal_Center_B−Cell−Like_Diffuse_Large_B−Cell_Lymphoma_Cell_Line_bp_SU−DHL−5_d01.DNase.hotspot_v3.20131126.bed.gz
U−266_Multiple_myeloma_Cell_Line_BP_U−266_d01.DNase.hotspot_v3.20130819.bed.gz
Z−138_Mantle_cell_lymphoma_Cell_Line_BP_Z−138_d01.DNase.hotspot_v3.20130819.bed.gz
CD14_positive_CD16_negative_classical_monocyte_Cord_Blood_C005PS4E.DNase.hotspot_v3.20130819.bed.gz
CD34−negative_CD41−positive_CD42−positive_megakaryocyte_cell_Cord_Blood_C006NS47.DNase.hotspot_v3.20130819.bed.gz
CD14−positive_CD16−negative_classical_monocyte_Venous_Blood_C001UY46.DNase.hotspot_v3.20130415.bed.gz
CD14−positive_CD16−negative_classical_monocyte_Venous_Blood_C0010K46.DNase.hotspot_v3.20130415.bed.gz
CD14−positive_CD16−negative_classical_monocyte_Venous_Blood_C0011I43.DNase.hotspot_v3.20130415.bed.gz
CD14−positive_CD16−negative_classical_monocyte_Venous_Blood_C004084E.DNase.hotspot_v3.20130415.bed.gz
inflammatory_macrophage_Venous_Blood_S001MJ4A.DNase.hotspot_v3.20130819.bed.gz
inflammatory_macrophage_Venous_Blood_S001MJ48.DNase.hotspot_v3.20130819.bed.gz
macrophage_−_T_6days_B−glucan_Venous_Blood_S00CTZ41.DNase.hotspot_v3.20141014.bed.gz
macrophage_−_T_6days_B−glucan_Venous_Blood_S00C0J41.DNase.hotspot_v3.20141014.bed.gz
macrophage_−_T_6days_B−glucan_Venous_Blood_S00HTF41.DNase.hotspot_v3.20141014.bed.gz
macrophage_−_T_6days_B−glucan_Venous_Blood_S00JS941.DNase.hotspot_v3.20141014.bed.gz
macrophage_−_T_6days_LPS_Venous_Blood_S00BYT41.DNase.hotspot_v3.20141014.bed.gz
macrophage_−_T_6days_LPS_Venous_Blood_S00CS041.DNase.hotspot_v3.20141014.bed.gz
macrophage_−_T_6days_LPS_Venous_Blood_S00HSH41.DNase.hotspot_v3.20141014.bed.gz
macrophage_−_T_6days_LPS_Venous_Blood_S00JRB41.DNase.hotspot_v3.20141014.bed.gz
macrophage_−_T_6days_untreated_Venous_Blood_S00BXV41.DNase.hotspot_v3.20141014.bed.gz
macrophage_−_T_6days_untreated_Venous_Blood_S00CR241.DNase.hotspot_v3.20141014.bed.gz
macrophage_−_T_6days_untreated_Venous_Blood_S00HRJ41.DNase.hotspot_v3.20141014.bed.gz
macrophage_−_T_6days_untreated_Venous_Blood_S00JQD41.DNase.hotspot_v3.20141014.bed.gz
macrophage_Venous_Blood_C005VG45.DNase.hotspot_v3.20130819.bed.gz
macrophage_Venous_Blood_C006UE47.DNase.hotspot_v3.20130819.bed.gz
monocyte_−_T_0days_Venous_Blood_S00EPZ41.DNase.hotspot_v3.20141014.bed.gz
monocyte_−_T_0days_Venous_Blood_S00JPF41.DNase.hotspot_v3.20141014.bed.gz

Figure S4

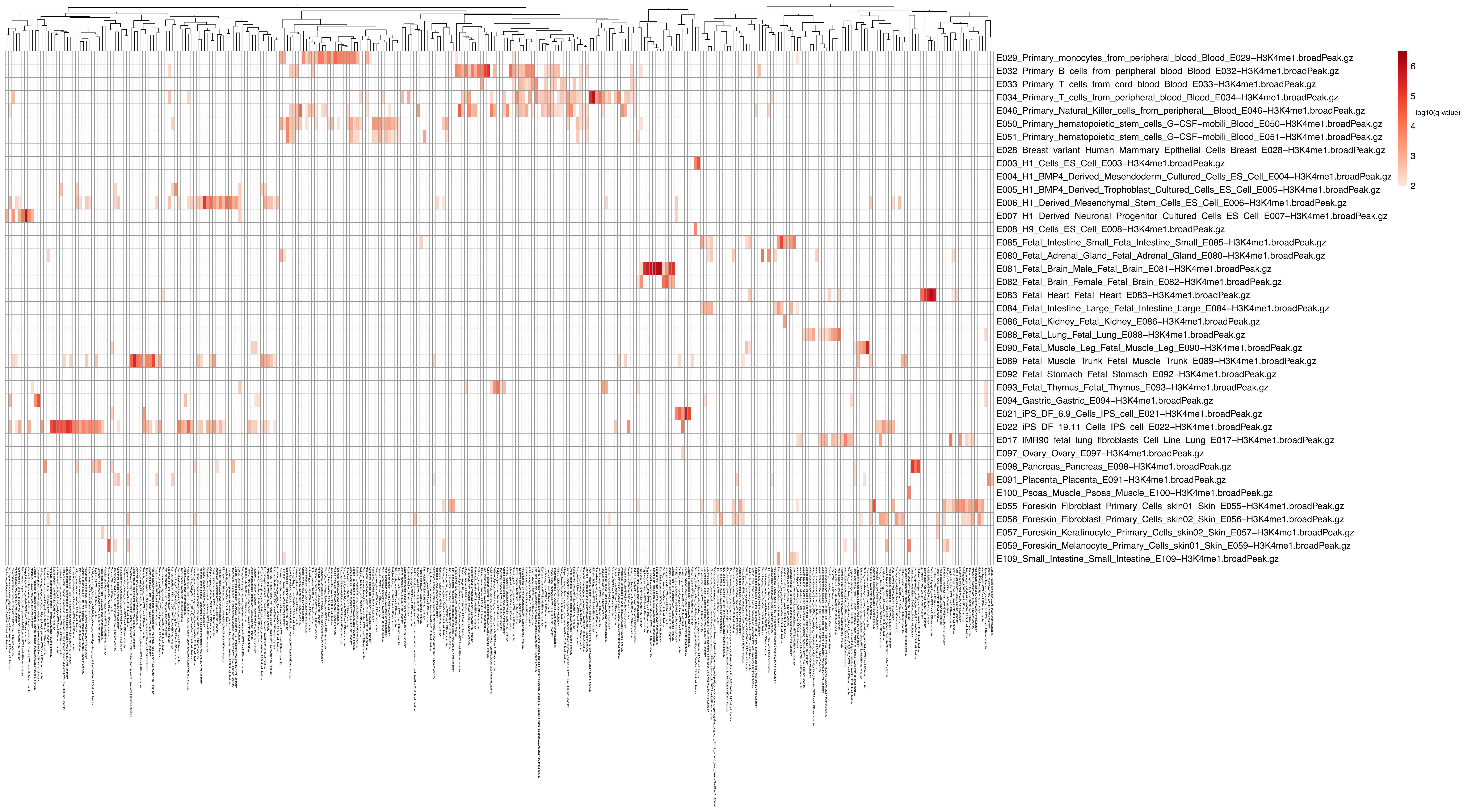

E029_Primary_monocytes_from_peripheral_blood_Blood_E029−H3K4me1.broadPeak.gz
E032_Primary_B_cells_from_peripheral_blood_Blood_E032−H3K4me1.broadPeak.gz
E033_Primary_T_cells_from_cord_blood_Blood_E033−H3K4me1.broadPeak.gz
E034_Primary_T_cells_from_peripheral_blood_Blood_E034−H3K4me1.broadPeak.gz
E046_Primary_Natural_Killer_cells_from_peripheral__Blood_E046−H3K4me1.broadPeak.gz
E050_Primary_hematopoietic_stem_cells_G-CSF-mobili_Blood_E050−H3K4me1.broadPeak.gz
E051_Primary_hematopoietic_stem_cells_G-CSF-mobili_Blood_E051−H3K4me1.broadPeak.gz
E028_Breast_variant_Human_Mammary_Epithelial_Cells_Breast_E028−H3K4me1.broadPeak.gz
E003_H1_Cells_ES_Cell_E003−H3K4me1.broadPeak.gz
E004_H1_BMP4_Derived_Mesendoderm_Cultured_Cells_ES_Cell_E004−H3K4me1.broadPeak.gz
E005_H1_BMP4_Derived_Trophoblast_Cultured_Cells_ES_Cell_E005−H3K4me1.broadPeak.gz
E006_H1_Derived_Mesenchymal_Stem_Cells_ES_Cell_E006−H3K4me1.broadPeak.gz
E007_H1_Derived_Neuronal_Progenitor_Cultured_Cells_ES_Cell_E007−H3K4me1.broadPeak.gz
E008_H9_Cells_ES_Cell_E008−H3K4me1.broadPeak.gz
E085_Fetal_Intestine_Small_Feta_Intestine_Small_E085−H3K4me1.broadPeak.gz
E080_Fetal_Adrenal_Gland_Fetal_Adrenal_Gland_E080−H3K4me1.broadPeak.gz
E081_Fetal_Brain_Male_Fetal_Brain_E081−H3K4me1.broadPeak.gz
E082_Fetal_Brain_Female_Fetal_Brain_E082−H3K4me1.broadPeak.gz
E083_Fetal_Heart_Fetal_Heart_E083−H3K4me1.broadPeak.gz
E084_Fetal_Intestine_Large_Fetal_Intestine_Large_E084−H3K4me1.broadPeak.gz
E086_Fetal_Kidney_Fetal_Kidney_E086−H3K4me1.broadPeak.gz
E088_Fetal_Lung_Fetal_Lung_E088−H3K4me1.broadPeak.gz
E090_Fetal_Muscle_Leg_Fetal_Muscle_Leg_E090−H3K4me1.broadPeak.gz
E089_Fetal_Muscle_Trunk_Fetal_Muscle_Trunk_E089−H3K4me1.broadPeak.gz
E092_Fetal_Stomach_Fetal_Stomach_E092−H3K4me1.broadPeak.gz
E093_Fetal_Thymus_Fetal_Thymus_E093−H3K4me1.broadPeak.gz
E094_Gastric_Gastric_E094−H3K4me1.broadPeak.gz
E021_iPS_DF_6.9_Cells_IPS_cell_E021−H3K4me1.broadPeak.gz
E022_iPS_DF_19.11_Cells_IPS_cell_E022−H3K4me1.broadPeak.gz
E017_IMR90_fetal_lung_fibroblasts_Cell_Line_Lung_E017−H3K4me1.broadPeak.gz
E097_Ovary_Ovary_E097−H3K4me1.broadPeak.gz
E098_Pancreas_Pancreas_E098−H3K4me1.broadPeak.gz
E091_Placenta_Placenta_E091−H3K4me1.broadPeak.gz
E100_Psoas_Muscle_Psoas_Muscle_E100−H3K4me1.broadPeak.gz
E055_Foreskin_Fibroblast_Primary_Cells_skin01_Skin_E055−H3K4me1.broadPeak.gz
E056_Foreskin_Fibroblast_Primary_Cells_skin02_Skin_E056−H3K4me1.broadPeak.gz
E057_Foreskin_Keratinocyte_Primary_Cells_skin02_Skin_E057−H3K4me1.broadPeak.gz
E059_Foreskin_Melanocyte_Primary_Cells_skin01_Skin_E059−H3K4me1.broadPeak.gz
E109_Small_Intestine_Small_Intestine_E109−H3K4me1.broadPeak.gz
6
5
-log10(q-value)
4
3
2

Figure S5

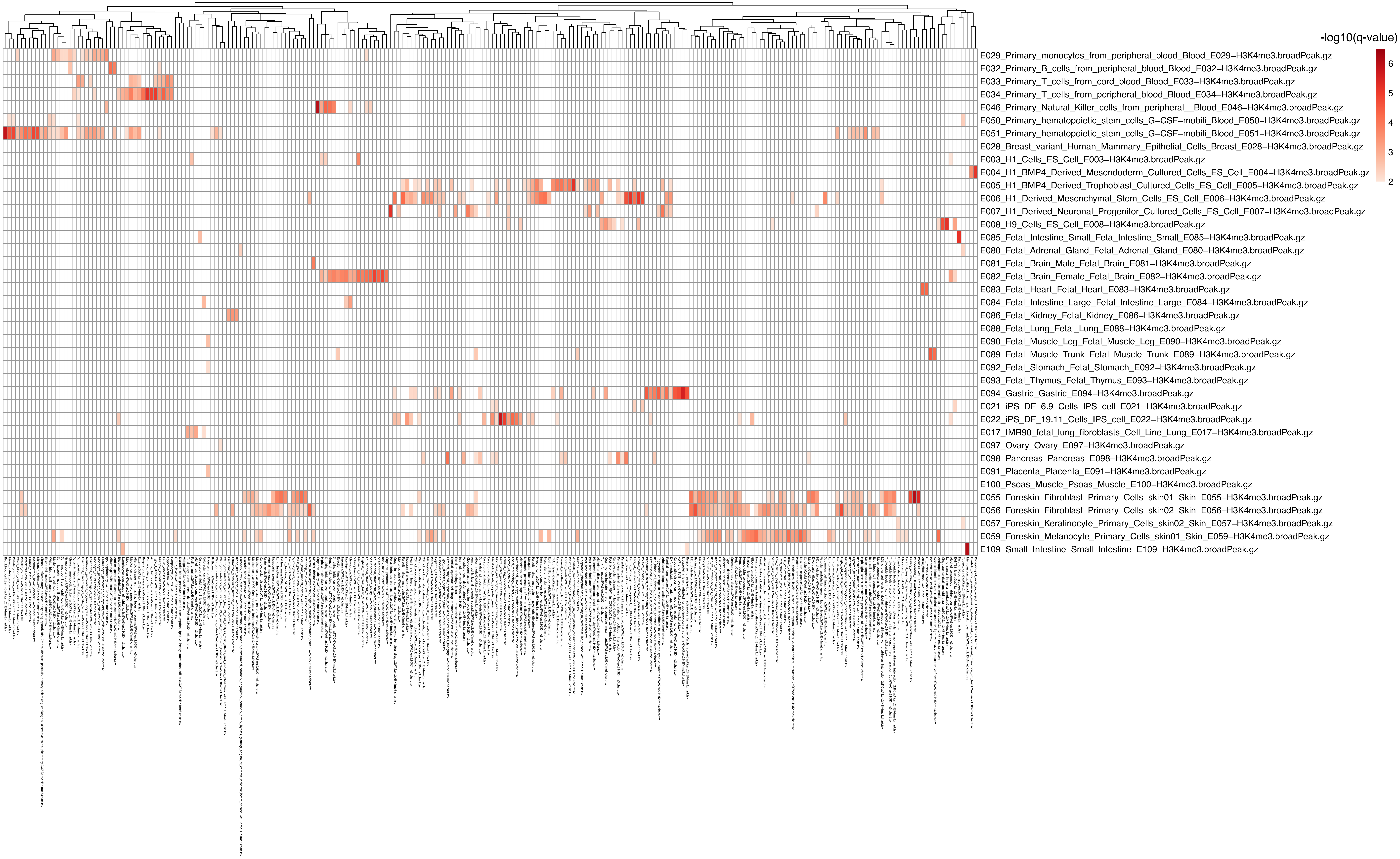
-log10(q-value)
6
5
4
3
2
E029_Primary_monocytes_from_peripheral_blood_Blood_E029–H3K4me3.broadPeak.gz
E032_Primary_B_cells_from_peripheral_blood_Blood_E032–H3K4me3.broadPeak.gz
E033_Primary_T_cells_from_cord_blood_Blood_E033–H3K4me3.broadPeak.gz
E034_Primary_T_cells_from_peripheral_blood_Blood_E034–H3K4me3.broadPeak.gz
E046_Primary_Natural_Killer_cells_from_peripheral__Blood_E046–H3K4me3.broadPeak.gz
E050_Primary_hematopoietic_stem_cells_G–CSF–mobili_Blood_E050–H3K4me3.broadPeak.gz
E051_Primary_hematopoietic_stem_cells_G–CSF–mobili_Blood_E051–H3K4me3.broadPeak.gz
E028_Breast_variant_Human_Mammary_Epithelial_Cells_Breast_E028–H3K4me3.broadPeak.gz
E003_H1_Cells_ES_Cell_E003–H3K4me3.broadPeak.gz
E004_H1_BMP4_Derived_Mesendoderm_Cultured_Cells_ES_Cell_E004–H3K4me3.broadPeak.gz
E005_H1_BMP4_Derived_Trophoblast_Cultured_Cells_ES_Cell_E005–H3K4me3.broadPeak.gz
E006_H1_Derived_Mesenchymal_Stem_Cells_ES_Cell_E006–H3K4me3.broadPeak.gz
E007_H1_Derived_Neuronal_Progenitor_Cultured_Cells_ES_Cell_E007–H3K4me3.broadPeak.gz
E008_H9_Cells_ES_Cell_E008–H3K4me3.broadPeak.gz
E085_Fetal_Intestine_Small_Feta_Intestine_Small_E085–H3K4me3.broadPeak.gz
E080_Fetal_Adrenal_Gland_Fetal_Adrenal_Gland_E080–H3K4me3.broadPeak.gz
E081_Fetal_Brain_Male_Fetal_Brain_E081–H3K4me3.broadPeak.gz
E082_Fetal_Brain_Female_Fetal_Brain_E082–H3K4me3.broadPeak.gz
E083_Fetal_Heart_Fetal_Heart_E083–H3K4me3.broadPeak.gz
E084_Fetal_Intestine_Large_Fetal_Intestine_Large_E084–H3K4me3.broadPeak.gz
E086_Fetal_Kidney_Fetal_Kidney_E086–H3K4me3.broadPeak.gz
E088_Fetal_Lung_Fetal_Lung_E088–H3K4me3.broadPeak.gz
E090_Fetal_Muscle_Leg_Fetal_Muscle_Leg_E090–H3K4me3.broadPeak.gz
E089_Fetal_Muscle_Trunk_Fetal_Muscle_Trunk_E089–H3K4me3.broadPeak.gz
E092_Fetal_Stomach_Fetal_Stomach_E092–H3K4me3.broadPeak.gz
E093_Fetal_Thymus_Fetal_Thymus_E093–H3K4me3.broadPeak.gz
E094_Gastric_Gastric_E094–H3K4me3.broadPeak.gz
E021_iPS_DF_6.9_Cells_IPS_cell_E021–H3K4me3.broadPeak.gz
E022_iPS_DF_19.11_Cells_IPS_cell_E022–H3K4me3.broadPeak.gz
E017_IMR90_fetal_lung_fibroblasts_Cell_Line_Lung_E017–H3K4me3.broadPeak.gz
E097_Ovary_Ovary_E097–H3K4me3.broadPeak.gz
E098_Pancreas_Pancreas_E098–H3K4me3.broadPeak.gz
E091_Placenta_Placenta_E091–H3K4me3.broadPeak.gz
E100_Psoas_Muscle_Psoas_Muscle_E100–H3K4me3.broadPeak.gz
E055_Foreskin_Fibroblast_Primary_Cells_skin01_Skin_E055–H3K4me3.broadPeak.gz
E056_Foreskin_Fibroblast_Primary_Cells_skin02_Skin_E056–H3K4me3.broadPeak.gz
E057_Foreskin_Keratinocyte_Primary_Cells_skin02_Skin_E057–H3K4me3.broadPeak.gz
E059_Foreskin_Melanocyte_Primary_Cells_skin01_Skin_E059–H3K4me3.broadPeak.gz
E109_Small_Intestine_Small_Intestine_E109–H3K4me3.broadPeak.gz

Figure S6

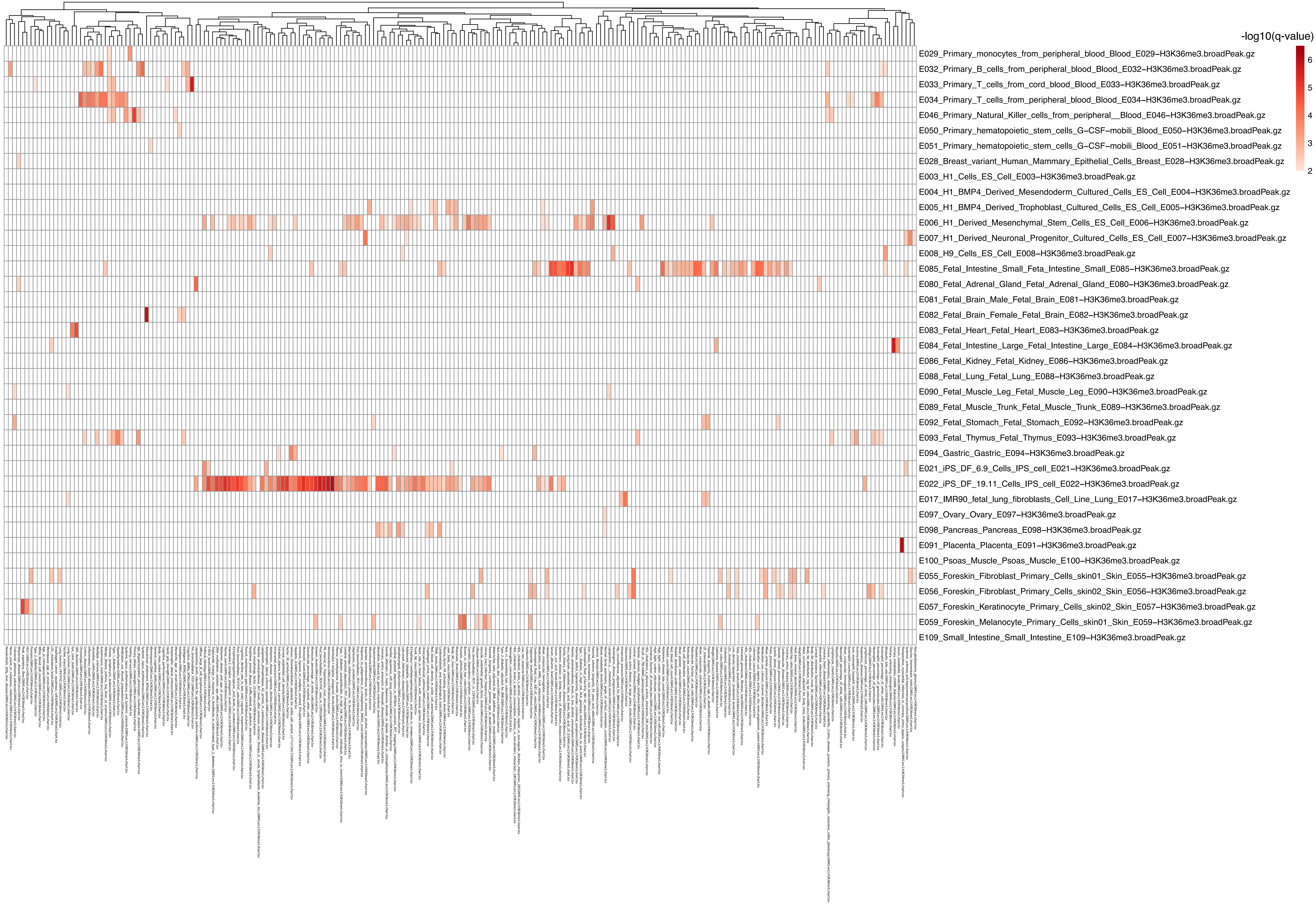
-log10(q-value)
6
5
4
3
2
E029_Primary_monocytes_from_peripheral_blood_Blood_E029–H3K36me3.broadPeak.gz
E032_Primary_B_cells_from_peripheral_blood_Blood_E032–H3K36me3.broadPeak.gz
E033_Primary_T_cells_from_cord_blood_Blood_E033–H3K36me3.broadPeak.gz
E034_Primary_T_cells_from_peripheral_blood_Blood_E034–H3K36me3.broadPeak.gz
E046_Primary_Natural_Killer_cells_from_peripheral__Blood_E046–H3K36me3.broadPeak.gz
E050_Primary_hematopoietic_stem_cells_G–CSF–mobili_Blood_E050–H3K36me3.broadPeak.gz
E051_Primary_hematopoietic_stem_cells_G–CSF–mobili_Blood_E051–H3K36me3.broadPeak.gz
E028_Breast_variant_Human_Mammary_Epithelial_Cells_Breast_E028–H3K36me3.broadPeak.gz
E003_H1_Cells_ES_Cell_E003–H3K36me3.broadPeak.gz
E004_H1_BMP4_Derived_Mesendoderm_Cultured_Cells_ES_Cell_E004–H3K36me3.broadPeak.gz
E005_H1_BMP4_Derived_Trophoblast_Cultured_Cells_ES_Cell_E005–H3K36me3.broadPeak.gz
E006_H1_Derived_Mesenchymal_Stem_Cells_ES_Cell_E006–H3K36me3.broadPeak.gz
E007_H1_Derived_Neuronal_Progenitor_Cultured_Cells_ES_Cell_E007–H3K36me3.broadPeak.gz
E008_H9_Cells_ES_Cell_E008–H3K36me3.broadPeak.gz
E085_Fetal_Intestine_Small_Feta_Intestine_Small_E085–H3K36me3.broadPeak.gz
E080_Fetal_Adrenal_Gland_Fetal_Adrenal_Gland_E080–H3K36me3.broadPeak.gz
E081_Fetal_Brain_Male_Fetal_Brain_E081–H3K36me3.broadPeak.gz
E082_Fetal_Brain_Female_Fetal_Brain_E082–H3K36me3.broadPeak.gz
E083_Fetal_Heart_Fetal_Heart_E083–H3K36me3.broadPeak.gz
E084_Fetal_Intestine_Large_Fetal_Intestine_Large_E084–H3K36me3.broadPeak.gz
E086_Fetal_Kidney_Fetal_Kidney_E086–H3K36me3.broadPeak.gz
E088_Fetal_Lung_Fetal_Lung_E088–H3K36me3.broadPeak.gz
E090_Fetal_Muscle_Leg_Fetal_Muscle_Leg_E090–H3K36me3.broadPeak.gz
E089_Fetal_Muscle_Trunk_Fetal_Muscle_Trunk_E089–H3K36me3.broadPeak.gz
E092_Fetal_Stomach_Fetal_Stomach_E092–H3K36me3.broadPeak.gz
E093_Fetal_Thymus_Fetal_Thymus_E093–H3K36me3.broadPeak.gz
E094_Gastric_Gastric_E094–H3K36me3.broadPeak.gz
E021_iPS_DF_6.9_Cells_IPS_cell_E021–H3K36me3.broadPeak.gz
E022_iPS_DF_19.11_Cells_IPS_cell_E022–H3K36me3.broadPeak.gz
E017_IMR90_fetal_lung_fibroblasts_Cell_Line_Lung_E017–H3K36me3.broadPeak.gz
E097_Ovary_Ovary_E097–H3K36me3.broadPeak.gz
E098_Pancreas_Pancreas_E098–H3K36me3.broadPeak.gz
E091_Placenta_Placenta_E091–H3K36me3.broadPeak.gz
E100_Psoas_Muscle_Psoas_Muscle_E100–H3K36me3.broadPeak.gz
E055_Foreskin_Fibroblast_Primary_Cells_skin01_Skin_E055–H3K36me3.broadPeak.gz
E056_Foreskin_Fibroblast_Primary_Cells_skin02_Skin_E056–H3K36me3.broadPeak.gz
E057_Foreskin_Keratinocyte_Primary_Cells_skin02_Skin_E057–H3K36me3.broadPeak.gz
E059_Foreskin_Melanocyte_Primary_Cells_skin01_Skin_E059–H3K36me3.broadPeak.gz
E109_Small_Intestine_Small_Intestine_E109–H3K36me3.broadPeak.gz

Figure S7

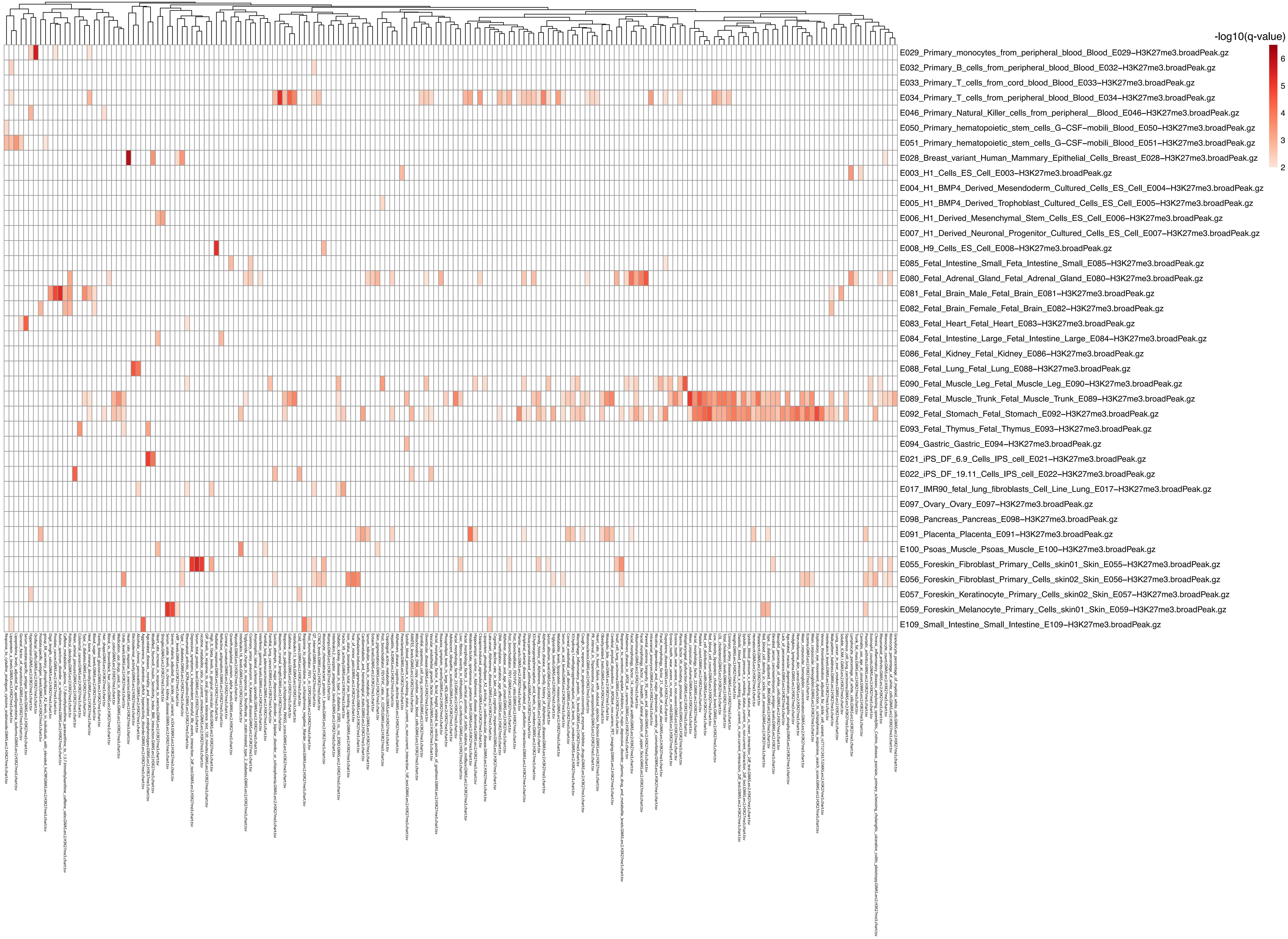
-log10(q-value)
6
5
4
3
2
E029_Primary_monocytes_from_peripheral_blood_Blood_E029–H3K27me3.broadPeak.gz
E032_Primary_B_cells_from_peripheral_blood_Blood_E032–H3K27me3.broadPeak.gz
E033_Primary_T_cells_from_cord_blood_Blood_E033–H3K27me3.broadPeak.gz
E034_Primary_T_cells_from_peripheral_blood_Blood_E034–H3K27me3.broadPeak.gz
E046_Primary_Natural_Killer_cells_from_peripheral__Blood_E046–H3K27me3.broadPeak.gz
E050_Primary_hematopoietic_stem_cells_G–CSF–mobili_Blood_E050–H3K27me3.broadPeak.gz
E051_Primary_hematopoietic_stem_cells_G–CSF–mobili_Blood_E051–H3K27me3.broadPeak.gz
E028_Breast_variant_Human_Mammary_Epithelial_Cells_Breast_E028–H3K27me3.broadPeak.gz
E003_H1_Cells_ES_Cell_E003–H3K27me3.broadPeak.gz
E004_H1_BMP4_Derived_Mesendoderm_Cultured_Cells_ES_Cell_E004–H3K27me3.broadPeak.gz
E005_H1_BMP4_Derived_Trophoblast_Cultured_Cells_ES_Cell_E005–H3K27me3.broadPeak.gz
E006_H1_Derived_Mesenchymal_Stem_Cells_ES_Cell_E006–H3K27me3.broadPeak.gz
E007_H1_Derived_Neuronal_Progenitor_Cultured_Cells_ES_Cell_E007–H3K27me3.broadPeak.gz
E008_H9_Cells_ES_Cell_E008–H3K27me3.broadPeak.gz
E085_Fetal_Intestine_Small_Feta_Intestine_Small_E085–H3K27me3.broadPeak.gz
E080_Fetal_Adrenal_Gland_Fetal_Adrenal_Gland_E080–H3K27me3.broadPeak.gz
E081_Fetal_Brain_Male_Fetal_Brain_E081–H3K27me3.broadPeak.gz
E082_Fetal_Brain_Female_Fetal_Brain_E082–H3K27me3.broadPeak.gz
E083_Fetal_Heart_Fetal_Heart_E083–H3K27me3.broadPeak.gz
E084_Fetal_Intestine_Large_Fetal_Intestine_Large_E084–H3K27me3.broadPeak.gz
E086_Fetal_Kidney_Fetal_Kidney_E086–H3K27me3.broadPeak.gz
E088_Fetal_Lung_Fetal_Lung_E088–H3K27me3.broadPeak.gz
E090_Fetal_Muscle_Leg_Fetal_Muscle_Leg_E090–H3K27me3.broadPeak.gz
E089_Fetal_Muscle_Trunk_Fetal_Muscle_Trunk_E089–H3K27me3.broadPeak.gz
E092_Fetal_Stomach_Fetal_Stomach_E092–H3K27me3.broadPeak.gz
E093_Fetal_Thymus_Fetal_Thymus_E093–H3K27me3.broadPeak.gz
E094_Gastric_Gastric_E094–H3K27me3.broadPeak.gz
E021_iPS_DF_6.9_Cells_IPS_cell_E021–H3K27me3.broadPeak.gz
E022_iPS_DF_19.11_Cells_IPS_cell_E022–H3K27me3.broadPeak.gz
E017_IMR90_fetal_lung_fibroblasts_Cell_Line_Lung_E017–H3K27me3.broadPeak.gz
E097_Ovary_Ovary_E097–H3K27me3.broadPeak.gz
E098_Pancreas_Pancreas_E098–H3K27me3.broadPeak.gz
E091_Placenta_Placenta_E091–H3K27me3.broadPeak.gz
E100_Psoas_Muscle_Psoas_Muscle_E100–H3K27me3.broadPeak.gz
E055_Foreskin_Fibroblast_Primary_Cells_skin01_Skin_E055–H3K27me3.broadPeak.gz
E056_Foreskin_Fibroblast_Primary_Cells_skin02_Skin_E056–H3K27me3.broadPeak.gz
E057_Foreskin_Keratinocyte_Primary_Cells_skin02_Skin_E057–H3K27me3.broadPeak.gz
E059_Foreskin_Melanocyte_Primary_Cells_skin01_Skin_E059–H3K27me3.broadPeak.gz
E109_Small_Intestine_Small_Intestine_E109–H3K27me3.broadPeak.gz

Figure S8

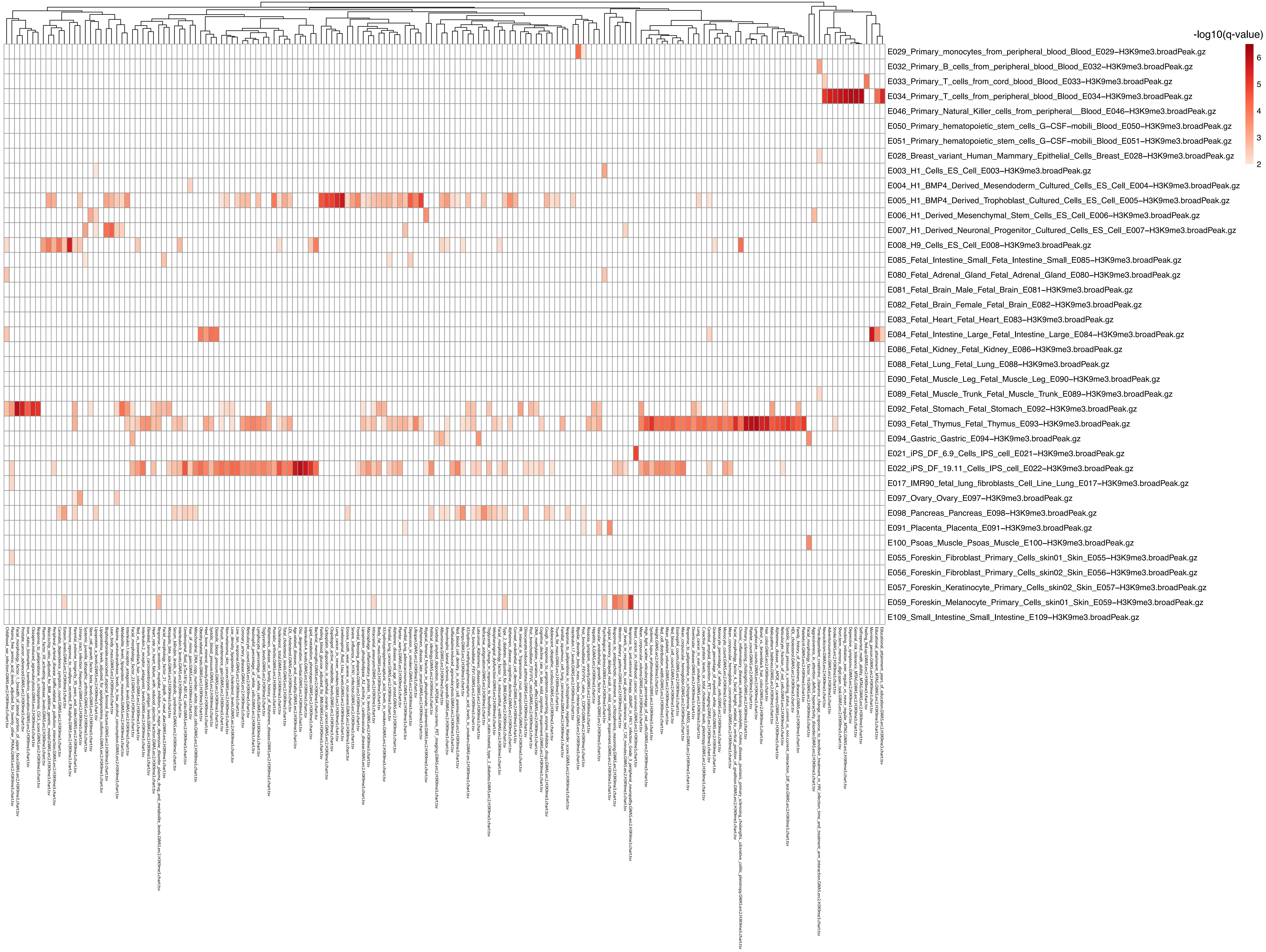
-log10(q-value)
6
5
4
3
2
E029_Primary_monocytes_from_peripheral_blood_Blood_E029–H3K9me3.broadPeak.gz
E032_Primary_B_cells_from_peripheral_blood_Blood_E032–H3K9me3.broadPeak.gz
E033_Primary_T_cells_from_cord_blood_Blood_E033–H3K9me3.broadPeak.gz
E034_Primary_T_cells_from_peripheral_blood_Blood_E034–H3K9me3.broadPeak.gz
E046_Primary_Natural_Killer_cells_from_peripheral__Blood_E046–H3K9me3.broadPeak.gz
E050_Primary_hematopoietic_stem_cells_G–CSF–mobili_Blood_E050–H3K9me3.broadPeak.gz
E051_Primary_hematopoietic_stem_cells_G–CSF–mobili_Blood_E051–H3K9me3.broadPeak.gz
E028_Breast_variant_Human_Mammary_Epithelial_Cells_Breast_E028–H3K9me3.broadPeak.gz
E003_H1_Cells_ES_Cell_E003–H3K9me3.broadPeak.gz
E004_H1_BMP4_Derived_Mesendoderm_Cultured_Cells_ES_Cell_E004–H3K9me3.broadPeak.gz
E005_H1_BMP4_Derived_Trophoblast_Cultured_Cells_ES_Cell_E005–H3K9me3.broadPeak.gz
E006_H1_Derived_Mesenchymal_Stem_Cells_ES_Cell_E006–H3K9me3.broadPeak.gz
E007_H1_Derived_Neuronal_Progenitor_Cultured_Cells_ES_Cell_E007–H3K9me3.broadPeak.gz
E008_H9_Cells_ES_Cell_E008–H3K9me3.broadPeak.gz
E085_Fetal_Intestine_Small_Feta_Intestine_Small_E085–H3K9me3.broadPeak.gz
E080_Fetal_Adrenal_Gland_Fetal_Adrenal_Gland_E080–H3K9me3.broadPeak.gz
E081_Fetal_Brain_Male_Fetal_Brain_E081–H3K9me3.broadPeak.gz
E082_Fetal_Brain_Female_Fetal_Brain_E082–H3K9me3.broadPeak.gz
E083_Fetal_Heart_Fetal_Heart_E083–H3K9me3.broadPeak.gz
E084_Fetal_Intestine_Large_Fetal_Intestine_Large_E084–H3K9me3.broadPeak.gz
E086_Fetal_Kidney_Fetal_Kidney_E086–H3K9me3.broadPeak.gz
E088_Fetal_Lung_Fetal_Lung_E088–H3K9me3.broadPeak.gz
E090_Fetal_Muscle_Leg_Fetal_Muscle_Leg_E090–H3K9me3.broadPeak.gz
E089_Fetal_Muscle_Trunk_Fetal_Muscle_Trunk_E089–H3K9me3.broadPeak.gz
E092_Fetal_Stomach_Fetal_Stomach_E092–H3K9me3.broadPeak.gz
E093_Fetal_Thymus_Fetal_Thymus_E093–H3K9me3.broadPeak.gz
E094_Gastric_Gastric_E094–H3K9me3.broadPeak.gz
E021_iPS_DF_6.9_Cells_IPS_cell_E021–H3K9me3.broadPeak.gz
E022_iPS_DF_19.11_Cells_IPS_cell_E022–H3K9me3.broadPeak.gz
E017_IMR90_fetal_lung_fibroblasts_Cell_Line_Lung_E017–H3K9me3.broadPeak.gz
E097_Ovary_Ovary_E097–H3K9me3.broadPeak.gz
E098_Pancreas_Pancreas_E098–H3K9me3.broadPeak.gz
E091_Placenta_Placenta_E091–H3K9me3.broadPeak.gz
E100_Psoas_Muscle_Psoas_Muscle_E100–H3K9me3.broadPeak.gz
E055_Foreskin_Fibroblast_Primary_Cells_skin01_Skin_E055–H3K9me3.broadPeak.gz
E056_Foreskin_Fibroblast_Primary_Cells_skin02_Skin_E056–H3K9me3.broadPeak.gz
E057_Foreskin_Keratinocyte_Primary_Cells_skin02_Skin_E057–H3K9me3.broadPeak.gz
E059_Foreskin_Melanocyte_Primary_Cells_skin01_Skin_E059–H3K9me3.broadPeak.gz
E109_Small_Intestine_Small_Intestine_E109–H3K9me3.broadPeak.gz

Figure S9

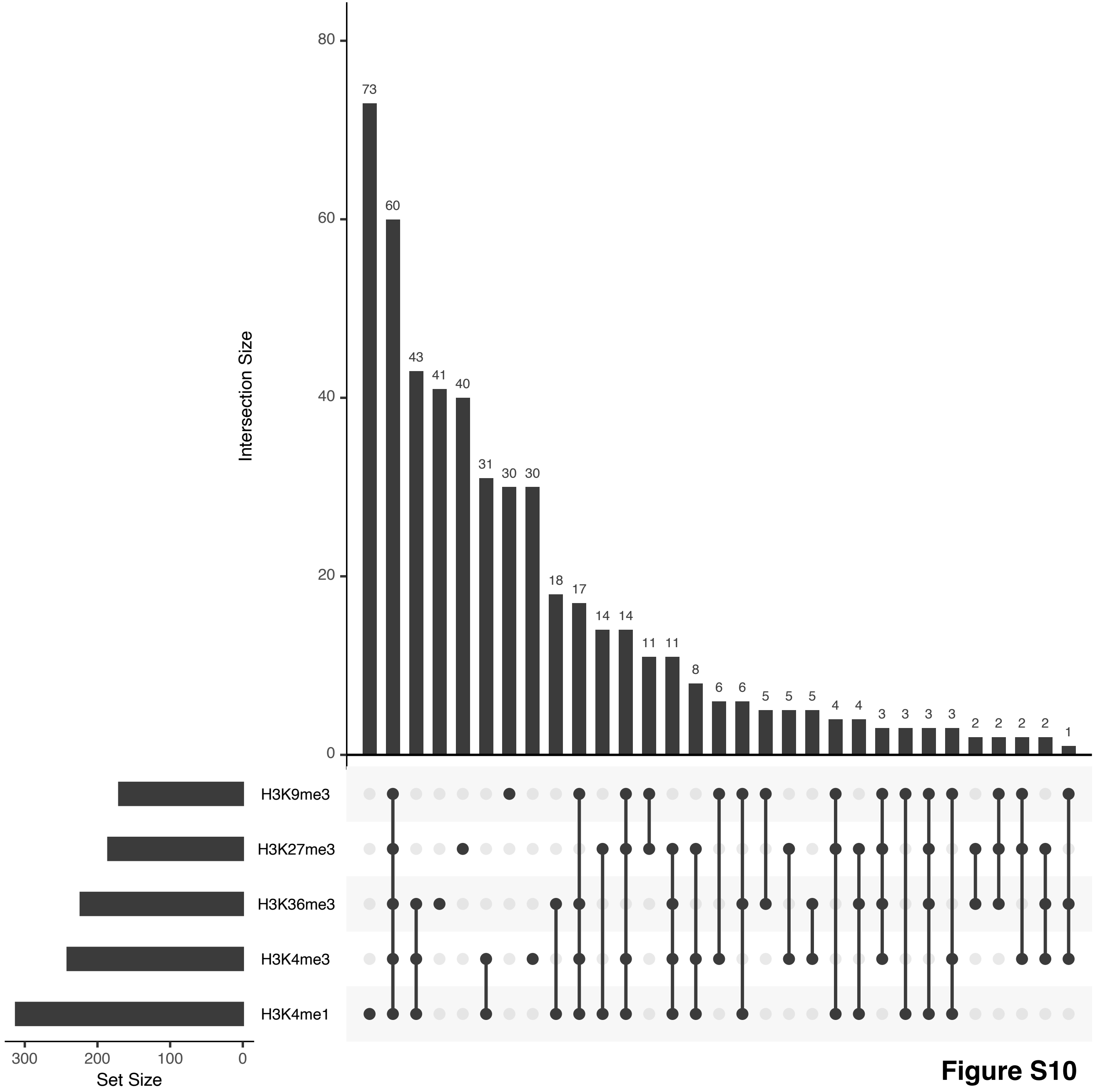

Intersection Size
80
60
40
20
0
73
60
43
41
40
31
30
30
18
17
14
14
11
11
8
6
6
5
5
5
4
4
3
3
3
3
2
2
2
2
1
H3K9me3
H3K27me3
H3K36me3
H3K4me3
H3K4me1
300
200
100
0
Set Size

Figure S10

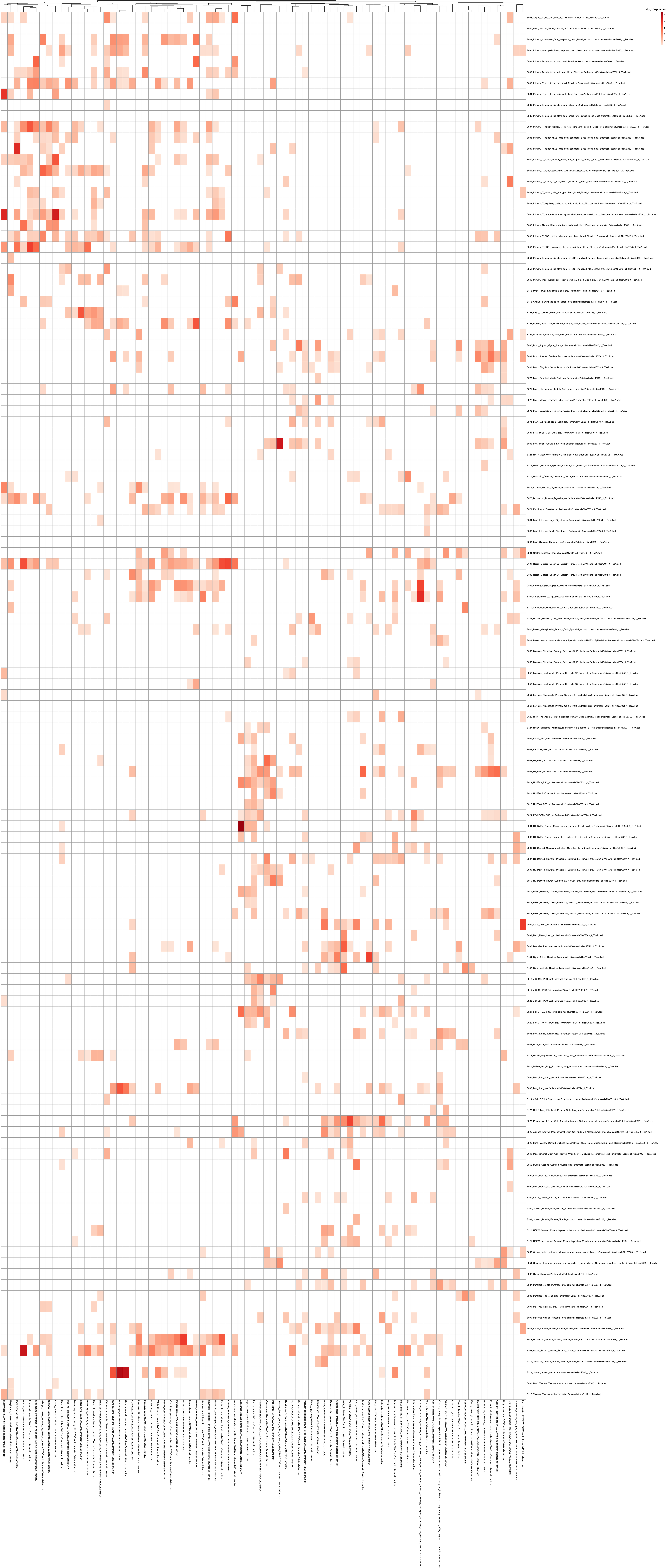

Figure S11

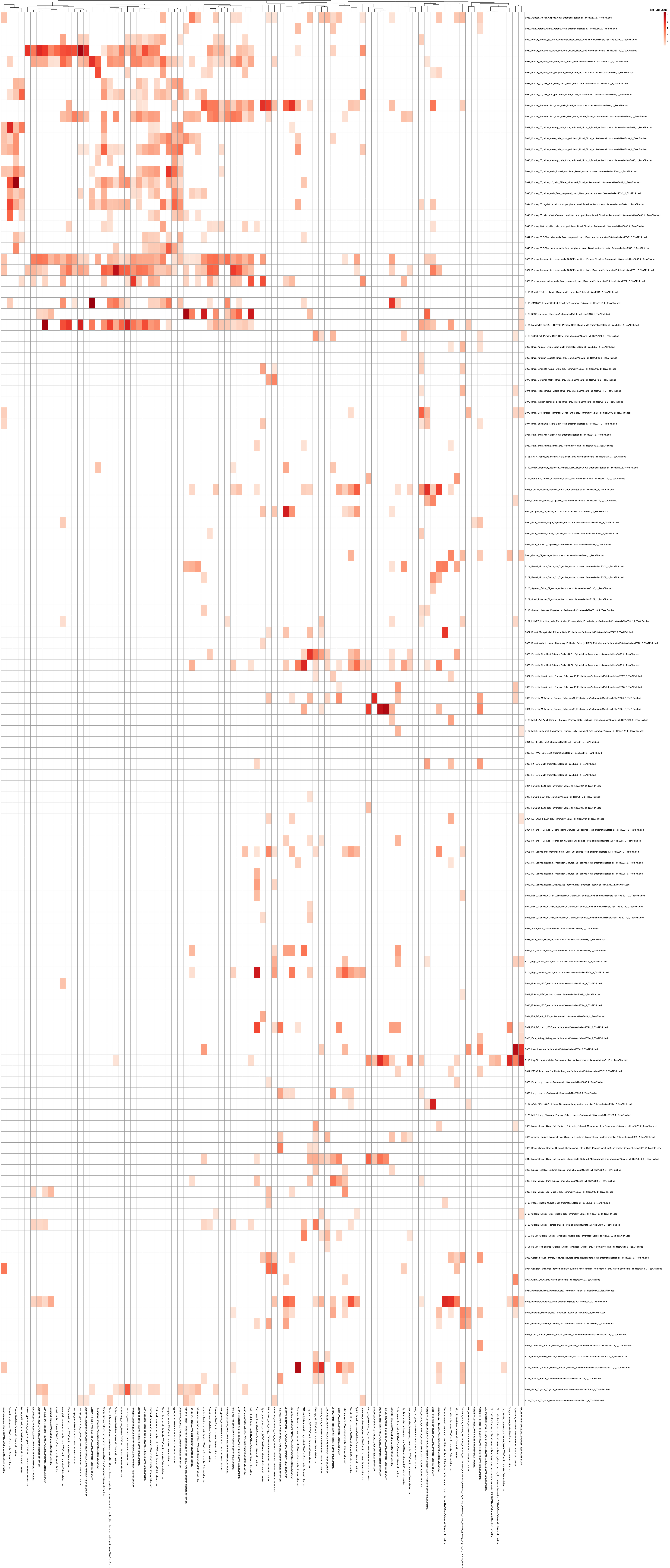

Figure S12

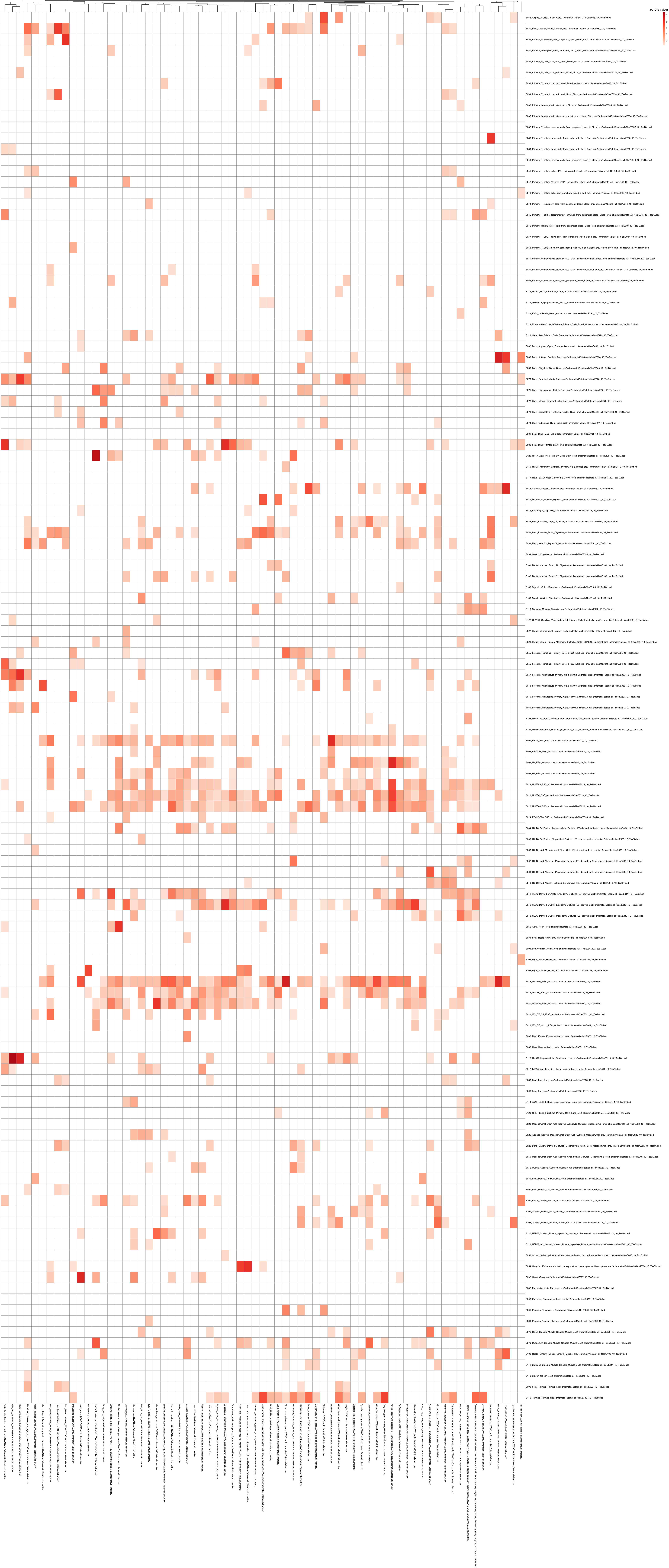

Figure S13

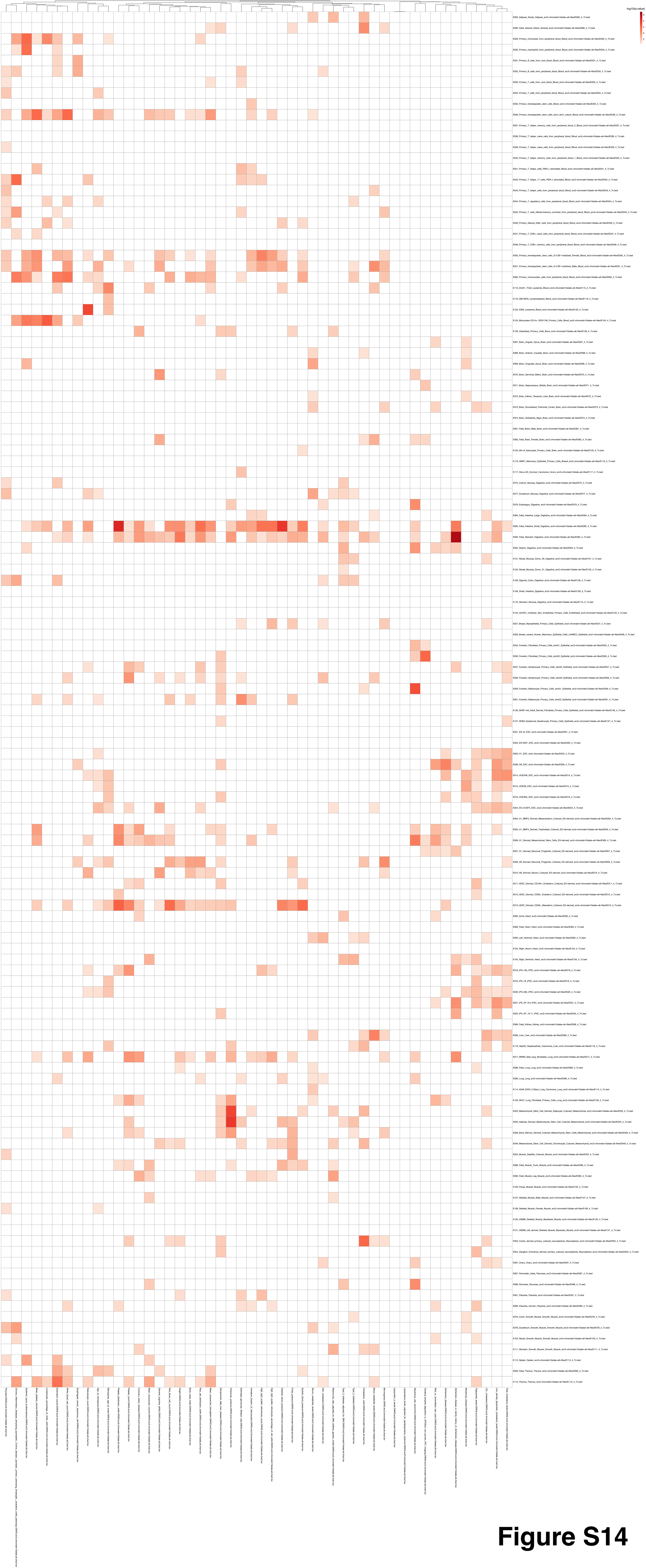

Figure S14

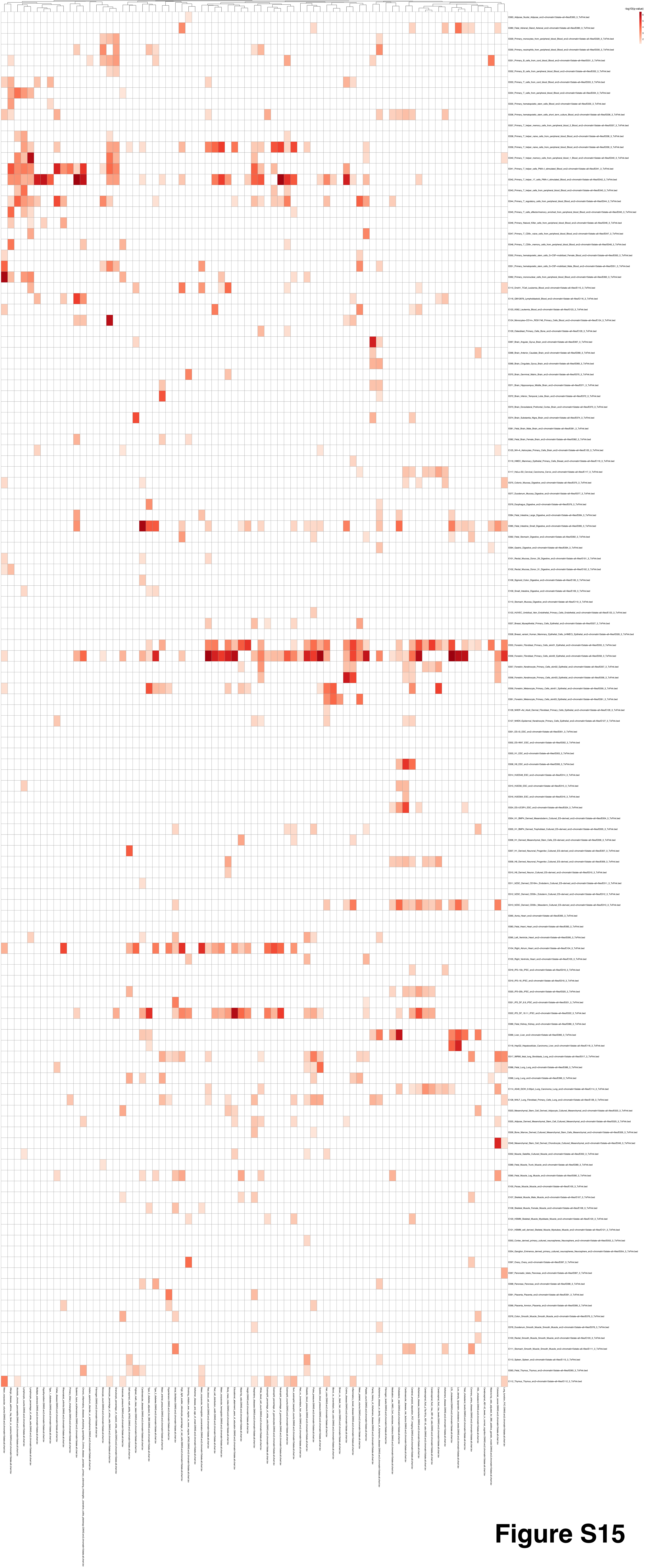

Figure S15

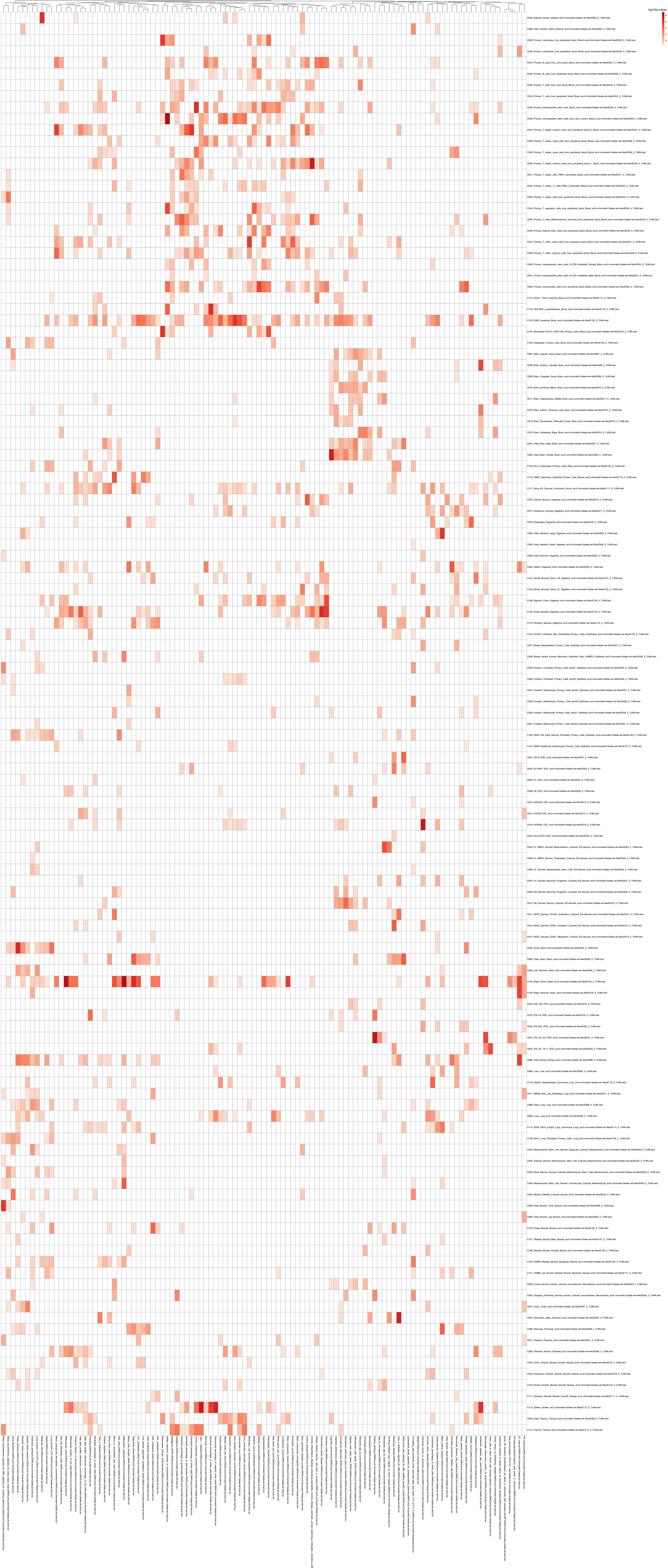

Figure S16

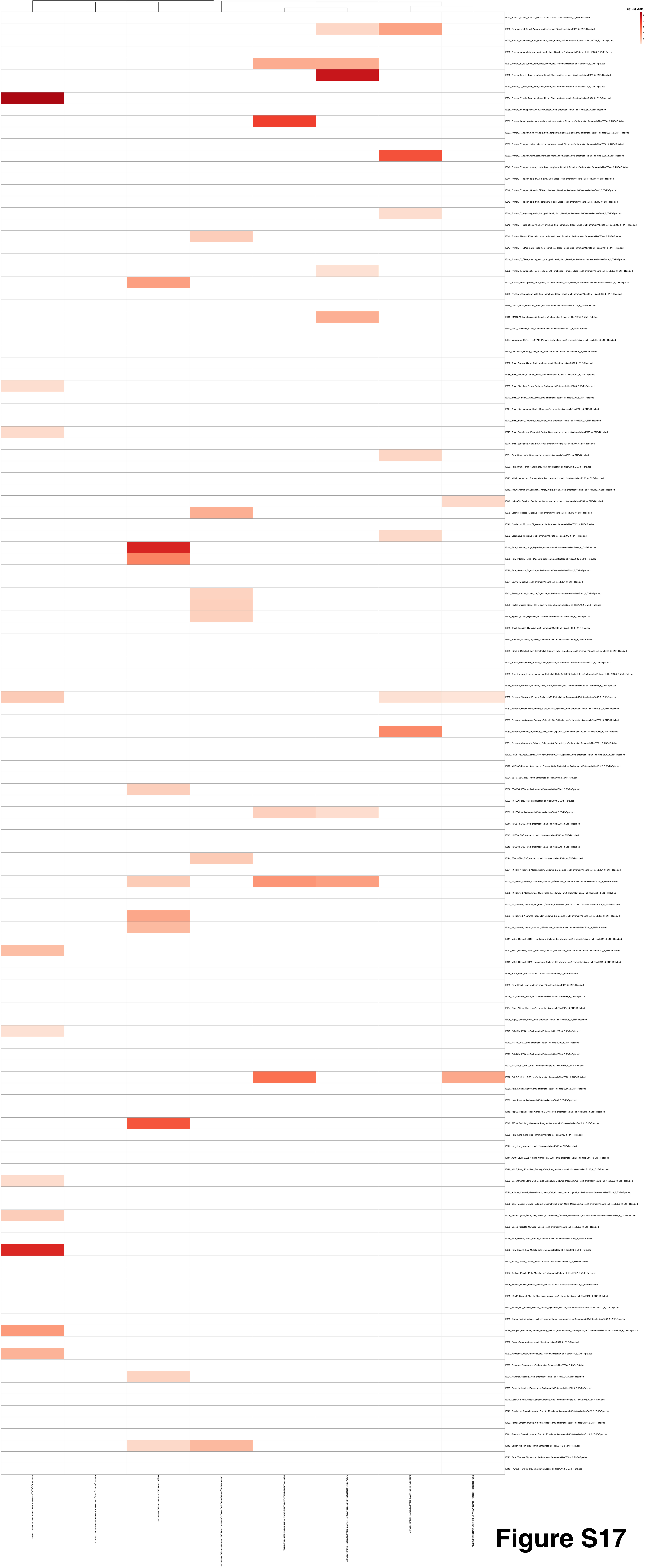

Figure S17

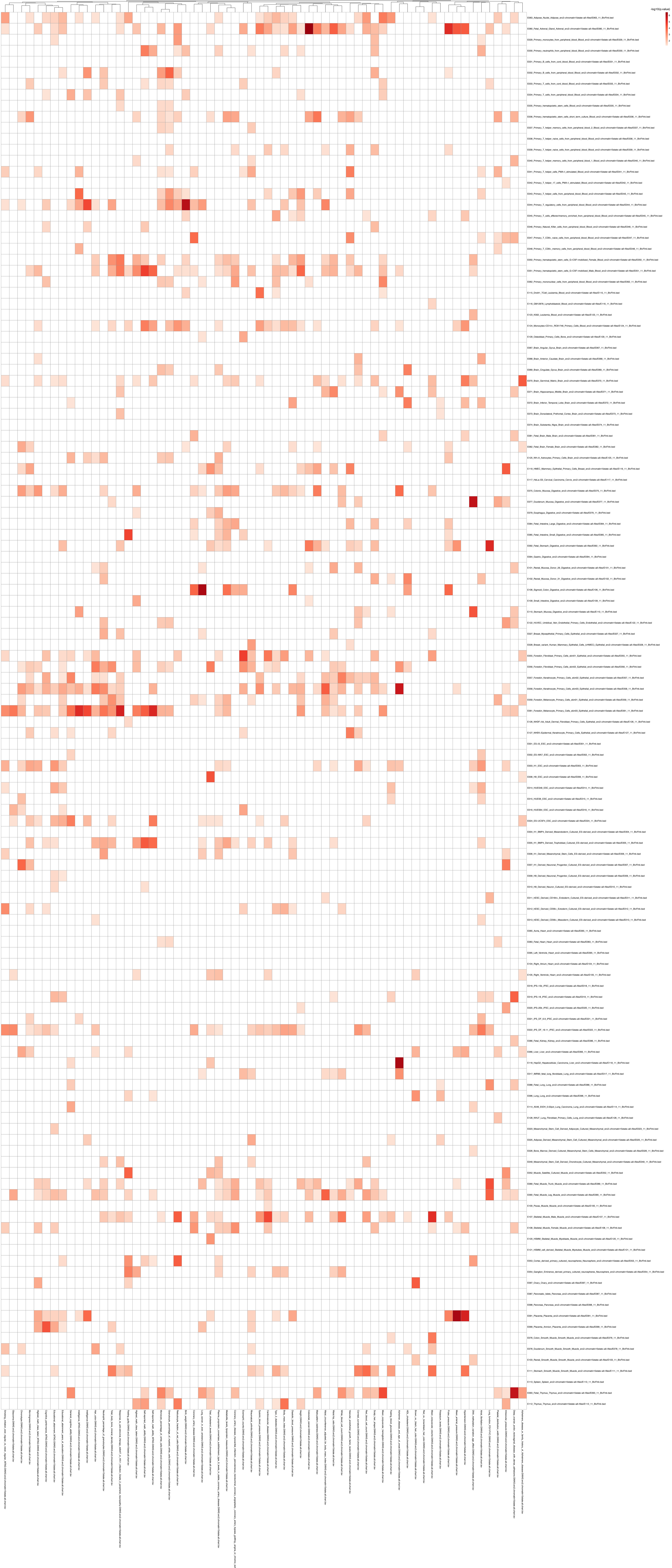

Figure S18

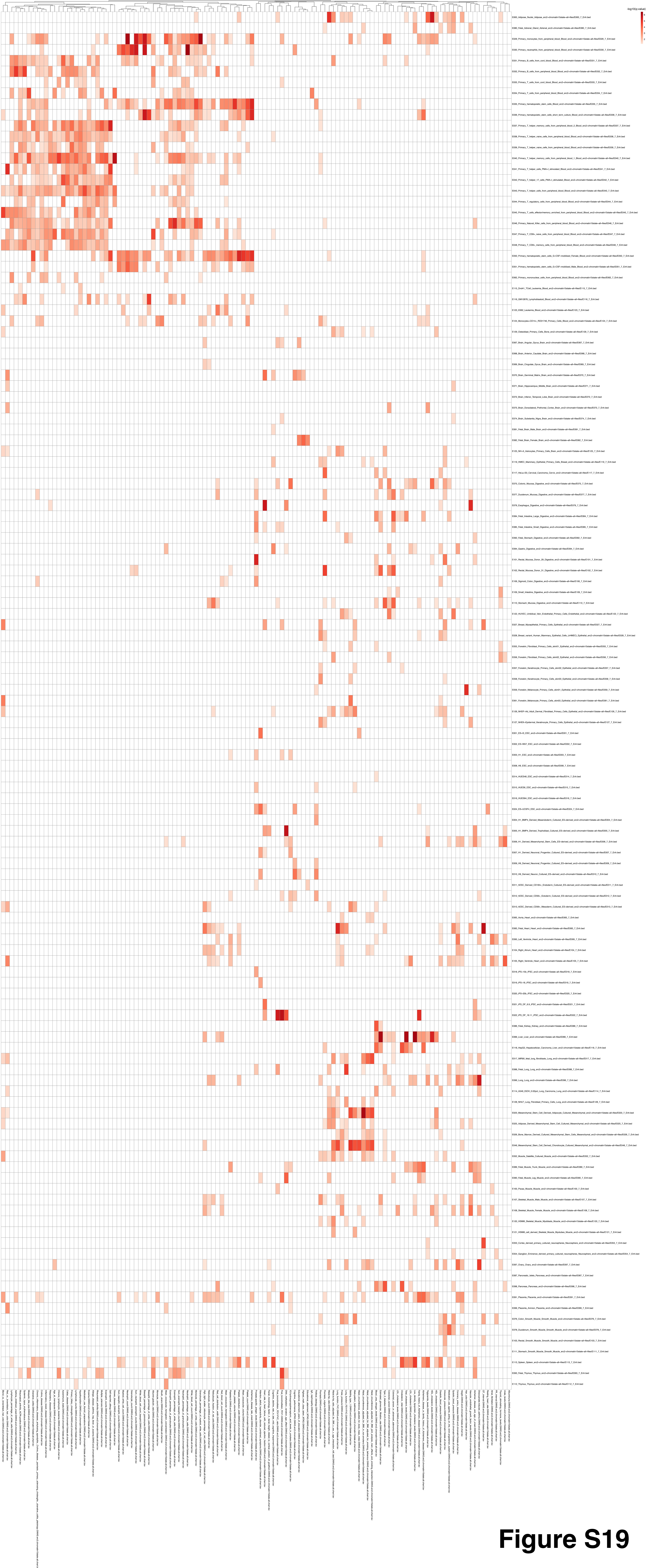

Figure S19

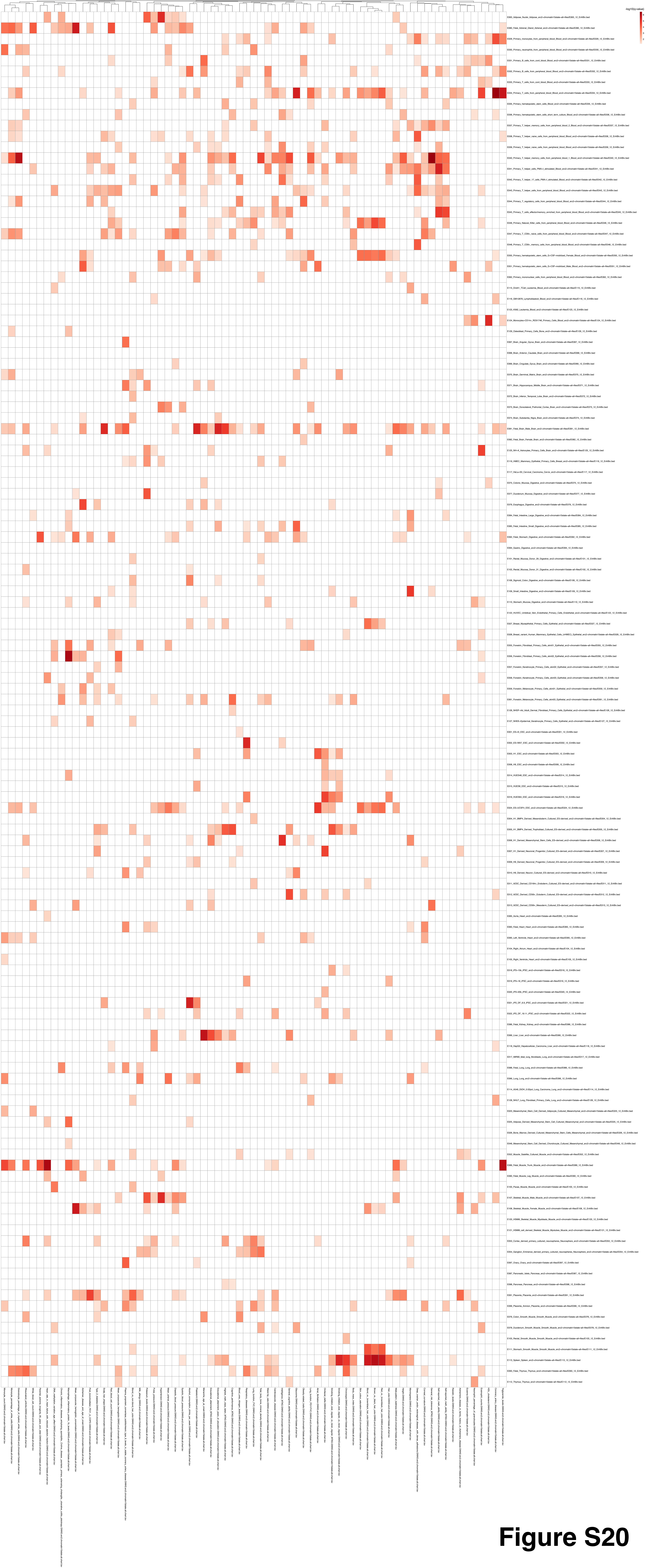

Figure S20

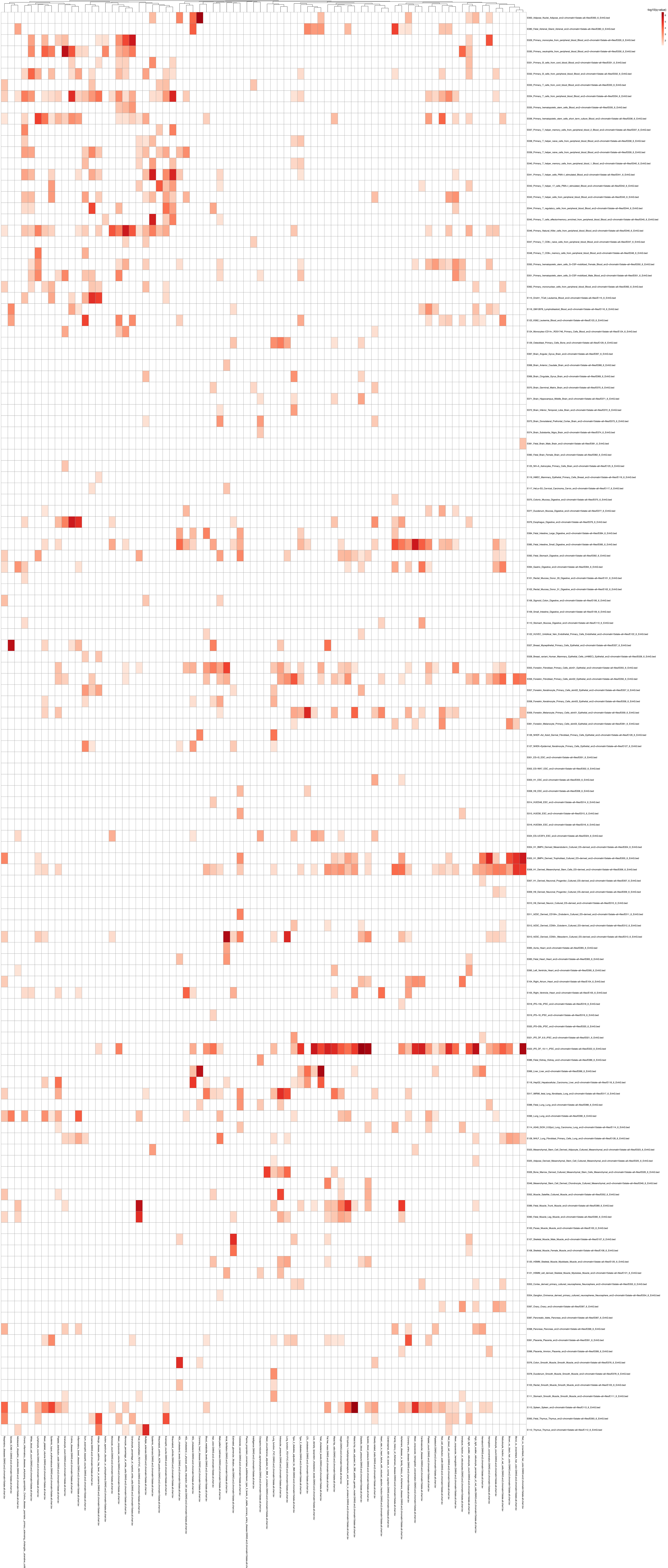

Figure S21

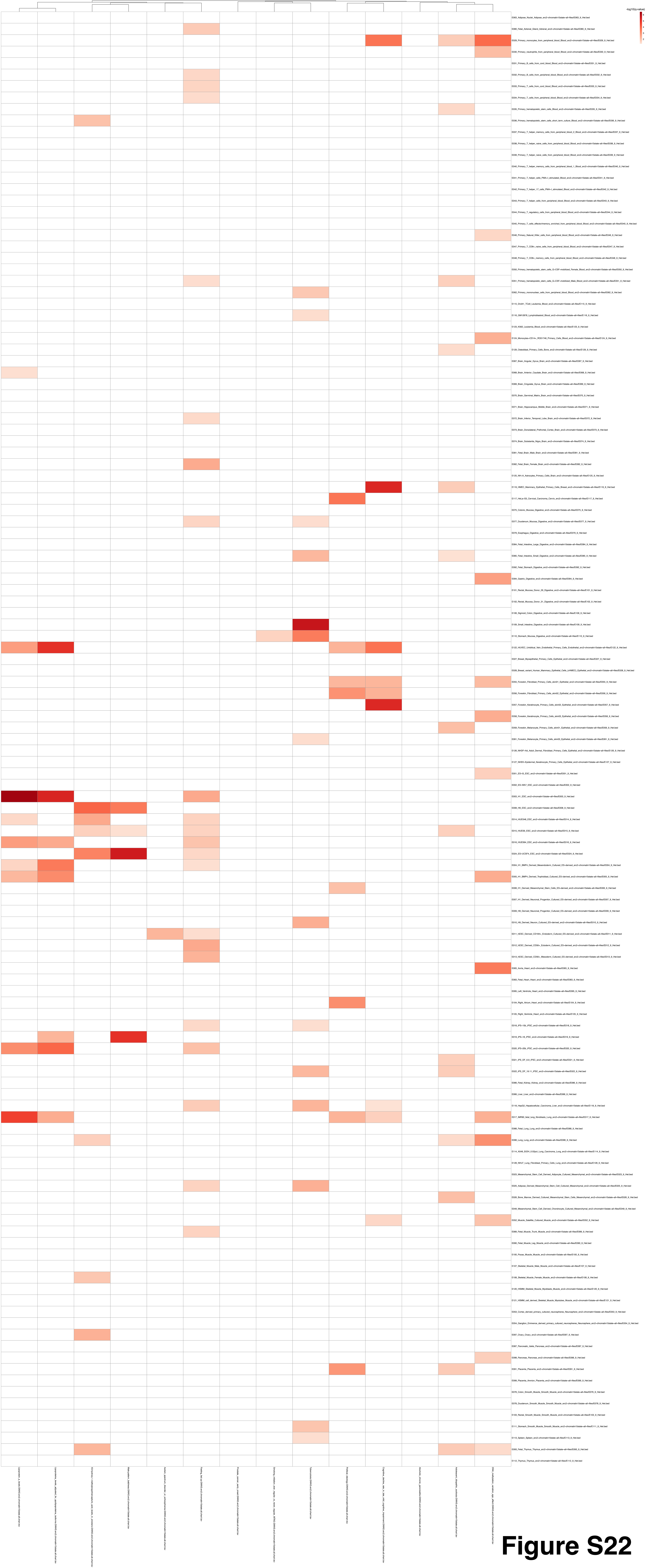

Figure S22

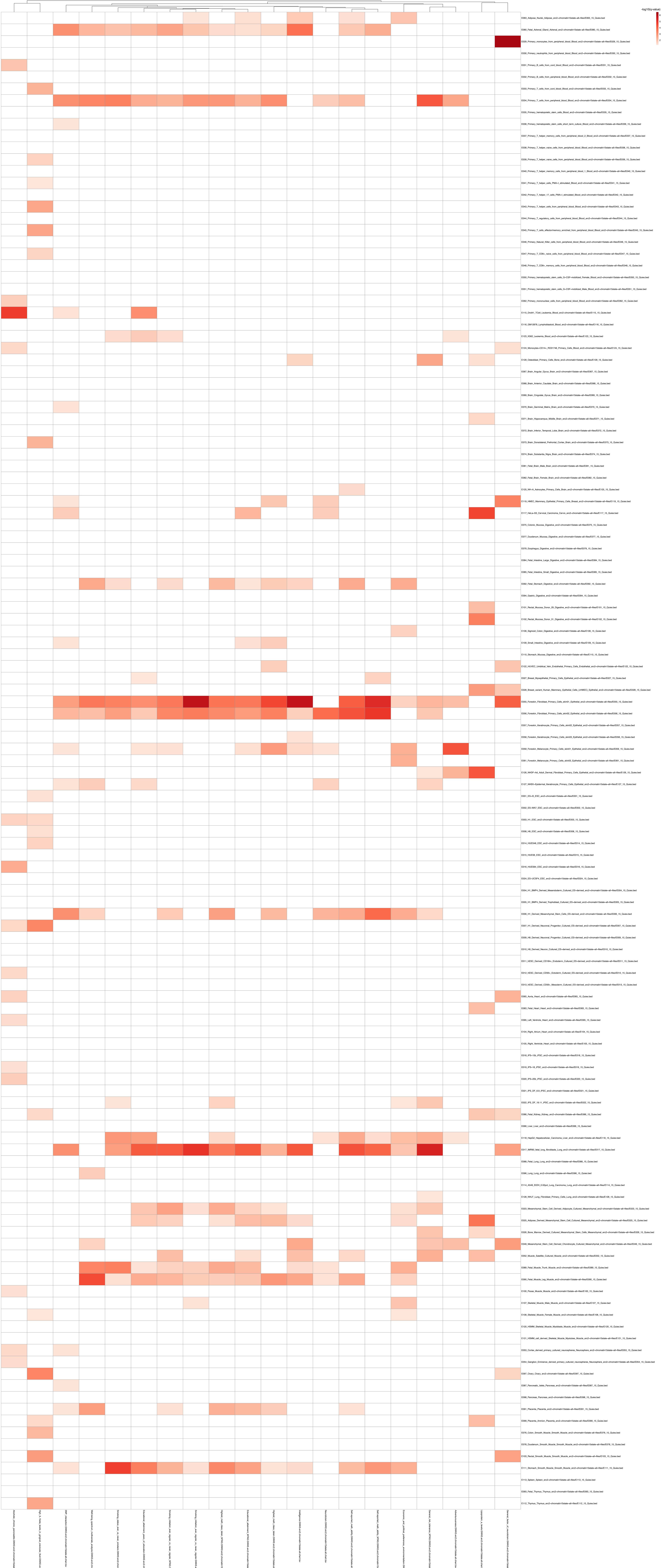

Figure S23

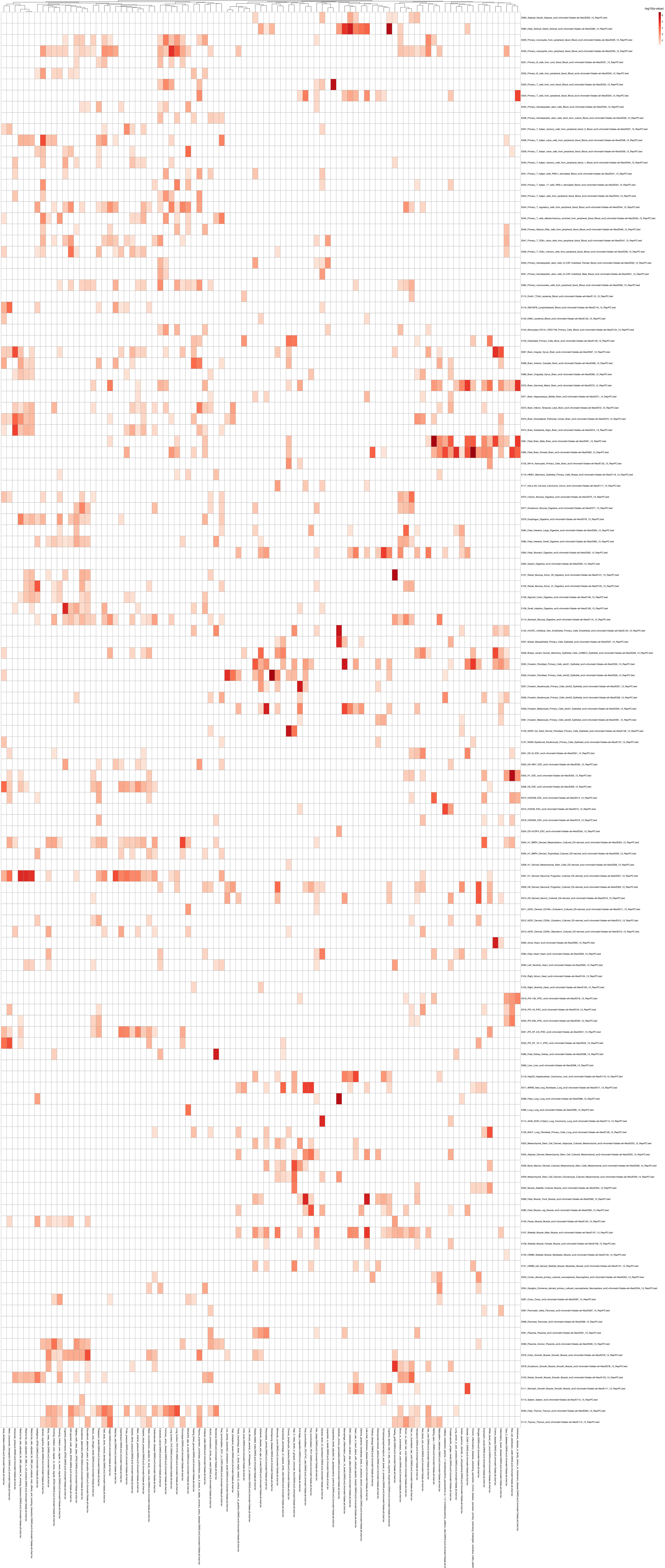

Figure S24

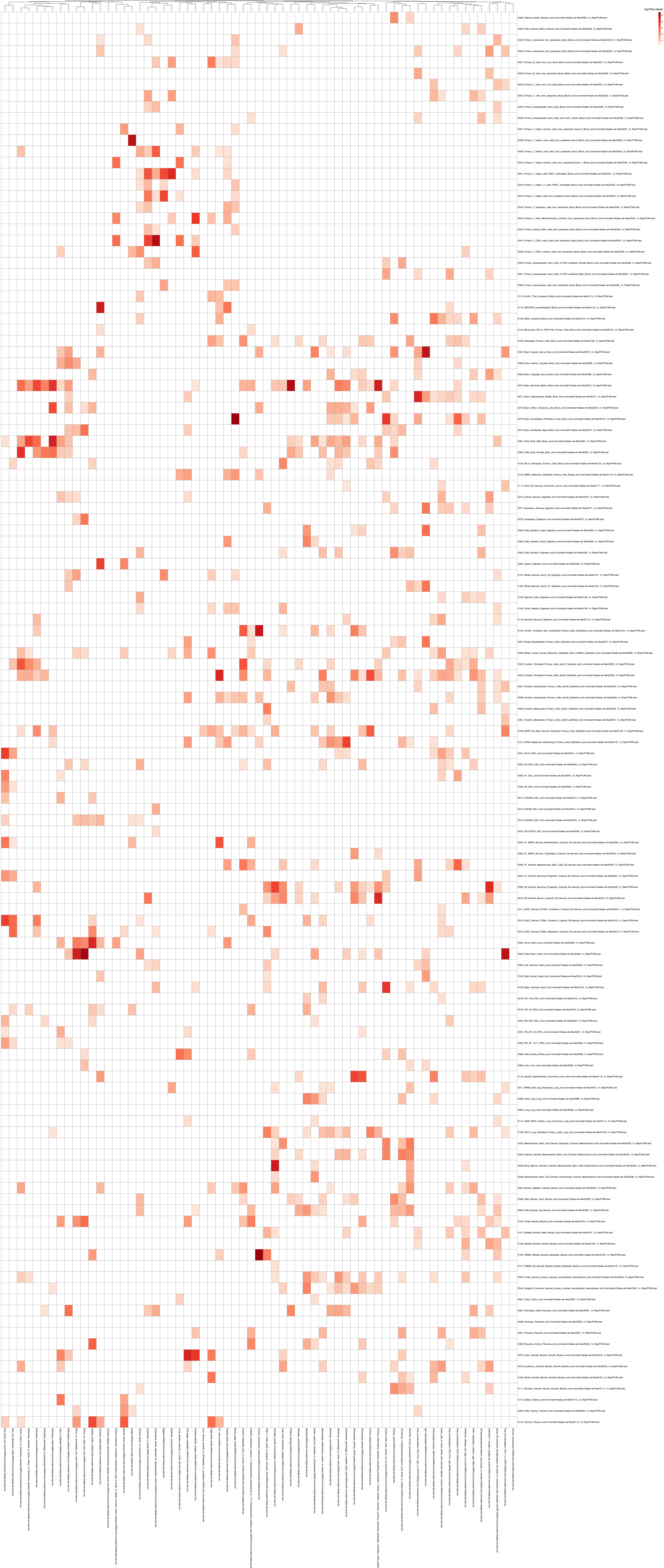

Figure S25

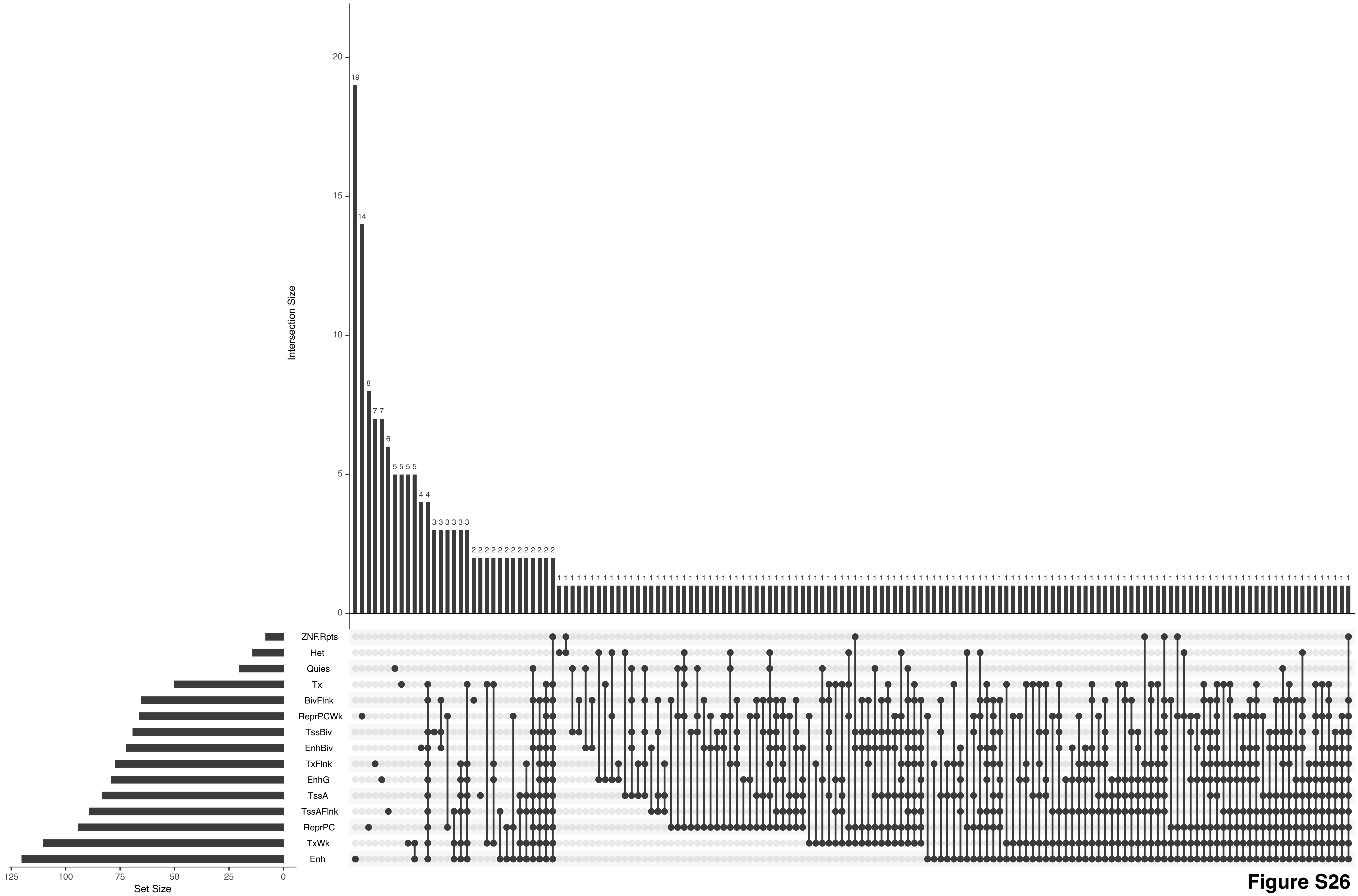
Intersection Size
Set Size
ZNF.Rpts
Het
Quies
Tx
BivFlnk
ReprPCWk
TssBiv
EnhBiv
TxFlnk
EnhG
TssA
TssAFlnk
ReprPC
TxWk
Enh

Figure S26
