# Supplementary material for: Integrative analysis of 3604 GWAS reveals multiple novel cell type-specific regulatory associations
Source: Genome Biol. 2022 Jan 7;23:13. doi: 10.1186/s13059-021-02560-3 (PMC8742386; doi:10.1186/s13059-021-02560-3)
Supplement: Supplementary file 14 — Additional file 14: Review history. [file 13059_2021_2560_MOESM14_ESM.docx]

**Review history**

**First round of review**

**Reviewer 1**

The authors demonstrate the value of their Forge2 web tool for assessing GWAS overlaps within specific epigenomic marks curated across multiple large-scale studies. The tool is potentially useful for GWAS researchers who may want to quickly relate their GWAS significant SNPs to specific cell-type functions. There are also some notable weaknesses to this approach and the presentation of the manuscript and tool:
1) The SNP list that is input to Forge2 is based on a simple overlap of SNPs. How does this approach compare to more sophisticated methods like s-LDSR which uses the fuller distribution of summary statistics
2) The presentation of the manuscript could be considerable reduced and improved. It would further benefit from describing the method and how it compares to other approaches as the first section of the results. The titles of the current results sections are uninformative.
3) The generated figures by Forge2 are large and difficult to read and sort. Can the authors provide functionality for users that would create figures based on significant subsets of the data? The main figures in this manuscript should be improved for presentation. Even if the tool output an R script to produce the figure that someone could work with, it might be helpful in seeing Forge2 results integrated in presentations and papers.
4) For reproducibility, files with GWAS SNPs that were input to Forge2 should be provided with the documentation and there should be some discussion on the minimum number of SNPs required. How does SNP density impact analyses?
5) Is there a process for using unique epigenomics data a user might have? How easy it is to replicate the Forge2 pipeline elsewhere?

**Reviewer 2**

General comments

This paper reports on an update (FORGE2) of the FORGE package. The FORGE package provides a very useful tool for investigators who perform GWAS studies, or want to use them to understand disease etiology or basic biology, to provide a biologically meaningful interpretation of GWAS findings. This is very valuable. And this is a high quality tool that integrates across a lot of complex data that is not easy or practical for investigators to access on their own. And the authors have made it clear in the introduction why FORGE2 is a needed upgrade over the original FORGE. Given the complex material, it is generally fairly well written. But I feel that to make it more useful to the community, careful attention to rewriting so that all terms and concepts are explained for people who are not as familiar with the underlying epigenetic databases as the authors are would help. A limitation in many papers on new bionformatic tools is that they are not written for people less familiar with the epigenetic database than the authors. This makes it harder for people to properly digest and report the output clearly. One attends many terrible talks (and reads discussions sections in good journals) where it is clear that people using many integrative bioinformatics tools do not fully understand the results. Since you have authors capable of explaining these complex data, you are uniquely positioned to write this to better educate the community of people who do GWAS that will use your very useful tool. In general, suggest giving the paper to various scientists who don't use FORGE or similar tools and are not familiar with the epigenetic databases keep rewriting until things are clear. I have many specific comments on how to make the presentation clearer but this is not meant to be exhaustive.

Comments on the abstract

I realize that abstracts have to be short. But you don't let the reader know that there was already FORGE and briefly indicate that this new version adds. I think that this in in the abstract later on but rewriting would enable the reader to have a better sense of how this differs from the previous version and why you would want to use it.

Also, the stats provided about the 3,604 GWAS and 2,057 significant disease-tissue and trait-tissue
Associations are not that helpful. Rather, I would give what is the number of those GWAS where there is one of these two associations. Perhaps one GWAS has multiple. Perhaps the same number were detectable with the original FORGE. And what do you mean by significant disease-tissue and trait-tissue associations? I also don't know what this sentence means "we demonstrate that FORGE2 analysis can establish a relationship between regulatory enrichments". The issues with the abstract are basic writing.

I think that you could also somewhere say why these specific features that you list are important (DNase I hotspots, 5 histone mark categories and 15 hidden Markov model (HMM) chromatin states). For example because SNPs at these locations are more likely to have some functional impact? This probably seems so obvious to you why you would choose these. But this is probably not clear to many readers. This comment applies both to the abstract and the introduction.

Even the title could be improved. It is not obvious what a tissue-specific association is. Association with what? Do you mean that this tool shows you that some GWAS findings suggest that the implicated SNPs might affect gene expression in the target tissue in a way that could influence disease risk. I am not sure what you mean from the title.

Likewise rewrite this key finding - seems like there are missing words - how do you deconvolute an association into a regulatory class? "Redesigned FORGE2 tool can deconvolute previous associations into different regulatory element classes, such as promoters and enhancers". Also earlier in that sentence, it is not clear why the "relationship between regulatory elements" is what most users of the tool to annotate their GWAS findings are wanting to do. That seems like basic biology. Is this just a phrasing issue?

Comments on the introduction:
The introduction does a better job of explaining why you needed up update FORGE to FORGE2. But again rewriting for clarity and context and avoiding terms that are not defined would help. An example "Our tool is designed to analyse GWAS data across both peak-based and modelled epigenomic data tracks". What are "peak-based" tracks as opposed to modelled ones. Peaks of what?

Comments of the Results:
Some examples of where you could do make the writing clearer follow but are not exhaustive.

You say: "we first focused analysis on DNase-seq data. DNase I hypersensitive sites (DHSs) are regions of chromatin sensitive to cleavage by the DNase I enzyme [11]."
I would suggest clarifying to add that DNase-seq can identify the DHSs. Again, you probably assume everyone knows this, but this is not obvious to all people who might want to use FORGE2.
This sentence is dense and obscure: "DNase-seq data was an early epigenetic dataset to be linked to GWAS tissue specific enrichment, analysed both in DHS format [4], and later in DNase I hotspot format…," Does everyone know what the difference or importance of these "formats" is. Just rewrite for clarity.

Another example to clarify "As a result of this analysis, we observe tissue- and cell type-specific enrichments for 293 phenotypes from the GWAS catalog". Can you just add the words to clarify what you mean by "enrichments". How do you calculate the enrichment?

In this sentence can you clarify why "complete-linkage clustering (Euclidean distance)" enables you to find what you did? If this is an important detail, explain it. If not, consider whether you need it here. "Results for these phenotypes were clustered using complete-linkage clustering (Euclidean
distance), revealing….." Later in this paragraph it would help to rewrite to clarify what tissue-associated groups are? "..the existence of tissue-associated groups converging on particular tissues or cell types" Groups of what? Later you imply you mean groups of phenotypes but then to me "tissue associated phenotypes" is unclear. Every phenotype is associated with a tissue. The groups are important to explain because otherwise this result seems weird and obvious and without meaning: "For example, brain was associated with cognitive performance,…"

This sentence is both dense and potentially repetitive making it less clear: "Thus, we demonstrate that FORGE2 can systematically link a wide variety of phenotypes to accessible chromatin elements specific to particular tissues and cell types, confirming previous findings [4], and also revealing previously unreported links between GWAS phenotypes and epigenomic marks for certain cell types and tissues." If I understand what you are saying I suggest rewriting as follows: "Thus, we demonstrate that FORGE2 can systematically link a wide variety of GWAS findings for associations between SNPs and phenotypes to accessible chromatin elements specific to particular tissues and cell types, providing functional confirmation of the GWAS findings…"

It would be useful to include the input GWAS summary dataset that you used for all the example as a supplementary file. That way users could attempt to run that and see if they get the same results as you and then they will know how to interpret results that they might get. For the example analysis, when you say "supplementary table 2 of study" do you mean that you have reproduced the table 2 from the referenced paper? Or is that data already in one of your supp tables? Make that clear. If not already there, you should include the actual file that you input into FORGE2 to run this example as a supplementary file in this paper. Also, in supp tables 1 and 2 what are those Zscores and P values for? Can you add a supp file that defines the columns?

Just a few other examples of where the language is confusing - not exhaustive
In the section on "Relationship between datasets". When you say "we clustered our GWAS results". Do you really mean GWAS results? To me that means the GWAS results you downloaded from the databases. I think you don't mean that but rather the results from running FORGE2 on those.

Methods description
I like the Figure legend for Supp figure 1 as a summary of the Methods. But a few things could be clarified to make it easier to follow. "over 454 samples" confuses me. What 454 samples? What do you mean by "using a similar array background". What array?
In the corresponding text of the methods section, when you say that "FORGE2 takes a set of input SNPs (typically a set of RSIDs) and computes their overlap score (the sum of all overlaps) against epigenomic track data across a range of cell types?
and tissues." can you clarify how you define overlap and how you define this so that you can calculate the score for overlap with a tissue or cell type? This is probably very clear to you but it is not obvious.

Outputs section
You say that various pdfs come out of running FORGE2. You ran the example using an MPN GWAS dataset. I don't know how many pdfs come out of FORGE 2. Could you possibly make a supplementary file with all of those with annotation regarding how to interpret them? Can you give a couple of examples of key pdfs that come out of FORGE2 with some annotation of how to interpret them? This would be very useful. I assume it is more than just Figure 6 a and b.

False positive rate testing
You indicate that the original FORGE used Bonferroni but this one uses FDR<0.05. Can you give a reason for the change? Did you get feedback on this point?

**Authors’ response to reviewers**

Reviewer #1:

The authors demonstrate the value of their Forge2 web tool for assessing GWAS overlaps within specific epigenomic marks curated across multiple large-scale studies. The tool is potentially useful for GWAS researchers who may want to quickly relate their GWAS significant SNPs to specific cell-type functions. There are also some notable weaknesses to this approach and the presentation of the manuscript and tool:

**1) The SNP list that is input to Forge2 is based on a simple overlap of SNPs. How does this approach compare to more sophisticated methods like s-LDSR which uses the fuller distribution of summary statistics?**

**Response:** Thank you for the opportunity to clarify this. We would like to highlight that FORGE2 and s-LDSR are different methods that are geared toward answering different questions. Both methods use summary results from GWAS. Partitioned LDSR estimates the SNP heritability that can be attributed to different annotation components. FORGE2 does not estimate heritability, but rather provides tissue-specific enrichment statistics and assesses annotations for specific GWAS loci that have a high degree of association with a given phenotype. FORGE2 does not only compute overlaps but also systematically assesses tissue- and cell type-specific enrichment using a statistical model that accounts for multidimensional genomic biases including several different pools with millions of background SNPs. FORGE2 generates thousands of specialized background SNP sets for each input set, adjusting for minor allele frequency, transcription start site, and GC content. Unlike LDSR, which is based on the entire genome, FORGE2 is focused on the statistical analysis of SNP sets and annotating individual variants, which can be further analyzed via laboratory methods (e.g. massively parallel reporter assay/CRISPR experimentation). Therefore, these tools are complementary to one another and provide added utility to the current GWAS analysis toolbox.

FORGE2 has some advantages over LDSR. Partitioned LDSR requires a GWAS dataset with a relatively large sample size (e.g., N>10,000) and/or large heritability, and the disease or trait must be polygenic. Thus, it is not useful for smaller studies or datasets with limited polygenicity. In addition, it requires an LD reference population that matches the dataset, which does not allow it to be used with multi-ancestry or admixed populations or other datasets without a similar LD reference panel. In contrast, FORGE2 does not have restrictions on sample size or population ancestry (as it utilizes a diverse reference population). It typically runs in < 1 minute, is available as a web tool, and does not require a formatted dataset or external LD reference panel, which can be cumbersome to format. It allows for widespread use across a broad range of GWAS data from rare diseases to specialized populations. For example, we were able to use FORGE2 to analyze GWAS results from four lymphoid malignancies (i.e., chronic lymphocytic leukemia, diffuse large B-cell lymphoma (DLBCL), marginal zone lymphoma (MZL), and follicular lymphoma) and show enrichment for specific immune cell types:

However, the datasets were too small to yield stable results using partitioned or cross-trait LDSR. FORGE2 can analyze data and provide insight across many different traits/diseases, including GWAS of rare diseases or unique populations, where large datasets are not feasible.

**2) The presentation of the manuscript could be considerable reduced and improved. It would further benefit from describing the method and how it compares to other approaches as the first section of the results. The titles of the current results sections are uninformative.**

**Response:** Thank you for the suggestion. We have shortened and revised the text to make it easier to read (please see also reviewer 2 response). We have added text describing the method and how it complements other methods. Specifically, we have added the following text:

“FORGE2 has a specific and unique role in the field of GWAS analysis methods. It performs multidimensional epigenomic data analysis across hundreds of samples and chromatin data types (including DNase I hotspots, histone mark broadPeaks and HMM chromatin states), utilizing background SNP sets comprising thousands of selected variants adjusted for multiple genomic biases to robustly test for enrichment, and optimized in a fast and easy to use web tool. Unlike LD score regression [29] [30] FORGE2 does not rely on a specific reference population or require a large GWAS sample size, and it can be used to evaluate results from a broad range of GWAS, including GWAS from multi-ancestry, admixed or unique populations, as well as GWAS on both common and rare diseases. FORGE2 is focused on the statistical analysis of SNP sets, as well as annotating individual variants. FORGE2 rapid analysis of regulatory associations can aid further investigation of variants via laboratory methods (e.g. massively parallel reporter assays/CRISPR experimentation). As such, it possesses unique characteristics and targets a niche different from that of other current tools [10] [31] [29] [30].”

We have revised the results section titles to make them more informative.

**3) The generated figures by Forge2 are large and difficult to read and sort. Can the authors provide functionality for users that would create figures based on significant subsets of the data? The main figures in this manuscript should be improved for presentation. Even if the tool output an R script to produce the figure that someone could work with, it might be helpful in seeing Forge2 results integrated in presentations and papers.**

**Response:** We have provided a functionality to plot subsets of FORGE2 figures by incorporating R code for figure generation in the output of FORGE2. An example of this R code is shown at <https://forge2.altiusinstitute.org/files/0x5DB893F6D51A11EB9CCA935FB5A874F8/Unnamed.GWAS.erc2-H3K4me1.chart.R> for the PDF and <https://forge2.altiusinstitute.org/files/0x5DB893F6D51A11EB9CCA935FB5A874F8/Unnamed.GWAS.erc2-H3K4me1.dChart.R> for the interactive D3 chart. Selecting subsets of the data is as straightforward as changing the tsv file that is the input to the plotting program, or indicating subsets to be plotted within the R script. Some example code to select a subset of the data is available at <https://github.com/charlesbreeze/FORGE2/blob/forge2.v1.0/bin/plot.subsets/plot.subsets.txt>.

To address the problem of viewing FORGE2 results shown in this paper we have provided zoomable, high resolution versions for all the main figures and all supplementary figures at <https://www.easyzoom.com/>.

All figures are included in easyzoom in the following folder: <https://www.easyzoom.com/albumaccess/3de0ae35c00b41189f63d13635bdb8d9>.

All zoomable figure links have been added to the figure legends and the link for the easyzoom folder has been added to the manuscript:

“Zoomable versions of all figures were uploaded to easyzoom (<https://www.easyzoom.com>) and are available in the following easyzoom folder:

<https://www.easyzoom.com/albumaccess/3de0ae35c00b41189f63d13635bdb8d9>. “

While Figure 6 in this manuscript is an example of FORGE2 standard output, other main Figures such as 1, 2, 4 and 5 are not the standard output of FORGE2, but rather clustered heatmaps of FORGE2 tabular results. The FORGE2 standard output figure is designed for multiple tissues and one GWAS.

Following the reviewer’s recommendation, we have created code to generate subplots of main figures 1, 2, 4 and 5. We have provided the code in the following github directory (plot.filesets.sh and heatmap.R): <https://github.com/charlesbreeze/FORGE2/tree/forge2.v1.0/bin/plot.heatmaps>

We have added the link to code for subsetting FORGE2 plots to the manuscript:

“Example code for creating FORGE2 plot subsets can be found on GitHub at: <https://github.com/charlesbreeze/FORGE2/blob/forge2.v1.0/bin/plot.subsets/plot.subsets.txt>.”

**4) For reproducibility, files with GWAS SNPs that were input to Forge2 should be provided with the documentation and there should be some discussion on the minimum number of SNPs required. How does SNP density impact analyses?**

**Response:** All input SNP lists, output analyses and figures have been uploaded to the FORGE2 Google Drive directory and are accessible from the following link: <https://drive.google.com/drive/folders/10GYaZQj_dF8FVj8LzMMBTlf1MwNBIIpU?usp=sharing>

FORGE2 requires a minimum of five SNPs to run; and a higher number of SNPs will result in improved power for analysis. The minimum SNP level was set based on assessment of false positive rates by simulation as described in the work by Dunham et al. (https://www.biorxiv.org/content/10.1101/013045v1) and shown in the figure below:

For the GWAS analysis in this manuscript, we required a minimum of 5 SNPs. We have added information regarding the minimum number of SNPs used in our analysis to the manuscript:

“For this analysis, we required each phenotype (or GWAS) to have a minimum of 5 SNPs.”

The reviewer raises a good question regarding the potential impact of SNP density on our results. It is theoretically possible that local SNP density might boost tissue-specific enrichment signals. To account for the potential impact of SNP density, FORGE2 includes LD filtering at r^2^ > 0.8 as default.

To further explore how SNP density and LD might impact analysis results, we conducted FORGE2 analysis across 5 tissues/cell types representative of key vital functions (heart, kidney, HSCs, lung and thymus) and 15 traits/diseases (3 representative traits for each tissue/cell type), using more stringent LD pruning.

In our first analysis, we pruned SNPs at an increased LD stringency of r^2^ > 0.1. Despite the reduced number of SNPs, FORGE2 showed the same tissue-specific enrichment results for all 15 traits as we observed previously using an r2>0.8 (see table below). This indicates that, at least for these traits, SNP pruning with r^2^ > 0.1 does not significantly affect the results.

We conducted a second more stringent analysis, pruning down to one SNP per chromosome, which effectively eliminates any effects of LD or SNP density. Again, FORGE2 showed the same tissue-specific enrichment results for all 15 traits, indicating that, for these traits, SNP pruning at the chromosomal level does not affect results.

| **Tissue/cell type** | **Phenotype** | **Significant tissue/cell type after pruning SNPs with R^2^>0.8** | **Significant tissue/cell type after pruning SNPs with r^2^>0.1** | **Significant tissue/cell type after limiting to 1 SNP per chromosome** |
| --- | --- | --- | --- | --- |
| Kidney | Blood_urea_nitrogen_levels | Kidney | Kidney | Kidney |
| Kidney | Renal_function.related_traits_BUN | Kidney | Kidney | Kidney |
| Kidney | Urate_levels | Kidney | Kidney | Kidney |
|  |  |  |  |  |
| Heart | Resting_heart_rate | Heart | Heart | Heart |
| Heart | Atrial_fibrillation | Heart | Heart | Heart |
| Heart | PR_interval | Heart | Heart | Heart |
|  |  |  |  |  |
| Immune cells | Asthma_childhood_onset | CD4 | CD4 | CD4 |
| Immune cells | Takayasu_arteritis | CD19 | CD19 | CD19 |
| Immune cells | Juvenile_idiopathic_arthritis | Thymus | Thymus | Thymus |
|  |  |  |  |  |
| HSCs | Mean_platelet_volume | HSCs | HSCs | HSCs |
| HSCs | Platelet_distribution_width | HSCs | HSCs | HSCs |
| HSCs | Neutrophil_percentage_of_granulocytes | HSCs | HSCs | HSCs |
|  |  |  |  |  |
| Lung | Pulmonary_function | Lung | Lung | Lung |
| Lung | Medication_use_drugs_used_in_diabetes | Lung | Lung | Lung |
| Lung | Pulmonary_function_smoking_interaction | Lung | Lung | Lung |

Of course, it is theoretically possible that a reduced number of SNPs (as a result of pruning) might, for other traits, result in a reduced FORGE2 signal, as there is less information included in the input set. Additionally, the FORGE2 web tool requires a minimum of 5 SNPs, so running an analysis using a pruned SNP list below this level is not possible on the web tool. Nevertheless, the results, for these 15 traits show a robustness to SNP density in the analysis and indicate that SNP density is likely not a key determining factor in FORGE2 analysis for the diseases and traits analyzed here.

We have clarified the LD filtering applied in our study in the text as follows: “To account for SNP density, we used the default LD filter of r^2^ >= 0.8 for this analysis.”

**5) Is there a process for using unique epigenomics data a user might have? How easy it is to replicate the Forge2 pipeline elsewhere?**

**Response:** It is currently not possible for the user to upload epigenomics data to the online version of FORGE2. However, we have a stand-alone version of FORGE2 that may be used for facilitated and open-sourced database construction. We have placed all the code for generating the FORGE2/eFORGE-type databases from scratch openly on GitHub (see <https://github.com/charlesbreeze/eFORGE/blob/master/database/README.txt> and examples in the same directory). We have added this link to the text:

“FORGE2 code has been tested on Mac OSX 10.12.6 and Linux Centos 7. Source code is available at <https://github.com/charlesbreeze/FORGE2>. Information for generating FORGE2/eFORGE-type databases from scratch can be found here: <https://github.com/charlesbreeze/eFORGE/blob/master/database/README.txt>.”

To facilitate data addition, we have changed the design of the SQLite database to introduce bitstring tables in a modular fashion, and we have also provided indications on FORGE2/eFORGE GitHub to produce bitstring tables from raw data.

To address this point in this manuscript, we have added the following text: “The addition of new epigenomic track data to the standalone FORGE2 database can be performed using bedtools/bedops and similar openly available code to previous methods [35].“

FORGE2 has been installed on several different systems including mac OSX, macOS, CentOS Linux. Clear installation instructions are provided on GitHub and, once the database is downloaded and perl is set up with the appropriate modules, the most recent code (as well as previous versions) can be simply downloaded from GitHub and run on Linux and mac OSX /macOS. A recent install was performed using GitHub and took around 10 minutes.

===

Please consider the following questions:

- Are the methods appropriate to the aims of the study, are they well described, and are necessary controls included? If not, please specify what is required.

- Are the conclusions adequately supported by the data shown? If not, please explain

- Are sufficient details provided to allow replication and comparison with related analyses that may have been performed? If not, please specify what is required.

- Does the work represent a significant advance over previously published studies?

- Is the paper of broad interest to others in the field, or of outstanding interest to a broad audience of biologists?

Reviewer #2: ===

This box is for your report. All comments included here will be passed to the authors.

===

Please consider the following questions:

- Are the methods appropriate to the aims of the study, are they well described, and are necessary controls included? If not, please specify what is required.

- Are the conclusions adequately supported by the data shown? If not, please explain

- Are sufficient details provided to allow replication and comparison with related analyses that may have been performed? If not, please specify what is required.

- Does the work represent a significant advance over previously published studies?

- Is the paper of broad interest to others in the field, or of outstanding interest to a broad audience of biologists?

*General comments*

*This paper reports on an update (FORGE2) of the FORGE package. The FORGE package provides a very useful tool for investigators who perform GWAS studies, or want to use them to understand disease etiology or basic biology, to provide a biologically meaningful interpretation of GWAS findings. This is very valuable. And this is a high quality tool that integrates across a lot of complex data that is not easy or practical for investigators to access on their own. And the authors have made it clear in the introduction why FORGE2 is a needed upgrade over the original FORGE. Given the complex material, it is generally fairly well written. But I feel that to make it more useful to the community, careful attention to rewriting so that all terms and concepts are explained for people who are not as familiar with the underlying epigenetic databases as the authors are would help. A limitation in many papers on new bionformatic tools is that they are not written for people less familiar with the epigenetic database than the authors. This makes it harder for people to properly digest and report the output clearly. One attends many terrible talks (and reads discussions sections in good journals) where it is clear that people using many integrative bioinformatics tools do not fully understand the results. Since you have authors capable of explaining these complex data, you are uniquely positioned to write this to better educate the community of people who do GWAS that will use your very useful tool. In general, suggest giving the paper to various scientists who don't use FORGE or similar tools and are not familiar with the epigenetic databases keep rewriting until things are clear. I have many specific comments on how to make the presentation clearer but this is not meant to be exhaustive.*

**Response:** Thank you for all the comments. We have done extensive edits to the paper to address the issues raised by the reviewers and make the manuscript more readable to the average researcher. We believe that these changes have improved our manuscript.

*Comments on the abstract*

*Let the reader know that there was already FORGE and briefly indicate that this new version adds. I think that this in in the abstract later on but rewriting would enable the reader to have a better sense of how this differs from the previous version and why you would want to use it.*

**Response:** Thank you for the suggestion. We have included this information in the following paragraph:

"To gain insight into potential functional mechanisms underlying GWAS associations, we developed FORGE2 (<https://forge2.altiusinstitute.org/>), which is an updated version of the FORGE web tool. FORGE2 uses an expanded atlas of cell type-specific regulatory element annotations, including DNase I hotspots, five histone mark categories and 15 hidden Markov model (HMM) chromatin states, to identify tissue- and cell type-specific signals. An analysis of 3,604 GWAS from the NHGRI-EBI GWAS catalogue yielded at least one significant disease/trait-tissue association for 2,057 GWAS”

*Also, the stats provided about the 3,604 GWAS and 2,057 significant disease-tissue and trait-tissue associations are not that helpful. Rather, I would give what is the number of those GWAS where there is one of these two associations. Perhaps one GWAS has multiple. Perhaps the same number were detectable with the original FORGE. And what do you mean by significant disease-tissue and trait-tissue associations?*

**Response:** The reviewer raises several good points. When we performed FORGE2 analysis against a given trait or disease, we recorded the number of GWAS that presented at least one significant tissue-specific enrichment across all tissues. We have clarified this in the abstract as follows:

“An analysis of 3,604 GWAS from the NHGRI-EBI GWAS catalogue yielded at least one significant disease/trait-tissue association for 2,057 GWAS, including > 400 associations specific to epigenomic marks in immune tissues and cell types, > 30 associations specific to heart tissue, and > 60 associations specific to brain tissue, highlighting the key potential of tissue- and cell type-specific regulatory elements.”

Compared to FORGE for the same GWAS catalogue data, FORGE2 detects a higher number of associations (FORGE2 detects 2,057, FORGE detects 413 associations). We have added this comparison to the text:

“Of the 3,604 GWAS evaluated, we identified at least one tissue- or cell-specific association for 2,057 GWAS, detecting more associations than FORGE, which can only detect 413 associations for the same data, and highlighting the importance of tissue-specific regulatory elements.”

*I also don't know what this sentence means "we demonstrate that FORGE2 analysis can establish a relationship between regulatory enrichments". The issues with the abstract are basic writing.*

**Response:** We have revised the abstract and clarified this statement as follows:

“Importantly, we demonstrate that FORGE2 analysis can separate previously observed accessible chromatin enrichments into different chromatin states, such as enhancers or active transcription start sites, providing a greater understanding of underlying regulatory mechanisms.”

FORGE2 analysis across multiple epigenomic marks showed evidence for enrichments in some marks indicating general regulatory activity (e.g. accessible chromatin, a mark covering many different types of regulatory regions) and more specific regulatory activity (e.g. active enhancer chromatin state regions) present for the same tissue. In some cases, tissue-specific enrichments in marks indicating general regulatory activity were also present in the same tissue for two different marks indicating specific regulatory activity (e.g. active promoters and enhancer chromatin states), with different sets of SNPs underlying each one of these two categories. Therefore, we were able to understand what regulatory elements were underlying the previous "accessible chromatin" enrichment, thus separating (deconvoluting) one enrichment in a given tissue into enrichments for two classes of elements, active promoters and enhancers. This way, and by analyzing the exact SNPs involved, we could understand the relationship between one tissue-specific enrichment covering all regulatory elements (accessible chromatin) and other tissue-specific enrichments covering specific regulatory elements (active promoter and enhancer chromatin states).

We have added a description of this in the text as:

“We also discovered that for some phenotypes, tissue-specific enrichments for marks indicating general regulatory activity (e.g. accessible chromatin) were also present in the same tissue for two or more different marks indicating specific regulatory activity (e.g. active promoters and enhancer chromatin states), with different sets of SNPs underlying each one of these two categories. By understanding which regulatory elements were underlying the previous "accessible chromatin" enrichment and separating these enrichments into different classes of regulatory elements, we can gain a greater understanding of the underlying biology of the GWAS SNPs.”

*I think that you could also somewhere say why these specific features that you list are important (DNase I hotspots, 5 histone mark categories and 15 hidden Markov model (HMM) chromatin states). For example because SNPs at these locations are more likely to have some functional impact? This probably seems so obvious to you why you would choose these. But this is probably not clear to many readers. This comment applies both to the abstract and the introduction.*

**Response**: We have modified the abstract to include a reference to the regulatory element annotations:

"FORGE2 uses an expanded atlas of cell type-specific regulatory element annotations, including DNase I hotspots, five histone mark categories and 15 hidden Markov model (HMM) chromatin states, to identify tissue- and cell type-specific signals.”

We have added text in the introduction to clarify the importance of evaluating regulatory regions when analyzing data for DNase-seq data:

"One way to aid the interpretation of GWAS results is by integrating large-scale epigenomic mapping data generated by projects like ENCODE, Roadmap Epigenomics, and BLUEPRINT [5][6][7]. In 2012, ENCODE scientists [4] demonstrated that by integrating DNase-seq data from large-scale epigenomic mapping projects with disease-associated GWAS variants one can systematically categorize and localize key disease-associated cell types, tissues, and regulatory regions.”

"Over the past few years, there has been an explosion in epigenomic data tracks, including ChIP-seq, DNase-seq and hidden Markov model (HMM) chromatin state tracks. When integrated with GWAS results, this new generation of data can offer further insight into the functional mechanisms of genetic loci discovered from GWAS and help discriminate SNPs responsible for a specific phenotype from other nearby genetic variants. However, this new epigenomic data can be difficult access and integrate into GWAS analyses. There is a need to provide an updated, user-friendly and comprehensive framework for the categorisation, analysis and prioritisation of GWAS loci based on regulatory elements, in order to facilitate the identification of associated regulatory pathways, drug targets, cis-regulatory elements and cell types/tissues. To address this need, we developed FORGE2, a new statistical framework and analysis tool, which expands upon FORGE and incorporates new epigenomic data tracks, including DNase I hotspots, histone mark broadPeaks and HMM chromatin state tracks. The expanded atlas of cell type-specific regulatory element annotations in FORGE2 provides additional information that can be used to further refine and understand identified loci from GWAS.”

We have also added text explaining DNase I hypersensitivity sites, histone modifications, and chromatin states and their importance:

"DNase-seq data is used to map DNase I hypersensitive sites (DHSs), which are regions of chromatin sensitive to cleavage by the DNase I enzyme [11]. DHSs are classic markers of regions of regulatory DNA, covering many types of cis-regulatory elements (CREs) including promoters, enhancers, silencers, insulators and locus control regions [12].”

"DNase I hotspot enrichments may be driven by variants located in different classes of cis-regulatory element (CRE), such as promoters and enhancers, all of which are in accessible chromatin. Particular histone modifications (e.g. H3K4me3) are closely associated with specific CRE classes (such as promoters). Posttranslational modifications of histone proteins are known to influence processes as diverse as gene regulation, DNA repair and chromosome condensation [13]. Specific histone modifications are enriched for certain types of genomic annotations such as enhancers (H3K4me1), promoters (H3K4me3), transcribed regions (H3K36me3), heterochromatin regions (H3K9me3), and polycomb-repressed regions (H3K27me3) [6]. Enrichment of GWAS loci in these regions could provide additional information regarding underlying mechanisms.”

"Although examination of histone marks can yield information about GWAS findings, additional information can be gained by evaluating the combination of multiple marks, informing segmentations of chromatin known as chromatin states. These chromatin states can further refine classes of genomic or regulatory elements, such as promoters, enhancers, repressed regions, as well novel classes or subclasses of elements [14]."

*Even the title could be improved. It is not obvious what a tissue-specific association is. Association with what? Do you mean that this tool shows you that some GWAS findings suggest that the implicated SNPs might affect gene expression in the target tissue in a way that could influence disease risk. I am not sure what you mean from the title.*

**Response:** These are tissue- and cell type-specific regulatory associations, affecting tissue- and cell type-specific regulatory regions.

To make this clear, we have changed the title to:" Integrative analysis across 3,604 GWAS reveals multiple novel cell type-specific regulatory associations "

*Likewise rewrite this key finding - seems like there are missing words - how do you deconvolute an association into a regulatory class? "Redesigned FORGE2 tool can deconvolute previous associations into different regulatory element classes, such as promoters and enhancers". Also earlier in that sentence, it is not clear why the "relationship between regulatory elements" is what most users of the tool to annotate their GWAS findings are wanting to do. That seems like basic biology. Is this just a phrasing issue?*

**Response:** In our FORGE2 analysis of GWAS results, we discovered cell type- and tissue-specific enrichment for some marks indicating general regulatory activity (e.g. accessible chromatin, a mark covering many different types of regulatory regions) and for more specific regulatory activity (e.g. active enhancer chromatin state regions) in the same tissue. In some cases, tissue-specific enrichments for marks indicating general regulatory activity were also present in the same tissue for two different marks indicating specific regulatory activity (e.g. active promoters and enhancer chromatin states), with different sets of SNPs underlying each one of these two categories. Therefore, we were able to understand what regulatory elements were underlying the previous "accessible chromatin" enrichment, thus separating (deconvoluting) one enrichment in a given tissue into enrichments for two classes of elements, active promoters and enhancers. With this knowledge and by analyzing the exact SNPs involved, we can gain a greater understanding of the relationship between one tissue-specific enrichment covering all regulatory elements (accessible chromatin) and other tissue-specific enrichments covering specific regulatory elements (active promoter and enhancer chromatin states) and the underlying biology of the GWAS SNPs.

Some phenotypes tend to be associated with enhancers, and others seem to be associated with other types of regulatory regions (e.g. regions of active transcription). Although we may not understand why GWAS SNPs tend to localize to specific types of regulatory regions for some phenotypes, it provides information about the underlying biological architecture of genetic susceptibility to these diseases and traits. This is presumably of interest to researchers who are seeking to understand their own GWAS results, as it can give them information on the exact types of tissue-specific regulatory elements impacted by genome-wide significant SNPs from their study.

We have clarified the key finding as follows:

“Redesigned FORGE2 tool can separate previous associations into specific regulatory element classes, such as promoters and enhancers“

We have also added following text to the discussion, which provides more explanation:

“We also discovered that for some phenotypes, tissue-specific enrichments for marks indicating general regulatory activity (e.g. accessible chromatin) were also present in the same tissue for two or more different marks indicating specific regulatory activity (e.g. active promoters and enhancer chromatin states), with different sets of SNPs underlying each one of these two categories. By understanding which regulatory elements were underlying the previous "accessible chromatin" enrichment and separating these enrichments into different classes of regulatory elements, we can gain a greater understanding of the underlying biology of the GWAS SNPs. We also observed that some phenotypes tend to be associated with specific regulatory regions (e.g., enhancers), and others seem to be associated with multiple types of regulatory marks. Although we may not understand why GWAS SNPs tend to localize to specific types of regulatory regions for some phenotypes, these results are a step forward and provide information about the underlying biological architecture of genetic susceptibility to these diseases and traits.”

*Comments on the introduction:*

*The introduction does a better job of explaining why you needed up update FORGE to FORGE2. But again rewriting for clarity and context and avoiding terms that are not defined would help. An example "Our tool is designed to analyse GWAS data across both peak-based and modelled epigenomic data tracks". What are "peak-based" tracks as opposed to modelled ones. Peaks of what?*

**Response:** Technologies like chromatin immunoprecipitation followed by sequencing (ChIP-seq) yield genomic read information that can be mapped to specific genomic regions. These mapped reads tend to accumulate in and around regulatory regions, creating peaks, which are clusters of reads identified by a peak calling software (such as MACS). Peaks of mapped reads (DNase I hypersensitive sites) may also be obtained from similar technologies such as DNase-seq, where reads may also be clustered into larger regions called DNase hotspots.

The whole set of read-based peaks for each sample constitutes a track, and when analyzing multiple samples, multiple tracks are present. These (peak-based) tracks are based on mapping of direct experimental data (genomic reads). Certain modelling approaches (such as Hidden Markov Models) may be applied to large sets of diverse data obtained from different experiments. These experiments typically involve ChIP-seq data for histone modifications such as H3K4me1, H3K4me3, among others, along with DNase-seq data, DNA methylation data, gene expression data, etc. These diverse datasets are then input into a model that can categorize genomic regions into chromatin state segmentations such as active enhancers, active promoters, etc. This is what we refer to as modelled tracks. They are different than peak-based tracks as they typically receive input from many prior epigenomic tracks or annotations and are strongly dependent on the model.

However, given that all these distinctions and classifications might be confusing, we have removed a mention of this in the text and have simplified the introduction section.

*Comments of the Results:*

*Some examples of where you could do make the writing clearer follow but are not exhaustive.*

*You say: "we first focused analysis on DNase-seq data. DNase I hypersensitive sites (DHSs) are regions of chromatin sensitive to cleavage by the DNase I enzyme [11]."*

*I would suggest clarifying to add that DNase-seq can identify the DHSs. Again, you probably assume everyone knows this, but this is not obvious to all people who might want to use FORGE2.*

**Response:** We have clarified this sentence as follows:

"To gain understanding of the relationship between GWAS findings and regulatory regions, we first focused on DNase-seq data. DNase-seq data is used to map DNase I hypersensitive sites (DHSs), which are regions of chromatin sensitive to cleavage by the DNase I enzyme [11]. ”

*This sentence is dense and obscure: "DNase-seq data was an early epigenetic dataset to be linked to GWAS tissue specific enrichment, analysed both in DHS format [4], and later in DNase I hotspot format…," Does everyone know what the difference or importance of these "formats" is. Just rewrite for clarity.*

**Response:** We have rewritten this section as follows:

"DNase-seq data is used to map DNase I hypersensitive sites (DHSs), which are regions of chromatin sensitive to cleavage by the DNase I enzyme [11]. DHSs are classic markers of regions of regulatory DNA, covering many types of cis-regulatory elements (CREs) including promoters, enhancers, silencers, insulators and locus control regions [12]. We used DNase-seq data from ENCODE, BLUEPRINT and Roadmap Epigenomics (2012 and 2015 data releases) to identify DNase I hotspots, which are similar to DHSs but encompass broader regions of DNase hypersensitivity [10].”

*Another example to clarify "As a result of this analysis, we observe tissue- and cell type-specific enrichments for 293 phenotypes from the GWAS catalog". Can you just add the words to clarify what you mean by "enrichments". How do you calculate the enrichment?*

**Response:** An enrichment or overrepresentation constitutes a significantly higher overlap between regulatory regions and the input set of SNPs compared to the FORGE2 background sets of SNPs, after accounting for multiple testing. These background sets of SNPs have been adjusted for transcription start site (TSS) distance, GC content and minor allele frequency (MAF). Each set possesses the same number of SNPs as the input set. Analyses in this paper all include 1000 background sets for each input set. Statistical enrichment is the same as statistical overrepresentation.

We have clarified this sentence as follows:

"We observed significant tissue- and cell type-specific enrichment for DNase I hotspots for 293 phenotypes from the GWAS catalogue (q-value <0.01) (**Figure 1, Supplementary Figures 2-4, Supplementary Tables 1-4**).”

We have included details regarding the enrichment analysis in the “Functional overlap analysis” in the Methods section:

“To test for enrichment (higher overlap than expected), FORGE2 first computes an overlap score for a SNP set (the sum of all overlaps of that set) against epigenomic track data across a range of cell types and tissues. For each epigenomic track sample, the overlap score of the input set is then compared to the overlap scores of 1000 background SNP sets that FORGE2 generates. SNPs from these 1000 background sets are selected for similarity to the input SNP set, via matching decile bins for minor allele frequency (MAF), distance to transcription start site (TSS) and GC content. A binomial test is used to compare the overlap score of the input SNP set with the overlap scores of the 1000 background sets. This is repeated across multiple cell/tissue samples, until all samples have been analyzed. The p-values from the binomial tests for all cell/tissue samples tested are then adjusted for multiple testing via the Benjamini-Hochberg (BH) method [39], generating q-values as a result. BH is applied at a sample level, including all samples and p-values. Enrichments at a q-value<0.01 are considered significant. Alongside q-values, corresponding uncorrected p-values and Z-scores are also provided by FORGE2.”

*In this sentence can you clarify why "complete-linkage clustering (Euclidean distance)" enables you to find what you did? If this is an important detail, explain it. If not, consider whether you need it here. "Results for these phenotypes were clustered using complete-linkage clustering (Euclidean*

*distance), revealing….."*

**Response:** We agree that this is not a sufficiently important detail for the main text and that it should be kept in the methods section. We have removed this from the main text.

*Later in this paragraph it would help to rewrite to clarify what tissue-associated groups are? "..the existence of tissue-associated groups converging on particular tissues or cell types" Groups of what?*

**Response:** We have revised this sentence as follows:

"Clustering analysis of the enrichment results revealed the existence of groups of phenotypes with significant associations for specific tissues or cell types.”

*Later you imply you mean groups of phenotypes but then to me "tissue associated phenotypes" is unclear. Every phenotype is associated with a tissue.*

**Response:** In this paper, we evaluated associations between GWAS results and cell type- or tissue specific regulatory regions using an unsupervised, aggregate statistical approach based on genomic data. In our analysis, not every phenotype with GWAS results showed a significant tissue-specific association. This does not necessarily mean that the phenotype is not associated with a tissue, only that the GWAS results for that phenotype do not show any significant enrichment for particular cell types or tissues. We have removed the phrase “tissue associated phenotypes” from the manuscript for clarity.

*The groups are important to explain because otherwise this result seems weird and obvious and without meaning: "For example, brain was associated with cognitive performance,…"*

**Response:** All of these significant tissue-specific associations were obtained purely by unsupervised statistical analysis in this paper. We did not input any of knowledge of disease aetiology to fine-tune any of these associations. Some of the results might seem obvious or confirmatory, but they are important to show, because they confirm that our analysis is performing as expected.

We agree with reviewer 1 that there are too many examples throughout the text. We have amended this text to:

" For example, this analysis showed that GWAS findings for QT interval were enriched for DNase I hotspots in heart tissue, whereas GWAS results for red blood cell traits were enriched for DNase I hotspots in hematopoietic stem cells. Several of these associations were previously known, but we uncovered many new associations, including, among others, for hair colour, which was enriched for melanocyte DNase I hotspots, and for waist circumference, which was enriched for mesenchymal stem cell DNase I hotspots.”

*This sentence is both dense and potentially repetitive making it less clear: "Thus, we demonstrate that FORGE2 can systematically link a wide variety of phenotypes to accessible chromatin elements specific to particular tissues and cell types, confirming previous findings [4], and also revealing previously unreported links between GWAS phenotypes and epigenomic marks for certain cell types and tissues." If I understand what you are saying I suggest rewriting as follows: "Thus, we demonstrate that FORGE2 can systematically link a wide variety of GWAS findings for associations between SNPs and phenotypes to accessible chromatin elements specific to particular tissues and cell types, providing functional confirmation of the GWAS findings…"*

**Response:** Thank you for this very helpful suggestion. We have amended the text to:

"Thus, we demonstrated that FORGE2 can systematically link a wide variety of GWAS associations to DNase I hotspots (or accessible chromatin elements) specific to particular tissues and cell types, providing functional confirmation of previous findings [4], and also revealing previously unreported links between GWAS phenotypes and epigenomic marks for certain cell types and tissues.”

*It would be useful to include the input GWAS summary dataset that you used for all the example as a supplementary file. That way users could attempt to run that and see if they get the same results as you and then they will know how to interpret results that they might get.*

**Response:** All input SNP lists, output analyses and figures have been uploaded to the FORGE2 Google Drive directory and are accessible from the following link: <https://drive.google.com/drive/folders/10GYaZQj_dF8FVj8LzMMBTlf1MwNBIIpU?usp=sharing>

*For the example analysis, when you say "supplementary table 2 of study" do you mean that you have reproduced the table 2 from the referenced paper? Or is that data already in one of your supp tables? Make that clear.*

**Response:** We meant the supplementary table from the referenced paper, but we agree that this is confusing. We have revised this sentence as follows:

"This study reported a list of 25 distinct variants with suggestive associations with MPNs (p-value <10^-6^) [17].”

*If not already there, you should include the actual file that you input into FORGE2 to run this example as a supplementary file in this paper.*

**Response:** Following reviewer indications, we have added the list of SNPs used in the MPN analysis to the Google Drive directory: <https://drive.google.com/file/d/18HVoGZka5b6I7nxCFihexSQx7zdaAj3u/view?usp=sharing>.

*Also, in supp tables 1 and 2 what are those Zscores and P values for? Can you add a supp file that defines the columns?*

**Response:** We have clarified our methods to describe the statistical tests used to estimate enrichment:

“To test for enrichment (higher overlap than expected), FORGE2 first computes an overlap score for a SNP set (the sum of all overlaps of that set) against epigenomic track data across a range of cell types and tissues. For each epigenomic track sample, the overlap score of the input set is then compared to the overlap scores of 1000 background SNP sets that FORGE2 generates. SNPs from these 1000 background sets are selected for similarity to the input SNP set, via matching decile bins for minor allele frequency (MAF), distance to transcription start site (TSS) and GC content. A binomial test is used to compare the overlap score of the input SNP set with the overlap scores of the 1000 background sets. This is repeated across multiple cell/tissue samples, until all samples have been analyzed. The p-values from the binomial tests for all cell/tissue samples tested are then adjusted for multiple testing via the Benjamini-Hochberg (BH) method [39], generating q-values as a result. BH is applied at a sample level, including all samples and p-values. Enrichments at a q-value<0.01 are considered significant. Alongside q-values, corresponding uncorrected p-values and Z-scores are also provided by FORGE2.”

Supplementary Tables 1 and 2 are Supplementary Tables 11 and 12 in the revised manuscript. We have also added the following text in supplementary tables 11 and 12 defining each column of the output, following reviewer indications:

FORGE2 enrichment Zscore: Z-score of the overlap count of the input data versus the background.

FORGE2 enrichment Pvalue: P-value of the overlap count of the input data versus the background.

Cell: cell type in which the enrichment is being computed.

Tissue: tissue annotation according to either ENCODE, Roadmap Epigenomics, BLUEPRINT, or BioSamples.

Datatype: type of epigenomic track analysed.

File: file name for the specific epigenomic track analysed.

Probe: The specific SNPs that overlap epigenomic tracks in that file.

Accession: GEO accession number of the sample or dataset.

FORGE2 enrichment Qvalue: Q-value of the overlap count of the input data versus the background.

Finally, we have also changed column headers to "FORGE2 enrichment Zscore, FORGE2 enrichment Pvalue and FORGE2 enrichment Qvalue".

*Just a few other examples of where the language is confusing - not exhaustive*

*In the section on "Relationship between datasets". When you say "we clustered our GWAS results". Do you really mean GWAS results? To me that means the GWAS results you downloaded from the databases. I think you don't mean that but rather the results from running FORGE2 on those.*

**Response:** This section has been revised as follows:

"Given the interrelationship between DNase I hypersensitive regions, histone mark broadPeaks and HMM chromatin state regions, we sought to compare the FORGE2 results across these different data types. Clustering of the FORGE2 results revealed large-scale substructure with segregation into 5 main groups, ranked on the number of associated GWAS phenotypes identified by each group (**Figure 5**).”

*Methods description*

*I like the Figure legend for Supp figure 1 as a summary of the Methods. But a few things could be clarified to make it easier to follow. "over 454 samples" confuses me. What 454 samples?*

**Response:** We apologize for the confusion. For DNase I hotspots, there are a total of 454 cell-type/tissue samples available for analysis, summing across the BLUEPRINT, Roadmap Epigenomics and ENCODE data. However, the number of cell/tissue samples varies by epigenomic dataset. When running FORGE2, the user can specify the epigenomic tracks to be used in the analysis. We have added the number of tissues/cell type samples from each epigenomic dataset to the methods. For simplicity, in Supplementary Figure 1, we gave the total number of samples included in FORGE2 analysis for DNase I hotspots.

*What do you mean by "using a similar array background". What array?*

**Response:** Apologies, this was an erratum. The text has been amended to "using background SNPs".

*In the corresponding text of the methods section, when you say that "FORGE2 takes a set of input SNPs (typically a set of RSIDs) and computes their overlap score (the sum of all overlaps) against epigenomic track data across a range of cell types and tissues." can you clarify how you define overlap and how you define this so that you can calculate the score for overlap with a tissue or cell type? This is probably very clear to you but it is not obvious.*

**Response:** We have clarified this in the "FORGE2 database generation" text (Methods): " Bedops [38] was used to overlap epigenomic track bed files with SNP positions, where an overlap indicates identical coordinate position for the SNP and epigenomic track at a given base pair.”

*Outputs section*

*You say that various pdfs come out of running FORGE2. You ran the example using an MPN GWAS dataset. I don't know how many pdfs come out of FORGE 2. Could you possibly make a supplementary file with all of those with annotation regarding how to interpret them? Can you give a couple of examples of key pdfs that come out of FORGE2 with some annotation of how to interpret them? This would be very useful. I assume it is more than just Figure 6 a and b.*

**Response:** We have amended text in the "Outputs" Methods section as follows:

"FORGE2 returns several different outputs, including charts, tables, original raw text files and R code for plotting. Tables and charts are also generated in interactive format to allow for an intuitive exploration of cell type- and tissue-specific enrichment results. Specifically, FORGE2 outputs: a web-based interactive table, a web-based dimple (http://dimplejs.org) d3 interactive graphic using rCharts, a tab-separated values (TSV) file, a PDF file, R code source files for generating the charts and the table, as well as a compressed file with the input data. FORGE2 provides output similar to FORGE [10], eFORGE v1.0 and eFORGE v2.0 [35] [36][40], and additional information can be found in those references as well as in a recent epigenome-wide association study (EWAS) [41].”

Figure 6 a and b are examples of FORGE2 PDF output. The interactive graphic is the same except for the interactive response. The other outputs are table outputs and we have added examples of these in Supplementary tables 11 and 12.

As indicated above, all input SNP lists, output analyses and figures have been uploaded to the FORGE2 Google Drive directory and are accessible from the following link: <https://drive.google.com/drive/folders/10GYaZQj_dF8FVj8LzMMBTlf1MwNBIIpU?usp=sharing>. These files include all charts and tables for each of the GWAS catalogue phenotypes analyzed. We also include full descriptions on standard analysis, output and input files in FORGE2 help (<https://forge2.altiusinstitute.org/?help>) and documentation (<https://forge2.altiusinstitute.org/?documentation>) pages.

*False positive rate testing*

*You indicate that the original FORGE used Bonferroni but this one uses FDR<0.05. Can you give a reason for the change? Did you get feedback on this point?*

**Response:** The original FORGE used a tissue (not sample or cell type) level Bonferroni correction (e.g. if there are 24 tissues but 299 samples then the p-value cutoff is 0.05/24 not 0.05/299). Because of this, FORGE could only compute tissue-specific enrichments and not enrichments at the level of specific cell types or samples. eFORGE v1 and later v2, and FORGE2 use false-discovery rate (FDR) to correct for multiple testing at a sample-specific level. By using a sample-specific FDR correction, we can compute enrichments at the level of specific cell types, where samples clearly identify with one cell type (e.g. CD8+ T cells). In addition, in the cases where tissue samples present a heterogenous mixture of cell types, where some samples present a higher proportion of particular cells (e.g. immune cells) and other samples presenting a lower proportion these cells (e.g. an intestinal sample with mucose-associated lymphoid tissue or MALT, compared to another intestinal sample without MALT), we can correct for each sample separately. This allows us to compute enrichments at the level of each individual sample and test performed and to detect sample-specific tissue-level enrichments. We have shown that this provides a low false positive rate, while still retaining a good capability for detecting target cell types associated with disease mechanism.

To reduce confusion, we have removed the reference to FORGE and revised this section and the previous one as follows:

“A binomial test is used to compare the overlap score of the input SNP set with the overlap scores of the 1000 background sets. This is repeated across multiple cell/tissue samples, until all samples have been analyzed. The p-values from the binomial tests for all cell/tissue samples tested are then adjusted for multiple testing via the Benjamini-Hochberg (BH) method [39], generating q-values as a result. BH is applied at a sample level, including all samples and p-values. Enrichments at a q-value<0.01 are considered significant. Alongside q-values, corresponding uncorrected p-values and Z-scores are also provided by FORGE2.

**False positive rate testing**

To evaluate the false positives rate, we ran > 27 million random input SNP tests with FORGE2, including 1000 sets of 5, 10, 15, 20, 30, 40, 50, and 100 SNPs. This analysis showed a low level of false positives (7.6 for every 100,000 tests at a BH-corrected p-value<0.01). False Discovery Rate (FDR) correction was implemented in a similar way to the eFORGE tool, which previously implemented FDR for the detection of cell type-specific and tissue-specific enrichment signal [35][36][40]. ”

**Second round of review**

**Reviewer 1**

The authors have addressed my concerns.

There may still be challenges with readability of major figures that the editorial staff will want to work with authors to address.

**Reviewer 2**

The responses to my comments are quite thorough and satisfactory. The revised manuscript is much clearer now.

My only question is whether the link to the Easyzoom folder with the figures is stable and archived so that this same link will work in 5 years. Sometimes papers have links that clearly have expired or the site has gone away. Would depositing in some site that generates a DOI be an option. DOIs don't go away. But if this is not an issue with Easyzoom, there is no problem.

**Authors’ response to reviewers**

We thank the editors and reviewers for their many comments and formatting suggestions to improve our paper. Please find enclosed the revised manuscript addressing all of these comments. We have addressed all the comments and updated the manuscript accordingly.
